# Guest‐Induced Activation of Multicolor Photoluminescence in Naphthalene Bisimide Liquid Crystals

**DOI:** 10.1002/adma.202520184

**Published:** 2026-02-25

**Authors:** Johannes Nowarra, Swadhin Garain, Matthias Stolte, Frank Würthner

**Affiliations:** ^1^ Institut für Organische Chemie and Center for Nanosystems Chemistry (CNC) Universität Würzburg Würzburg Germany

**Keywords:** aromatic bisimides, charge‐transfer, emission tuning, liquid crystals, RTP, TADF

## Abstract

Achieving color‐ and lifetime‐tunable emission in processable materials remains challenging, as most of the available advanced materials systems exist only in impractical solutions or hard‐to‐process crystals. Liquid crystals (LCs) represent a promising platform due to their processability, though previous work has mainly controlled emission color by external fluorophore doping. In this work, we present a supramolecular strategy to activate and tune both photoluminescence (PL) color and lifetime in naphthalene bisimide (NBI) LCs by embedding electron‐rich guests. The electron‐deficient NBIs form columnar hexagonal mesophases stabilized by nanosegregation of aromatic cores and aliphatic side chains, providing a suitable environment for charge‐transfer (CT) interactions. Incorporating electron‐rich polycyclic aromatics, carbazole derivatives, or square‐planar Pt(II) complexes yields emissive CT states with tunable emission from visible to near‐infrared (511–685 nm), covering short‐lived fluorescence to long‐lived delayed luminescence of coexisting thermally activated delayed fluorescence (TADF) and room‐temperature phosphorescence (RTP). Co‐assembling two complementary NBI hosts with one guest enables an energy‐transfer cascade that simultaneously supports green and red dual emission of fluorescence and TADF. This additive‐induced activation and host‐mixing approach greatly broadens the luminescence range accessible in LCs, combining tunable photophysical properties with intrinsic processability for next‐generation optical encoding and security labeling technologies.

## Graphical abstract

Supramolecular host–guest design in naphthalene bisimide liquid crystals activates charge‐transfer emission and enables continuous tuning of color (511–685 nm) and lifetime, spanning prompt fluorescence, TADF, and RTP. Electron‐rich guests and Pt(II) complexes modulate emissive states, while co‐assembled NBI complexes create an energy‐transfer cascade for dual emission. The processable LC platform targets advanced optical encoding and security labeling.

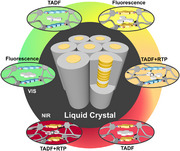

## 1 Introduction

Liquid crystals (LCs) have become indispensable in today's world. Due to the unique properties of mobility as in liquids, combined with the molecular order of crystals, they found application in displays and various technologies [1, 2, 3, 4, 5]. For the latter, direction‐dependent optical, electrical, and mechanical responses enabled advanced functions like smart materials and reconfigurable devices [4, 6, 7, 8, 9, 10, 11, 12, 13, 14, 15]. Furthermore, LCs have emerged as promising platforms for security applications, offering sophisticated anti‐counterfeiting solutions through switchable optical properties, structural colors, polarization‐dependent luminescence, and stimuli‐responsive behaviors, enabling multi‐mode authentication systems [16, 17, 18, 19, 20]. What has so far been much less explored in LC systems is the deliberate modulation of photoluminescence (PL) involving triplet states through tailored supramolecular interactions. In conventional fluorophore LC systems, luminescence is typically achieved by physical doping of emissive chromophores into a non‐emissive LC hosts or by covalent attachment of mesogenic units to chromophores [21, 22, 23]. While these strategies can yield luminescent LC materials, they are limited in adjustability when it comes to accessing multiple states of emission. As a result, the rational activation of exciplex emission, thermally activated delayed fluorescence (TADF), and room‐temperature phosphorescence (RTP) within a single LC host chromophore remain largely unexplored. In contrast, recent studies on co‐crystals and cyclophanes have demonstrated that precisely engineered supramolecular interactions can effectively modulate triplet‐state energetics and emission pathways, highlighting a conceptual gap that has not yet been adequately addressed in LC materials [24, 25, 26, 27, 28, 29].

Here, we demonstrate supramolecularly activated and tunable PL of two LC naphthalene bis(dicarboximide) (NBI) host materials through embedment of different guest molecules into their mesophase. We want to emphasize that in contrast to literature known systems for which luminescence in doped LC or LC mixtures has been realized by doping luminescent dyes into a nematic or smectic LC host, the here reported luminescence emerges from the LC host itself, being activated by the respective guest molecules. As we will show, this approach involving triplet states affords tunability of luminescence color and lifetime over a broader range and a rarely achieved continuity than accomplished in traditional luminescent LC materials [21]. To demonstrate the concept, we selected NBIs forming columnar hexagonal (Col_h_) mesophases as matrices and a variety of electron‐rich polycyclic aromatic hydrocarbons (PAHs) and Pt(II) complexes for activation of luminescence by means of through‐space intermolecular charge‐transfer (CT) interactions [30, 31]. By proper selection of the guest, the emissive state of the LC host can be controlled, resulting in emission tuning from the visible to the near infrared spectral range and covering both short‐lived fluorescence up to long‐lived delayed luminescence of coexisting thermally activated delayed fluorescence (TADF) and room‐temperature phosphorescence (RTP). Building on our findings of CT LC mixtures, we co‐assembled the two complementary NBI chromophore hosts with one guest molecule to create a mesophase framework for an energy‐transfer cascade, enabling simultaneous green and red emission for dual emission and smooth tuning of both color and lifetime in the LC state of matter.

## 2 Results and Discussion

### 2.1 LC Hosts and Guest Molecules

**FIGURE 1:**
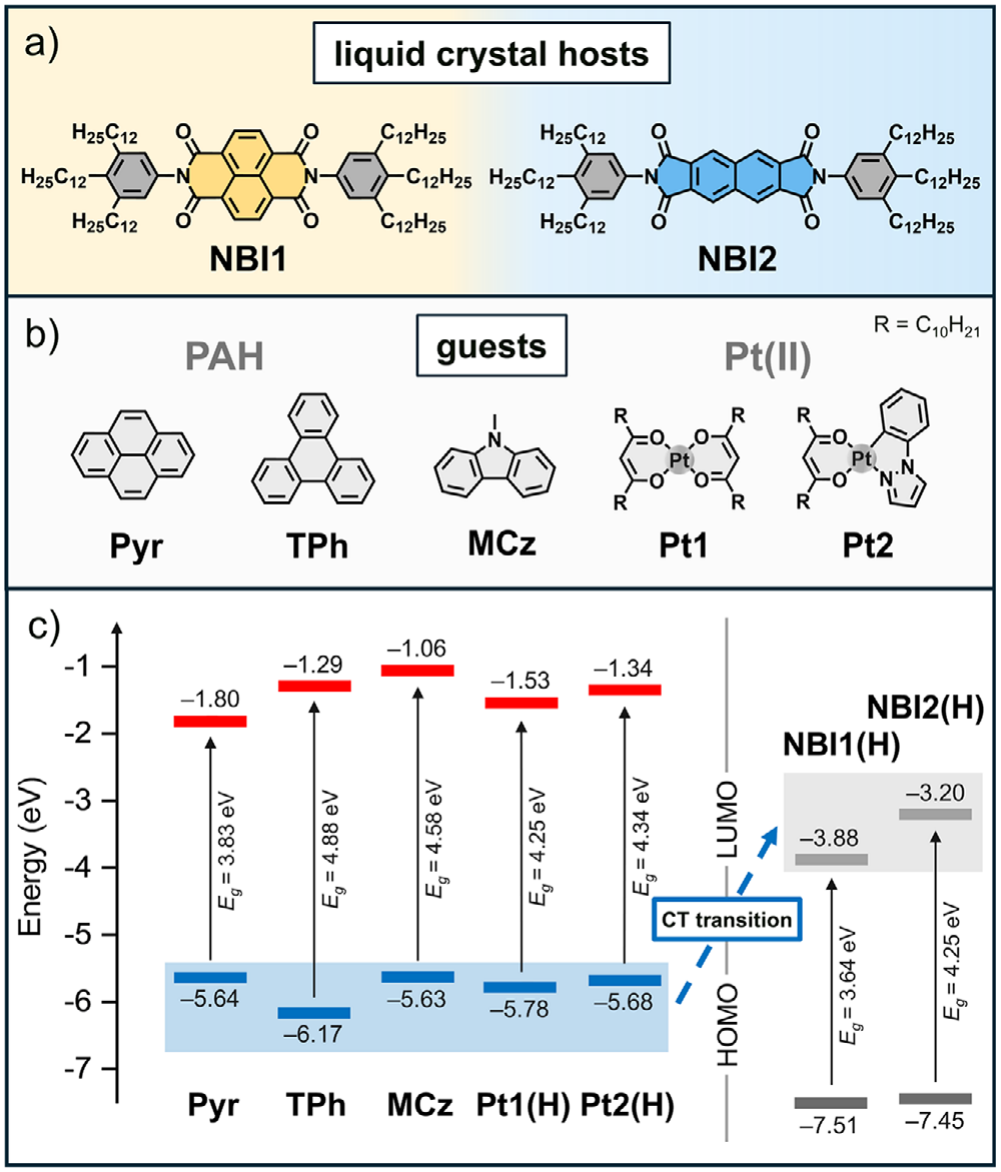
(a) Chemical structures of LC aromatic bisimides **NBI1** and **NBI2** with trialkyl phenyl imide substituents used as hosts and (b) of guests molecules based on PAHs pyrene (**Pyr**), triphenylene (**TPh**), *N*‐methyl carbazole (**MCz**), and Pt(II) complexes **Pt1** and **Pt2**. (c) Calculated energy‐level diagram showing the HOMO (blue, dark gray) and LUMO (red, light gray) energy levels and resultant energy gaps *E*_g_ of guest molecules **TPh**, **Pyr**, **MCz**, homoleptic Pt(II) complex **Pt1(H)**, heteroleptic Pt(II) complex **Pt2(H)** as well as of the LC host molecules **NBI1(H)** and **NBI2(H)**. The imide substituents of the NBIs and the alkyl chains of the Pt(II) complexes were replaced by H atoms.

Based on our earlier work on cyclophane‐based supramolecular host–guest complexes and co‐crystals, we designed two mesogens based on electron poor naphthalene‐1,8:4,5‐bis(dicarboximide) (**NBI1**) and naphthalene‐2,3:6,7‐bis(dicarboximide) (**NBI2**) cores, which differ in the position of their respective imide substituents [32, 33, 34, 35]. LC properties were induced through the attachment of well‐established mesogenic units consisting of aliphatic dodecyl chains in imide position [36, 37]. The NBIs were synthesized by imidization reactions from the corresponding naphthalene bisanhydrides with the mesogenic unit 3,4,5‐tridodecyl aniline in glacial acetic acid (Figure 1a). Notably, several 1,8:4,5‐NBI‐based LCs are already described in literature, whereas 2,3:6,7‐NBI‐based LCs have not been reported yet [38, 39, 40, 41, 42, 43].

The shape‐complementary host and guest molecules were selected based on a theoretical design principle in which a high HOMO level (i.e., good donating strength) of the donor and a deep LUMO level (i.e., good accepting strength) of the acceptor are expected to promote orbital mixing and thereby facilitate CT state formation (Figure 1). Accordingly, **NBI1** and **NBI2**, which possess deep LUMO levels (*E*_LUMO_ = −3.88 and −3.20 eV for **NBI1** and **NBI2**, respectively), are energetically well suited to form emissive CT states with all selected donors (*E*_HOMO_ = −5.63 to −6.17 eV), as illustrated in Figure 1c. As electron rich guests (Figure 1b) we chose the PAHs pyrene (**Pyr**), triphenylene (**TPh**), and *N*‐methyl carbazole (**MCz**), and two newly designed Pt(II) complexes, one homoleptic and one heteroleptic, with highly reduced melting points to roughly match the temperature of mesophase formation of the NBI hosts. The first complex platinum(II) bis(tricosane‐11,13‐dione) (**Pt1**) is a congener of Pt(acac)_2_ sensitizer (utilized by us previously for inducing RTP of NBI) [27, 28] bearing extended alkyl chains. **Pt1** was synthesized in a solid‐state reaction involving ball‐mill grinding. The second complex platinum(II) (1‐phenylpyrazole)(tricosane‐11,13‐dione) (**Pt2**) is heteroleptic with an extended π‐surface bearing a 1‐phenylpyrazole ligand and was synthesized by ligand exchange in solution (see Supporting Information for details). All guest molecules are crystalline in nature and do not form mesophases. All new molecules were characterized by ^1^H and ^13^C nuclear magnetic resonance (NMR) spectroscopy, high‐resolution mass spectrometry (HRMS), UV–vis and PL spectroscopy (see Supporting Information for details). The solid materials were further studied by differential scanning calorimetry (DSC), polarized optical microscopy (POM), and X‐ray scattering (XRS) as discussed in the following article.

### 2.2 Characterization of Pristine Materials

**FIGURE 2:**
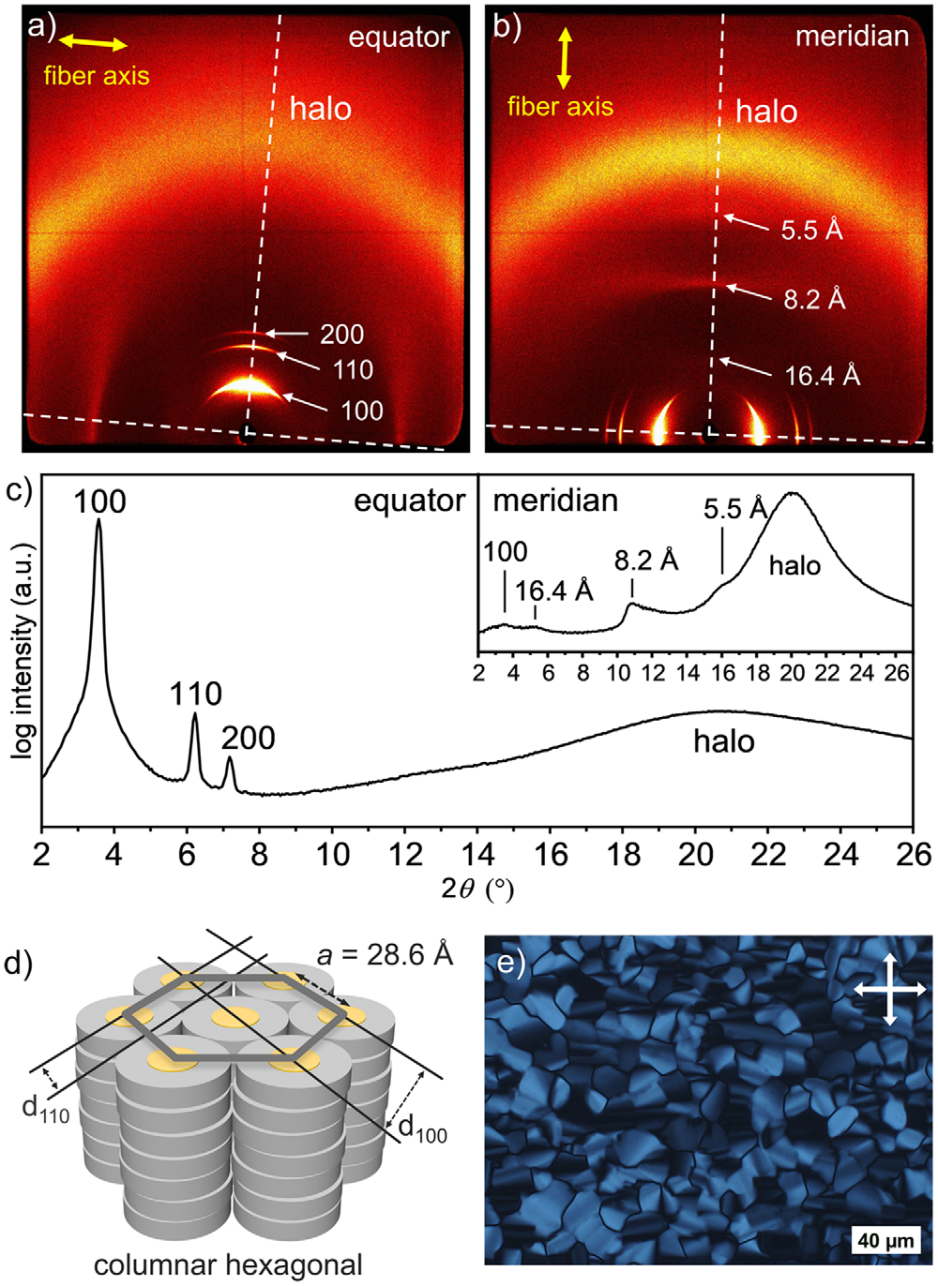
WAXS patterns of **NBI1** at 25°C of (a) a lying fiber and (b) a standing fiber. The direction of the fiber is indicated by yellow arrows, and the position of the equator and meridian are indicated with white dashed lines. (c) Integrated intensities along the equator and meridian (inlay) of the WAXS pattern. (d) Schematic disordered Col_h_ phase showing the self‐assembly of **NBI1** with lattice parameter (*a*) and Miller‐indexed lattice planes (d_hkl_). (e) POM image of **NBI1** with crossed polarizers at 25°C upon cooling from the isotropic liquid, displaying a pseudo focal fan‐shaped texture characteristic for a columnar phase.

The thermotropic properties of the NBI hosts were studied by DSC, POM, and XRS. Investigations by DSC and POM with a scan rate of 10 K min^−1^ revealed broad range LC behavior for both NBIs from −20°C up to the clearing into the isotropic liquid at 59°C for **NBI1** and 78°C for **NBI2**, respectively (Figure S1). The DSC peaks corresponding to the transition from the LC phase to the isotropic liquid show rather small enthalpies of around 3 kJ mol^−1^ (**NBI1**) and 4 kJ mol^−1^ (**NBI2**), indicating mesophases of low order (Figure S1). Notably, we found a significantly higher enthalpy value for **NBI1** at 46°C in the first heating run, indicative of a crystalline phase. This peculiarity indicates that the mesophase in this case is only metastable, which was further investigated with XRS and will be discussed later. In POM investigations both NBIs showed a high viscosity at ambient conditions that decreased with higher temperature, with **NBI1** being more viscous than **NBI2**. Upon cooling from the isotropic liquid both NBIs formed birefringent pseudo focal conic fan‐shaped textures that are indicative of Col_h_ LC phases (Figure 2; Figures S2 and S3) [44]. The textures did not change significantly with decreasing temperature, which is in accordance with the DSC traces showing one broad phase.

Wide‐angle X‐ray scattering (WAXS) measurements of aligned fibers at 25°C confirm Col_h_ LC phases for both NBIs (Figure 2; Figures S4 and S5). The Col_h_ LC materials can be aligned by extrusion to produce fibers [45]. In our case, this extrusion orients the columns in line with the fiber direction and the π‐surfaces of the aromatic cores within the columns perpendicular to the fiber direction. The integrated intensities of the equatorial signals, measured perpendicular to the fiber axis, show three main reflexes and a broad halo for both NBIs. The most dominant peaks are located at 24.8 and 27.6 Å for **NBI1** and **NBI2**, respectively, followed by two less intense peaks that can be assigned to the 100, 110, and 200 reflections of the Col_h_ phases with the lattice parameters *a* = 28.6 Å for **NBI1** and *a* = 31.8 Å for **NBI2** (Table S1). **NBI2** also shows a reflex that can be assigned to the 300 plane. Both NBIs have a diffuse halo in the wide‐angle region resulting from scattering by disordered liquid‐like alkyl chains [46].

While in this case the equator of aligned fibers is attributable to the intercolumnar distances, the meridian reveals intracolumnar order. We found peaks on the meridian for both NBIs that could be correlated to a layered organization in the columns. **NBI1** has the most intense peak at 8.2 Å that may originate from a stack of three NBIs (trimers) (Figure 2b,c). Both NBIs have a diffuse halo in the wide‐angle region of the meridian without a peak that could be assigned to a specific *π–π* distance (Figure 2c; Figures S4 and S5). The absence of discrete peaks suggests the presence of more mobile columnar disordered phases without distinct molecular spacings [37]. This observation is not unusual as disk‐shaped mesogens with small aromatic cores lack sufficiently strong *π–π* interactions to attain highly ordered arrangements compared to those with larger cores [37, 47]. This observation is also in accordance with the low enthalpy values of the clearing for both NBIs. For **NBI1**, in addition to our observations in the DSC traces, i.e. distinct peak during the initial heating run, we also noted a gradually developing opaque appearance over time. Thus, we investigated the stability of both NBIs for twenty days with WAXS measurements, which revealed that the initial disordered Col_h_ LC phase of **NBI1** is lost after one day, while the Col_h_ LC phase of the newly described **NBI2** remains stable and can be classified as enantiotropic (Figures S6 and S7) [48].

Next, we studied the optical properties of **NBI1** and **NBI2** in solution and in their mesophases. The UV–vis absorption spectra of the two NBIs in CHCl_3_ solution are similar to those of known derivatives [26, 30]. Thus, **NBI1** shows a more intense S_0_→S_1_ absorption band with well resolved vibronic progression and an absorption maximum (*λ*_max_) at 380 nm, while the lowest energy transition of **NBI2** is of weak intensity around 376 nm aside a dominant rather broad absorption band at 308 nm (Figure S8a). The absorption spectra of the Pt(II) complexes in CHCl_3_ solution at 25°C are broad and more intense in the UV region below 300 nm with a first maximum at 348 and 315 nm for **Pt1** and **Pt2**, respectively (Figure S8b). Both bisimides and both Pt(II) complexes are non‐emissive in CHCl_3_ solutions. As thin films in the LC phase, **NBI2** showed a weak light green fluorescence with a broad unstructured emission band and a maximum (*λ*_em_) at 539 nm that we attribute to an excimer while the emission of **NBI1** is negligible [49]. For **NBI2** the major lifetime PL component is 1.4 ns, and the PL quantum yield (*Φ*_PL_) is lower than 1% (Figures S9 and S10). The Pt(II) complexes showed crystalline behavior in both POM and DSC investigations with melting points of 85°C and 76°C for **Pt1** and **Pt2**, respectively (Figures S11–S14, Table S2). In the solid state, **Pt1** is non‐emissive, and **Pt2** shows weak emission with the longest lifetime component of 3 µs and a *Φ*_PL_ lower than 1% (Figures S15 and S16).

### 2.3 Liquid Crystalline Mixtures

**FIGURE 3:**
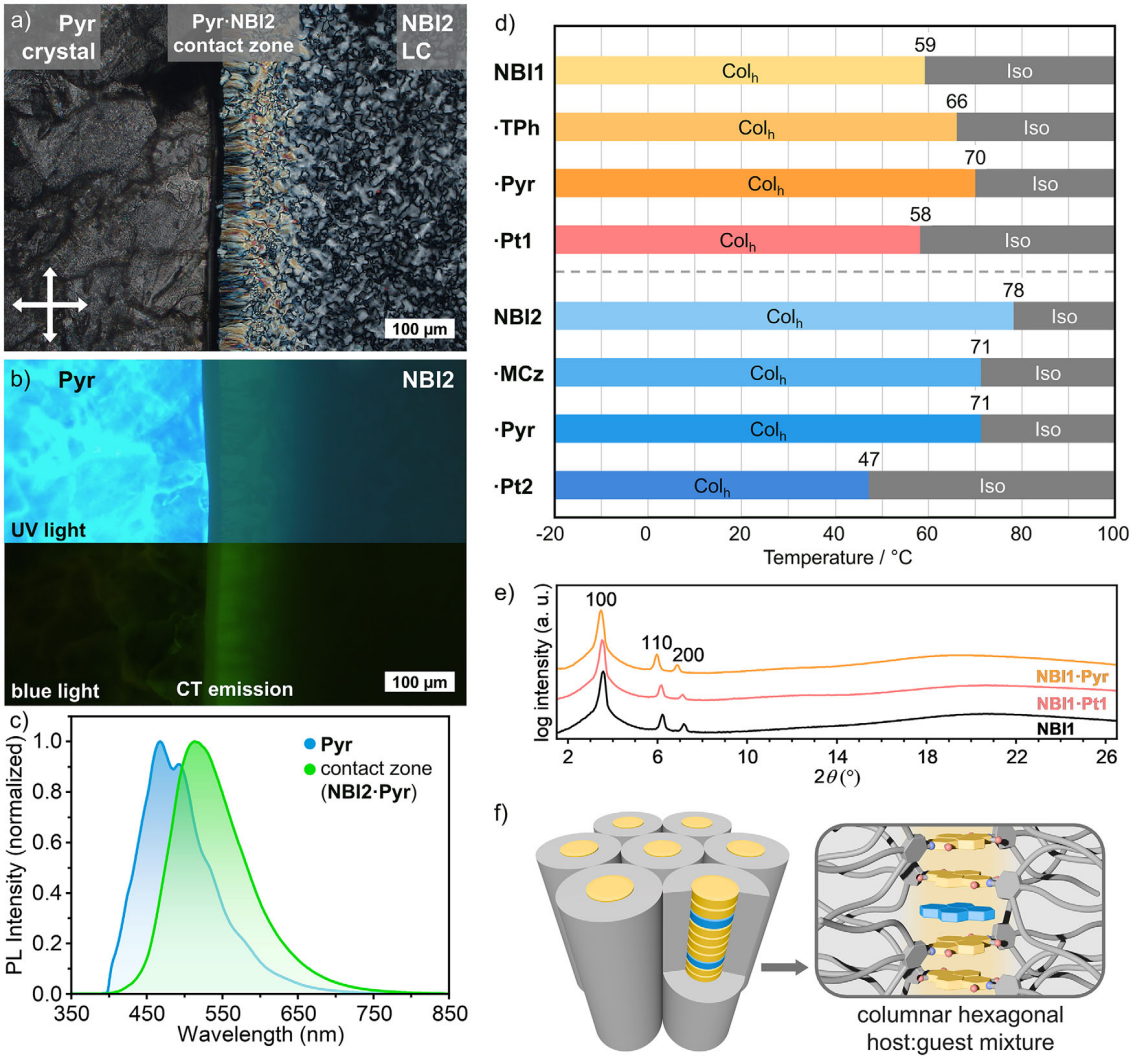
POM images of the contact experiment of **NBI2** host and **Pyr** guest. Recorded at ambient conditions (a) in transmission mode with crossed polarizers and (b) in reflection mode under UV light (top, *λ*_ex_ = 350–390 nm, *λ*_detection_ > 400 nm) and blue light (bottom, *λ*_ex_ = 460–500 nm, *λ*_detection_ > 520 nm) excitation. (c) PL spectra (not corrected) of **Pyr** (blue) and of the **NBI2·Pyr** contact zone (green) under UV light irradiation of the sample shown in (b). (d) Temperature‐dependent phase transition behavior of neat **NBI1** and **NBI2**, as well as the mixtures with the guests **TPh**, **Pyr**, **MCz**, **Pt1**, and **Pt2**, showing the clearing points upon heating; Col_h_ = columnar hexagonal; Iso = isotropic liquid. (e) Integrated intensities of the exemplary WAXS pattern at 25°C of the aligned fiber of **NBI1** (black), **NBI1·Pt1** (red), and **NBI1·Pyr** (orange). (f) Schematic presentation of the Col_h_ LC phase of **NBI1** with embedded guest molecule **Pyr** (blue).

Based on their frontier‐orbital energy levels (Figure 1c), all guest molecules are expected to form CT complexes with the two electron‐poor NBI hosts through interactions between the guest HOMO and the NBI host LUMO [31, 34, 35]. Moreover, the design of the study incorporates both conventional CT interactions with PAHs as well as the heavy‐atom effect (HAE) of Pt(II) complexes, we target mixtures with emission from singlet and triplet states [27, 28]. Our first experiment for LC mixtures involved examining contact probes using POM complemented by PL studies under UV and blue light irradiation within the same setup [50, 51]. This experiment can reveal possible emitting CT interactions when substances come into contact. For both NBIs, we obtained emission colors ranging from green over yellow to red for the available series of guest molecules (Figure 3a–c; Figures S17–S26).

Thus, the contact zones showed green emission for **NBI2·Pyr** (Figure 3a–c), light green emission for **NBI2·MCz**, yellow emission for **NBI1·TPh**, orange emission for **NBI2·Pt2**, red emission for **NBI1·Pyr** and **NBI1·MCz**, and dark red emission for **NBI1·Pt1** and **NBI1·Pt2** (Figures S17–S26, Table S3). As the proportions of the mixture decrease on both sides of the contact zone, it can be deduced that the mixing ratio has no effect on the emission color. These experimental results confirm the successful LC donor‐acceptor design and demonstrate that emissive CT states can be generated within columnar LCs of both NBI hosts. As shown in Figure 3, depending on the excitation wavelength (*λ*_ex_), blue emission originating from crystalline pyrene (Figure 3b, **Pyr**; left) can easily be distinguished from PL originating from the host–guest complexation zone (Figure 3b, **NBI2**; right). The PL properties were investigated in more detail, as will be discussed later. While **NBI1·Pt2** displayed luminescence behavior similar to **NBI1·Pt1** but with substantially reduced emission intensity (Figures S23 and S27), **NBI1·MCz** exhibited only faint red luminescence (Figure S24). Given these limited photophysical responses, further investigation of their structural properties was not pursued.

Mixtures with a stoichiometric ratio of 5:1 for NBI:guest were found to exhibit LC behavior across all luminescent systems, and the best optical response (Table S4). Accordingly, we proceeded with this ratio throughout our subsequent study. To gain structural insights we investigated the thermotropic behavior of the mixtures in the same way as for the neat NBI hosts. All mixtures showed pseudo focal conic textures under POM investigations, indicating Col_h_ LC phases, and the mixtures could be aligned by shearing, resulting in linearly polarized luminescence (Figures S28–S33) [36]. DSC measurements revealed a broad range LC behavior from −20°C transitioning to the isotropic liquid at temperatures around the hosts clearing points (59°C **NBI1**, 78°C **NBI2**). The mixtures of **NBI1·TPh** and **NBI1·Pyr** exhibit higher clearing temperatures than the neat host **NBI1**, thereby suggesting that the guest molecules stabilize the LC phase by *π–π*‐stacking interactions with the **NBI1** core (Figure 3d; Figure S34). This co‐assembly reinforces the columnar organization, increasing the energetic barrier against disorder and extending the mesophase temperature range. In contrast for the mixtures of **NBI2·MCz** and **NBI2·Pyr** we recorded lower clearing points than the neat **NBI2** host, suggesting destabilization of the mesophase by the addition of guest molecules (Figure 3d; Figure S34d,f). This destabilization can be attributed to packing frustration as host–guest interactions in the columnar lattice are weaker than the host–host interactions of **NBI2**. The effect is most pronounced for **NBI2·Pt2**, which showed a strong decrease of the clearing point to 47°C (Figure 3d; Figure S34e). Importantly, both stabilization and destabilization effects are observed consistently upon heating and cooling, confirming that the altered phase behavior reflects intrinsic thermodynamic changes induced by the guest molecules rather than kinetic artifacts.

WAXS measurements showed that the structural properties of the LC hosts are not altered significantly, as only slight changes in the lattice parameters of the mixtures compared to the hosts are noticeable (Figure 3e; Figures S35–S40, Table S1). Thus, the disordered Col_h_ phase of the NBI hosts are preserved upon mixing with all guests, and it is reasonable to assume that the guest molecules are embedded between the π‐planes of the NBI host molecules in a statistical manner (Figure 3f) resulting from *π–π* and Pt–π stacking interactions, similar to that previously observed in cyclophanes or co‐crystalline materials (Figure 3f) [21, 22, 25, 26, 27, 28]. Based on this rationale, we expected also similar PL properties for the respective mixed LC phases, which will be investigated in the following.

Although the initial structural integrity of the host–guest mixtures is maintained upon mixing, the stability of the NBI hosts may influence the stability of the mixture, as in the case of **NBI1** the Col_h_ LC phase was lost after one day. We therefore conducted systematic stability studies focusing on the preservation of the mixture's Col_h_ phase and the luminescence profile. Mixtures containing **NBI2** showed good stability over time, whereas **NBI1** based mixtures exhibited pronounced degradation in both structural order and PL behavior, mirroring the behavior of the neat host (Figures S41,S42, Table S5). Consequently, the long‐term stability of the Col_h_ phase and luminescence of the mixtures are directly correlated with the intrinsic phase stability of the NBI‐based LC host. Notably, owing to the temperature‐dependent phase behavior of thermotropic LCs, such degradation can be reversed by thermal cycling, which restores not only the Col_h_ order but also the optoelectronic properties and emission characteristics of the present system (Figure S43).

### 2.4 Photoluminescence Properties of Liquid Crystalline Mixtures

**FIGURE 4:**
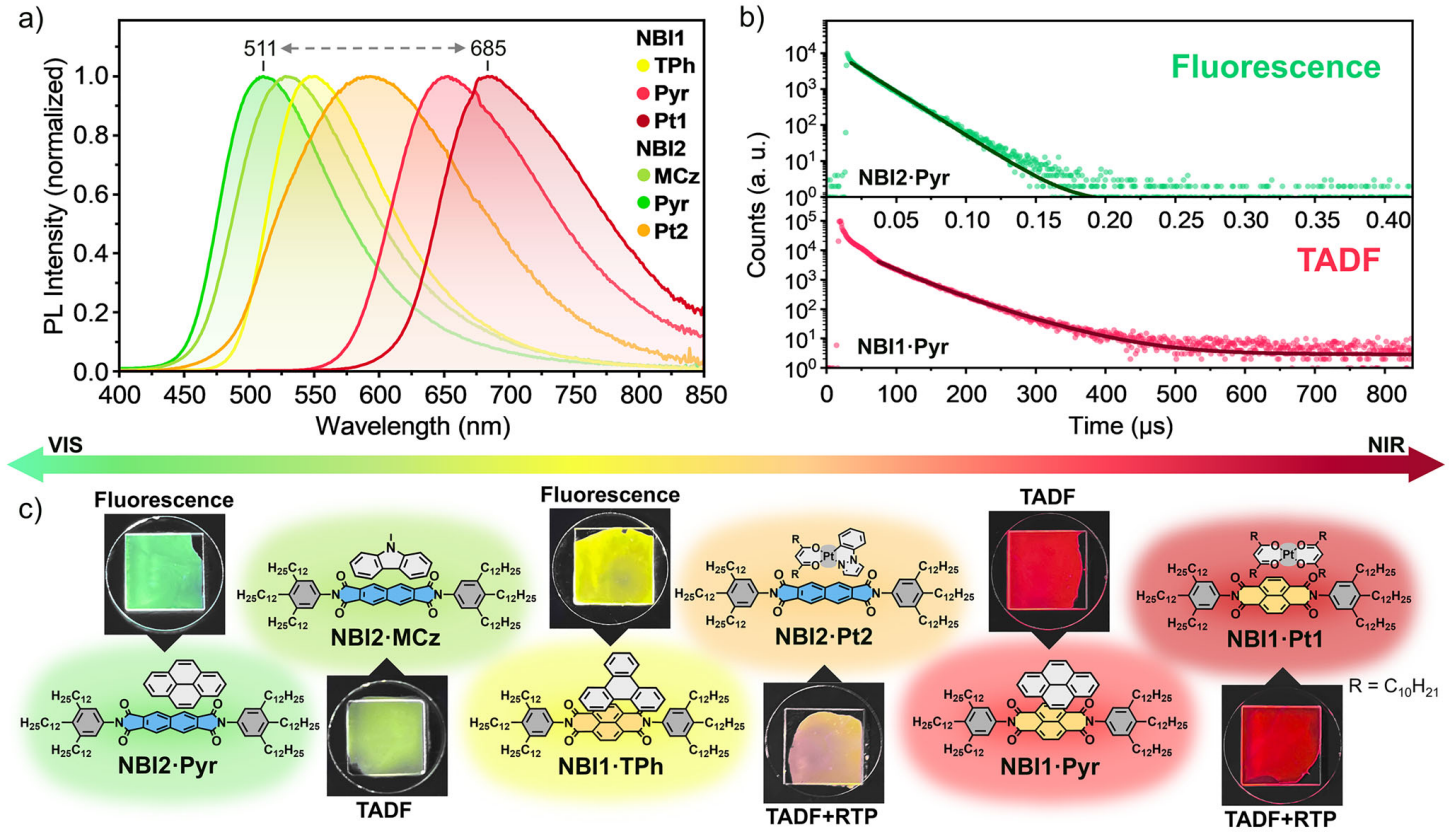
(a) Normalized PL spectra of the LC host:guest (5:1) mixtures as thin films on quartz substrates under ambient conditions. Spectra are displayed in the color of their respective emission; **NBI2·Pyr** (green, *λ*_ex_ = 350 nm), **NBI2·MCz** (light green, *λ*_ex_ = 350 nm), **NBI1·TPh** (yellow, *λ*_ex_ = 400 nm), **NBI2·Pt2** (orange, *λ*_ex_ = 400 nm), **NBI1·Pyr** (light red, *λ*_ex_ = 400 nm) and **NBI2·Pt1** (dark red, *λ*_ex_ = 400 nm). (b) Representative PL lifetime decays (symbols) along with the respective best fit (black line) of **NBI2·Pyr** (green) fluorescence and **NBI1·Pyr** (light red) TADF. (c) Images of substrates under UV light (*λ*_ex_ ≈ 400 nm, scale 10 × 10 mm^2^), also showing the complex with host **NBI1** (orange), **NBI2** (blue), and guests in gray formed by *π–π* or Pt–π interactions. The complexes are shown with their color of emission and assigned PL; fluorescence, thermally activated fluorescence (TADF), and room‐temperature phosphorescence (RTP).

For PL examination of the host:guest (5:1) mixtures, we drop‐casted them from CHCl_3_ solution onto heated quartz substrates and melted them to the isotropic liquid, before placing a cover glass to protect the sticky film. Then the samples were cooled to room temperature to form LC thin films. All mixtures of NBI host and guest emit from the LC state of matter at ambient conditions (Figure 4 and Table 1).

**TABLE 1:** Summary of the optical properties of the LC hosts **NBI1** and **NBI2**, as well as the mixtures **NBI2·Pyr**, **NBI2·MCz**, **NBI1·TPh**, **NBI2·Pt2**, **NBI1·Pyr**, **NBI1·Pt1** in 5:1 (NBI:guest) ratio as thin films on quartz substrates. The PL is classified as prompt fluorescence (PF), thermally activated delayed fluorescence (TADF), and room‐temperature phosphorescence (RTP).

Material	*λ*_em_ ^a^ (nm)	*Φ*_PL_ ^b^ (%)	*τ*_PF_ ^c^, ^d^ (ns)	*τ*_DF_ ^c^, ^d^ (µs)	*τ*_RTP_ ^c^, ^d^ (µs)	PL
**NBI1**	/	/	/	/	/	/
**NBI2**	539	< 1	5.0	/	/	PF
**NBI2·Pyr**	511	3	18	/	/	PF
**NBI2·MCz**	530	4	47	3.9	/	TADF
**NBI1·TPh**	549	2	18	/	/	PF
**NBI2·Pt2**	593	3	3.7	2.2	37	TADF+RTP
**NBI1·Pyr**	652	3	11	68	/	TADF
**NBI1·Pt1**	685	4	19	2.6	3.4^e^	TADF+RTP

^a^ Maximum of emission measured at ambient conditions.

^b^ PL quantum yield measured with absolute method at ambient conditions.

^c^ PL lifetimes measured by TCSPC excited either with an EPL laser diode, Agile laser or Xe flash‐lamp at ambient conditions.

^d^ Longest lifetime component given.

^e^ Coexistence of TADF and RTP. For more details see Supporting Information.

With the mixtures prepared from the two NBIs, coverage of a wide range of the visible spectrum could be achieved (Figure 4a,c; Figures S44–S49). The coverage starts with the green emission of **NBI2·Pyr** with a maximum at 511 nm, followed by light green emission of **NBI2·MCz** and the yellow emitting mixture **NBI1·TPh**. In the middle of the spectrum is the broad orange emission of **NBI2·Pt2**. The coverage is completed by the emissions of **NBI1·Pyr** in red and **NBI1·Pt1** in deep red with a maximum at 685 nm. The broad emission bands of **NBI1·Pyr** and **NBI1·Pt1** even extend into the NIR region. We found more and less pronounced CT bands in the excitation spectra of all mixtures in the spectral range from 400 to 650 nm, which verify the intermolecular CT interactions of closely stacked electron‐poor NBI and electron‐rich guests in the LC phase (Figures S44–S49). The *Φ*_PL_ of the LC mixtures are in the range of 2%–4% at ambient conditions (with a significant increase at low temperature), which provides a bright appearance of all samples under UV light excitation (Figure 4a,c, Table 1; Figure S50, Table S6). Since molecules in the LC state of matter are constantly in motion, the orbital overlap between NBI and the guest fluctuates, which reduces efficient CT interactions according to our previous study for a variety of Pt‐sensitized co‐crystals [28]. In addition, this dynamic environment could give substantial contribution to vibrational quenching, which together can account for the lower *Φ*_PL_ values compared to those of the more rigid crystalline systems. The low *Φ*_PL_ even at low temperatures for the systems that exhibit red emission (**NBI1·Pt1** and **NBI1·Pyr**) can be rationalized by the energy‐gap law [27, 28]. Notably, not only the overlap but also the distance between the molecules affects CT efficiency, and mobility opens non‐radiative decay pathways like internal conversion or vibrational relaxation.

Time‐resolved measurements revealed lifetimes of ∼18 ns for the green‐ and yellow‐emitting mixtures **NBI2·Pyr** and **NBI1·TPh** (Figure 4b; Figures S44 and S47). The short lifetimes of both systems are attributed to exciplex fluorescence from the ^1^CT states. The mixtures **NBI1·Pt1**, **NBI2·Pt2**, **NBI2·MCz**, and **NBI1·Pyr** exhibited both short‐ and long‐lived decays in the nanosecond and microsecond timescales, respectively (Figures S45–S48). The nanosecond components correspond to prompt fluorescence (PF) from the respective ^1^CT states, while the microsecond components indicate the involvement of triplet states, arising either from delayed fluorescence via reverse intersystem crossing (RISC) or phosphorescence associated with locally excited ^3^LE or ^3^CT states.

**FIGURE 5:**
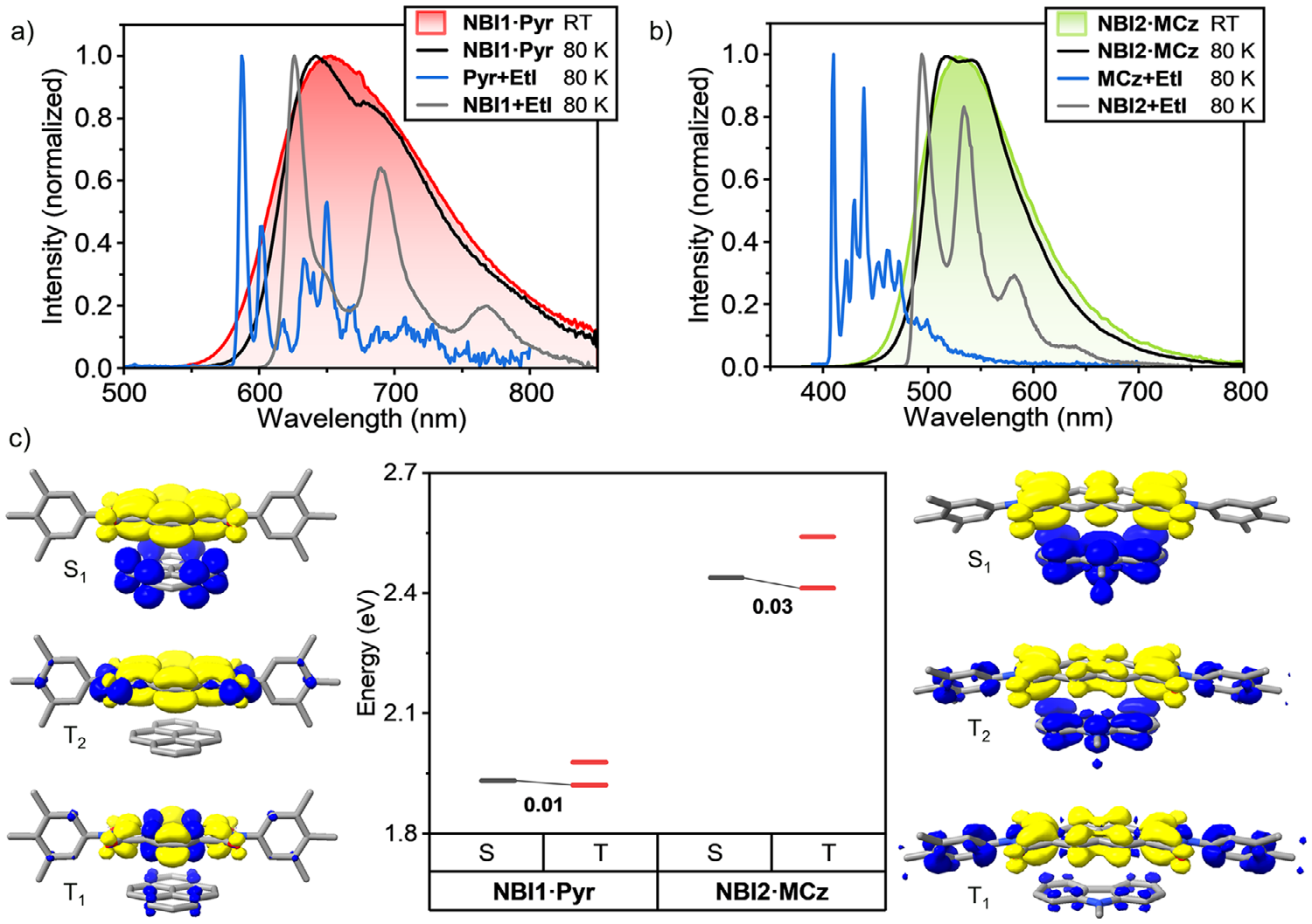
(a) Room and low‐temperature PL measurements of **NBI1·Pyr** and phosphorescence spectra of individual **NBI1** and **Pyr** sensitized with ethyl iodide (**EtI**). (b) Room and low‐temperature PL measurements of **NBI2·MCz** and phosphorescence spectra of individual **NBI2** and **MCz** sensitized with **EtI**. **NBI1** (in MCH, *c*_0_ = 1 × 10^−5^ m)**, NBI2** (in MCH, *c*_0_ = 1 × 10^−5^ m), **MCz** (MeTHF, *c*_0_ = 1 × 10^−5^ m), **Pyr** (in MeTHF, *c*_0_ = 1 × 10^−5^ m) with 20 wt.% **EtI** in respective solvent. (c) Optimized geometries, difference densities, and diagram of the calculated vertical singlet and triplet excited state energies of **NBI1·Pyr** (left) and **NBI2·MCz** (right). Difference densities shown at ±0.001 e/Bohr^3^ isovalue: loss of electron density with respect to the S_0_ state is shown in blue, while the gain is shown in yellow. Alkyl chains were replaced with methyl groups. H atoms are not shown for clarity.

To elucidate the nature of these emissions, we conducted temperature‐dependent measurements between 25°C (298 K) and −193°C (80 K). Under ambient conditions, the carbazole mixture **NBI2·MCz** and the pyrene mixture **NBI1·Pyr** displayed the longest lifetime components of ∼3 and ∼68 µs, respectively (Figure 4b; Figures S45 and S47). Both long‐lived components decreased at 80 K, consistent with TADF emission from the ^1^CT state (Figures S51,S52). At low temperature, an additional long‐lived phosphorescence band appeared, pointing toward RISC from the triplet manifold to the ^1^CT state in the **NBI1·Pyr** and **NBI2·MCz** mixtures. We substantiate this assignment via temperature‐dependent measurements in which both mixtures exhibit weak vibronic features, indicative of locally excited (LE) character. The experimentally estimated Δ*E*_ST_ values are 60 and 80 meV for **NBI1·Pyr** and **NBI2·MCz**, respectively. In order to show whether the triplet state responsible for RISC originates from the host or the guest, we experimentally determined ^3^LE states of the individual host and guest molecules (Figure 5a,b). We found that the ^3^LE states of the isolated guest molecules lie at higher energies than the corresponding ^1^CT states of the mixtures, indicating that the ^3^LE state of the NBI hosts is responsible for the RISC process. Theoretical calculations support our experimental observation and show that for both **NBI1·Pyr** and **NBI2·MCz**, the lowest‐energy singlet states are CT in nature, whereas the triplet states have predominantly LE character and localized on the **NBI1** and **NBI2** hosts (Figure 5c). The theoretically calculated Δ*E*_ST_ values of 10 and 30 meV are in good agreement with the experimentally determined values (Figure 5c). It is noteworthy that although the lowest triplet excited state (T_1_) of the NBI host is primarily ^3^LE in nature, it also contains a non‐negligible CT contribution. This mixed character is consistent with our experimental observations: a broad triplet emission with weak vibronic structure at very low temperatures, indicating a triplet state dominated by LE character with a partial CT contribution (Figure 5c).

The two Pt(II)‐complex mixtures, **NBI1·Pt1** and **NBI2·Pt2**, exhibited short‐lived PF in the nanosecond regime alongside a long‐lived microsecond component (Figures S46 and S48). Cryogenic measurements revealed that one long‐lived component decreases with decreasing temperature, while another increases, suggesting the coexistence of both TADF and RTP processes (Figures S53 and S54). To further probe the origin of this dual emission, we collected ^3^LE phosphorescence spectra of **NBI1** and **NBI2**. Compared to the vibronically resolved low‐temperature phosphorescence spectra of the NBI hosts sensitized by ethyl iodide (**EtI**), which originate from locally excited ^3^LE states, the observed spectra are broader, featureless, and red‐shifted, consistent with CT‐derived triplet emission (Figure S55). We therefore attribute the long‐lived emission of **NBI1·Pt1** and **NBI2·Pt2** under ambient conditions to the coexistence of TADF and RTP from ^1^CT and ^3^CT states. Accordingly, the heavy‐atom effect (HAE) of platinum promotes pronounced spin–orbit coupling (SOC) in the CT mixtures containing Pt(II) complexes, which stimulates both efficient intersystem crossing (ISC) to the triplet manifold and the radiative decay to the ground state [29, 30]. The resulting increase in the radiative rate shortens the excited‐state lifetime as apparent for **NBI1·Pt1** and **NBI2·Pt2** with the shorter lifetimes of up to ∼3 µs. In contrast, purely organic triplet emitters typically display longer‐lived triplet states due to their intrinsically slow radiative decay and comparatively high singlet‐triplet energy gap (∆*E*_ST_). A donor‐acceptor architecture, as in this case, can enable changes in orbital angular momentum, thereby enhancing SOC and promoting ISC. In addition, LE and CT states can mix, which further increases the efficiency of ISC, resulting in the coexisting TADF and RTP emissions of the LC mixtures [52, 53].

### 2.5 CT‐Mediated Energy‐Transfer Dual Emission

We further advanced the concept of supramolecular emission activation within the LC platform by deliberately co‐assembling the two NBI hosts, **NBI1** and **NBI2**, with the embedded guest **Pyr** to form a unified mesophase framework that simultaneously supports green and red emission, aiming for true dual emission combining prompt fluorescence and long‐lived delayed luminescence from TADF. With this architecture, we created an energy‐transfer cascade in which the energetically higher‐lying, green‐emitting **NBI2·Pyr** donor complex transfers its excitation energy into the CT band of the lower‐energy, red‐emitting **NBI1·Pyr** acceptor. In our approach, we exploit the tightly packed self‐assembly of columnar LCs, which serves as an ideal environment for efficient short‐range energy transfer processes [54]. To combine efficient energy transfer and color tuning, an excess of the green‐emitting complex is required, thereby taking advantage of the enantiotropic behavior of **NBI2**. Accordingly, we prepared mixtures with **NBI2**:**NBI1** ratios of 100:1, 100:5, 100:10, and 100:100 while maintaining a constant overall LC content of 5:1 **Pyr**.

POM investigations revealed focal‐conic textures for all mixtures (Figure S56) together with liquid appearance, suggesting that the columnar mesophase remains intact. DSC traces showed broad LC behavior for all compositions (Figures S57 and S58). Mixing the neat hosts in the absence of guests resulted in a modest increase in stability, reflected by a broader mesophase range and higher clearing temperatures. Upon addition of **Pyr**, host mixtures rich in **NBI2** became slightly destabilized, showing reduced clearing points, whereas increasing the **NBI1** fraction led to pronounced stabilization, with the mesophase broadened to nearly 100°C (Figure S58). This observation can be attributed to the shape complementarity between **NBI1** and **NBI2**, which enables efficient *π–π* stacking interaction. Similar to the host–guest mixtures discussed above, WAXS measurements showed that mixing the two hosts does not significantly alter the structural properties, with only minor changes observed in the lattice parameters (Figures S59–S62, Table S1).

Under UV illumination at the PL POM setup, the mixtures displayed a smooth color gradient: starting with the green emission of **NBI2·Pyr** (100:0), shifting through yellow (100:5) and orange (100:10), and culminating in the red emission of **NBI1·Pyr** (0:100) (Figure S56). Stability studies revealed that mixtures containing high proportions of **NBI2** remained stable over time, whereas the 1:1 mixture with **NBI1** exhibited pronounced structural degradation after several days, accompanied by a loss of the Col_h_ phase. Notably, the luminescence profile remained intact for all mixtures. (Figure S63 and Table S5).

**FIGURE 6:**
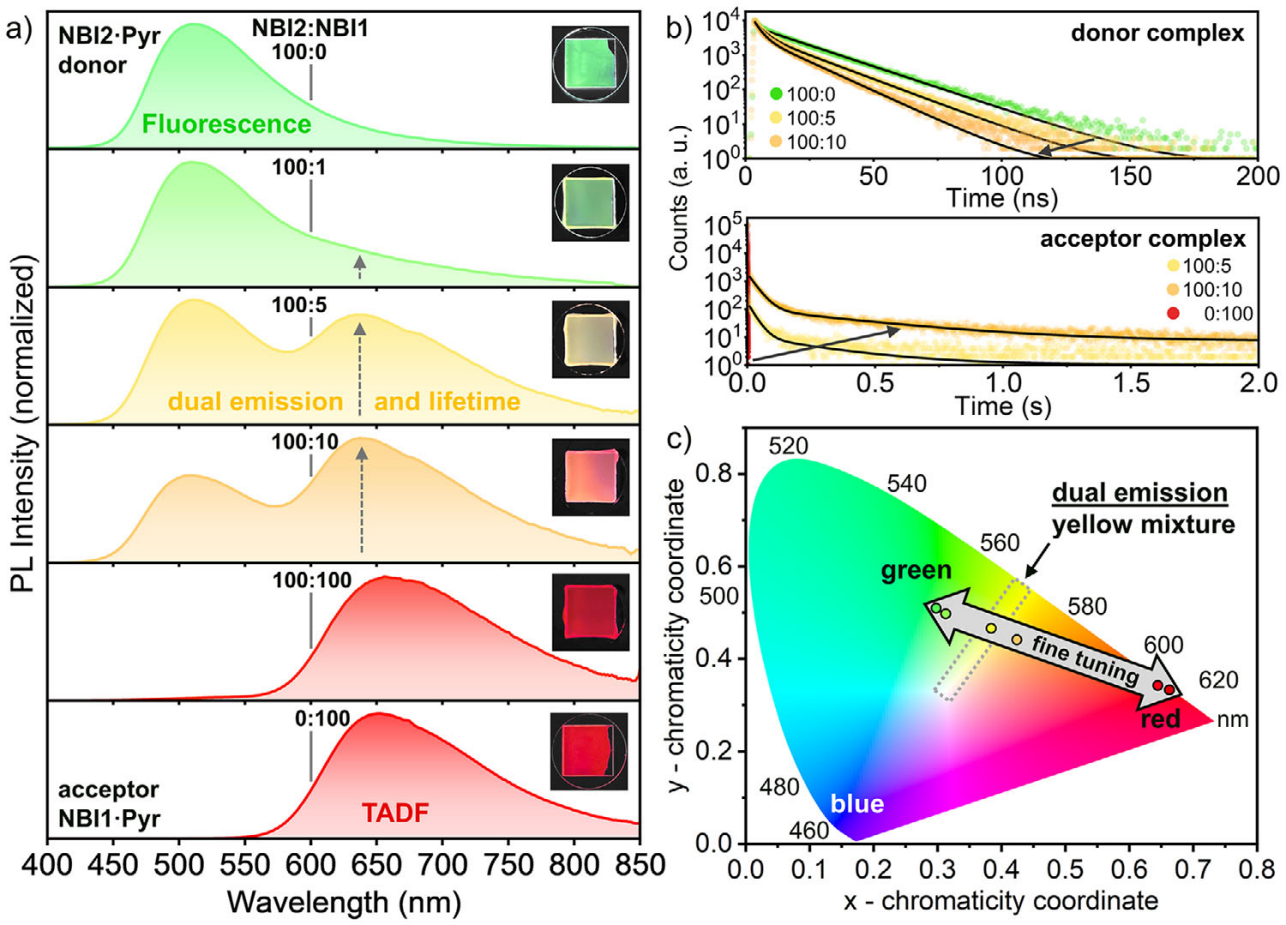
Energy‐transfer mixture of **(NBI2:NBI1)·Pyr** (NBIs:guest, 5:1) with given ratios of **NBI2**:**NBI1** showing dual emission and dual lifetime. (a) Normalized PL spectra of the mixtures in their color of emission with images of the substrates under UV light (*λ*_ex_ ≈ 400 nm, scale 10 × 10 mm^2^). (b) Representative PL lifetime decays (symbols) of the host mixtures (**NBI2**:**NBI1**) 100:5 (yellow) and 100:10 (orange) as well as **NBI2·Pyr** (green) and **NBI1·Pyr** (red) along with the respective best fit (black line) of the donor complex fluorescence at the first peak (*λ*_em_ = 510 nm) and the delayed luminescence of the acceptor complex at the emerging second peak (*λ*_em_ = 640 nm). (c) CIE diagram showing the possible range of color fine‐tuning visible to the human eye from **NBI2·Pyr** green to **NBI1·Pyr** red, with the position of the dual emission on the gray arrow.

For the following PL measurements, all host mixtures were excited at 430 nm, corresponding to the low energy CT band of the donor complex **NBI2·Pyr**. With increasing **NBI1** content in the host mixture from 100:1 to 100:100 (**NBI2**:**NBI1**) ratio, alongside the ^1^CT fluorescence of **NBI2·Pyr** with a maximum at ∼510 nm, a second emission band gradually emerged with a maximum at ∼640 nm, reflecting the growing contribution of the **NBI1** TADF to the overall luminescence (Figure 6a). Time‐resolved measurements at the two different PL maxima, i.e., 510 and 640 nm confirmed the prompt fluorescence at the first peak (**NBI2·Pyr**) and long‐lived luminescence at the emerging second peak (**NBI1·Pyr**, Figure 6b; Figures S64–S67). Additionally, we found that increasing the amount of acceptor complex led to a decrease in the donor complex lifetime (Figure 6b). These observations are consistent with energy transfer processes and validate our strategy for achieving dual emission [48, 55]. Moreover, the results show that by adjusting the host chromophore mixing ratio, it is possible to tune the luminescence color of the LC system smoothly from green to red (Figure 6c).

This energy‐transfer approach to utilize CT‐mediated emission in the LC phase enables continuous fine‐tuning of both emission color and excited‐state lifetime. By adjusting the mixing ratio, smooth color gradients from green through yellow, orange to deep red can be achieved, together with combined PF and TADF components. Such dual emission should be particularly attractive for anti‐counterfeiting labels, because both the complex spectral signature as well as the multi‐stage decay dynamics are difficult to replicate. With our strategy, we were able to cover two‐thirds of the RGB colors, and adding a blue component could produce white light in the future. The discovery of this exceptional LC mixing system can be further modulated by the usage of different chromophore compounds, a variety of guests or different processing conditions thereby enabling multifaceted applications.

### 2.6 Application Prospects

**FIGURE 7:**
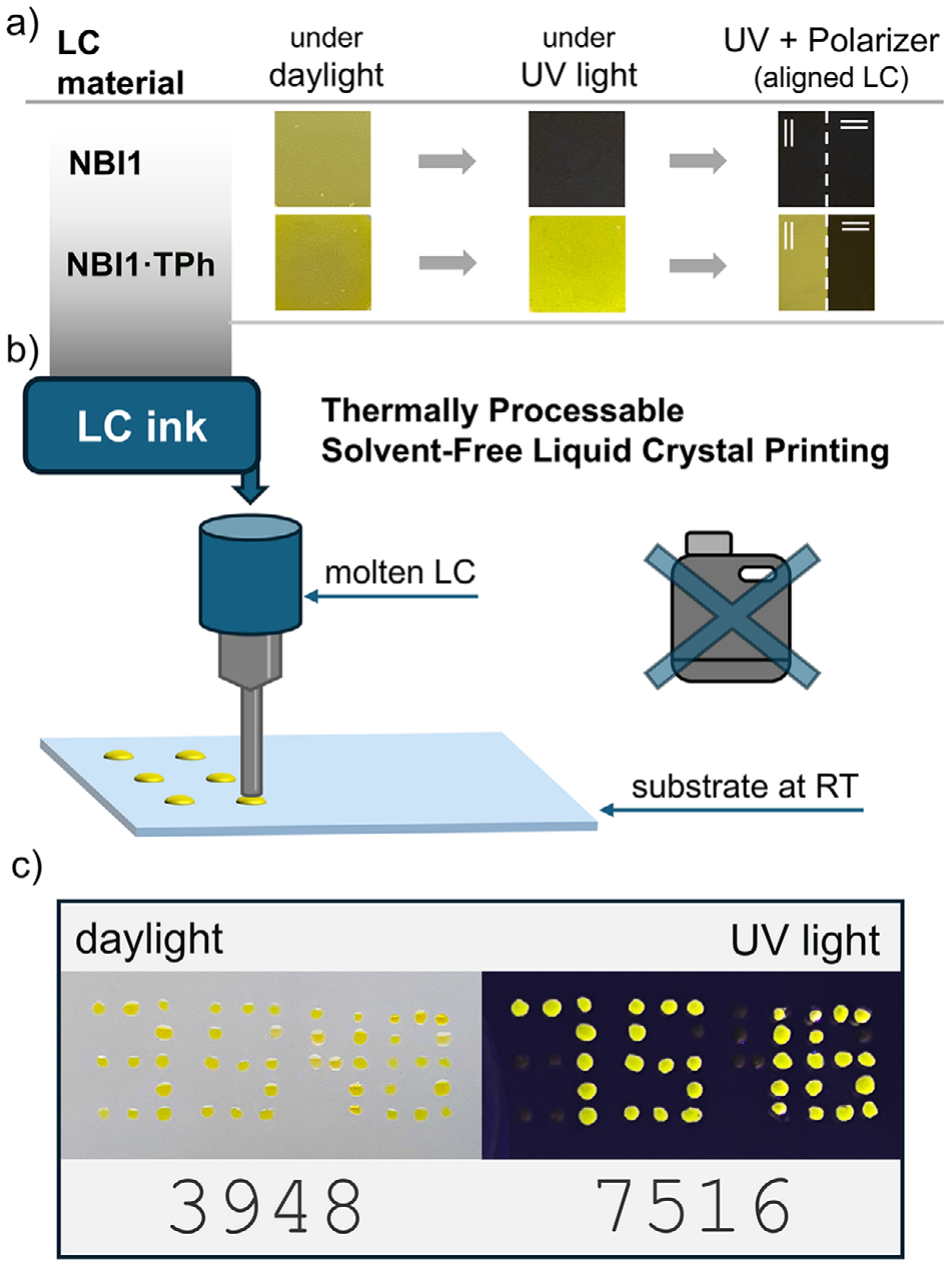
Application prospects of the LC mixtures. (a) Intrinsic properties of the aligned layers of LC that can be utilized as security features, showing neat LC host **NBI1** and the mixture **NBI1·TPh** (5:1) under white and UV light (with and without polarizer) illumination. (b) Schematic presentation of solvent‐free processing as LC ink using heat. (c) Encrypted numerical codes for anticounterfeiting purpose. 3948 is visible under daylight, while 7516 is hidden and only visible under UV light (spot diameter approximately 1 mm).

With a view toward potential applications, we utilized the intrinsic properties of the discotic LC for processing while simultaneously demonstrating an anticounterfeiting concept (Figure 7). Both the neat LC host **NBI1** and its mixture **NBI1·TPh** (5:1) exhibit a similar yellow appearance under daylight, rendering them visually indistinguishable (Figure 7a). Under UV illumination, however, the materials can be clearly differentiated. The LC host **NBI1** is non‐emissive, whereas the mixture **NBI1·TPh** shows yellow exciplex emission. Furthermore, the intrinsic alignment properties of the LC mixture provide an additional level of functionality. Upon shear processing, the mixture adopts a preferential molecular orientation, resulting in linearly polarized emissions that can be selectively filtered using a polarizer (Figures S28–S33).

The use of thermotropic LC is particularly attractive for future applications, as it enables a sustainable and environmentally friendly processing route that requires no additional solvent for mixing and processing. The LC mixtures can be prepared in a solvent‐free manner through simple mixing and melting, with the LC itself acting as the processing medium. Furthermore, these mixtures can be applied via a thermal printing process, followed by an annealing step that enables the formation of well‐defined, dot‐like features (Figure 7b,c).

We therefore melted the LC in a canula and applied it onto a glass substrate at room temperature. The LC ink attached to the surface, and upon removal of the cannula, an imprint of the applied geometry remained. This imprint could be eliminated by thermal annealing to the melting temperature after all spots had been deposited. Using this approach, we were able to produce spots with diameters of approximately 1 mm. As a result, we encrypted numerical codes in an anticounterfeiting concept, one that is visible under daylight (3948), while a second, hidden color code becomes visible only under UV light (7516) (Figure 7c). Further improvements in processing could incorporate linearly polarized light and employ different mixtures for yellow emission appearance (energy‐transfer mixtures for dual emission), enabling the fabrication of LC‐based security labels that are difficult to reproduce yet straightforward to process.

## 3 Conclusion

In this article, we demonstrated that liquid crystals (LCs) can serve as versatile emissive supramolecular hosts whose emission wavelengths and lifetimes can be tuned by the addition of guests. By designing electron‐deficient naphthalene bisimide (NBI) mesogens that form columnar hexagonal LC phases stabilized through nanosegregation, we created supramolecular donor–acceptor environments capable of activating and tuning photoluminescence through charge‐transfer interactions. Incorporation of polycyclic aromatic hydrocarbons, a carbazole derivative, and Pt(II) complexes afforded a broad spectral response spanning from the green to the near‐infrared spectral region. With regard to security applications, it is important that the investigated LC host–guest systems not only cover a wide range of the visible spectrum but also decay times from the lower nanosecond up to the high‐microsecond regime.

Thus, unlike conventional doped LC systems in which luminescence arises from fluorescent guest chromophores emitting in the narrow decay regime of a few nanoseconds, our host–guest strategy enables large variations in the decay time by involvement of emitting triplet states originating from CT interactions. We further showed that co‐assembly of two complementary NBI hosts with just one guest creates an energy‐transfer cascade that affords dual emission with both emission color and excited‐state lifetimes being tunable by simply adjusting the ratio of the LC host chromophores. Our study on luminescent LC mixtures highlights the potential of supramolecular design to unlock new emissive states and lifetime control within a processable LC platform. Together, the demonstrated tunability, combined with the intrinsic advantages of LC processability, our research provides a promising route toward advanced applications in optical encoding, multi‐mode authentication, and security labeling technologies.

## Funding

Deutsche Forschungsgemeinschaft (DFG, German Research Foundation) in the framework of IRTG 2991 “Photoluminescence in supramolecular matrices” (Project No. 517122340).

## Conflicts of Interest

The authors declare no conflicts of interest.

## Supporting information




**Supporting File**: adma72579‐sup‐0001‐SuppMat.pdf.

## Data Availability

The data that support the findings of this study are openly available in Zenodo at https://doi.org/10.5281/zenodo.17234268, reference number 17234268.
