# Supplementary material for: Guest‐Induced Activation of Multicolor Photoluminescence in Naphthalene Bisimide Liquid Crystals
Source: Adv Mater. 2026 Feb 25;38(17):e20184. doi: 10.1002/adma.202520184 (PMC13003916; doi:10.1002/adma.202520184)
Supplement: Supplementary file 1 — Supporting File: adma72579‐sup‐0001‐SuppMat.pdf. [file ADMA-38-e20184-s001.pdf]

Supporting Information

Guest-induced Activation of Multicolor Photoluminescence in Naphthalene Bisimide Liquid Crystals

*Johannes Nowarra, Swadhin Garain, Matthias Stolte, Frank Würthner**

J. Nowarra, Dr. S. Garain, Dr. M. Stolte, Prof. Dr. F. Würthner

Institut für Organische Chemie and Center for Nanosystems Chemistry (CNC), Universität Würzburg, Am Hubland, 97074 Würzburg, Germany.

E-mail: frank.wuerthner@uni-wuerzburg.de

Table of Contents

1. Materials and Methods S2
2. Synthesis S5
3. Experimental Data S13
4. NMR Spectra S73
5. Mass Spectrometry S78
6. Supporting References S81

1. Materials and Methods

All chemicals, reagents and solvents were purchased from commercial suppliers and used after appropriate purification if not stated otherwise. *N*-methylcarbazole was synthesized in our laboratory to avoid the carbazole isomers.[S1] Dichloromethane (CH_2Cl_2) was distilled prior to use. Column chromatography was performed with 60 M silica gel, particle size 0.04-0.063 mm (*Merck KGaA*). Normal phase high-performance liquid chromatography (HPLC) was performed on a recycling semipreparative HPLC system (*JASCO Inc.*) equipped with a VP 250/21 NUCLEOSIL 100-7 column (*Macherey-Nagel GmbH*).

UV/Vis spectroscopy was carried out with a V770 spectrophotometer (*JASCO Inc.*) equipped with a Peltier temperature control unit. Measurements were performed in 10 mm SUPRASIL® quartz cuvettes (*Hellma GmbH)* and spectroscopic grade CH_2Cl_2.

Photoluminescence spectroscopy in solution was carried out on a FLS980-D2D2-ST spectrometer (*Edinburgh Instruments Ltd.*) with temperature-controlled sample holder at 20 °C (293 K). Spectroscopy of thin films was carried out with the same spectrometer on a F-J03 front-face sample holder at 22.5° and mirror system at ambient conditions. Temperature-dependent photoluminescence spectra from −193 °C (80 K) to 25 °C (298 K) were recorded using a nitrogen flow cryostat connected to a Mercury ITC temperature controller (both *Oxford Instruments plc*). Spectra were corrected against lamp intensity and photomultiplier sensitivity. Time-resolved measurements were performed either with EPL series picosecond pulsed diode lasers (λ_{ex} = 403.8 nm and 479.7 nm) with a time-correlated single photon counting (TCSPC) detection unit, a µF2 pulsed Xenon microsecond flashlamp with variable wavelength or an AGILE® wavelength-tunable supercontinuum laser, both detected with a standard UV/Vis photomultiplier tube (PMT) photon counting detector (all *Edinburgh Instruments Ltd.*).

Absolute photoluminescence luminescence quantum yields (Φ_{PL}) were determined with a C9920-02 Absolute PL Quantum Yield Measurement System (*Hamamatsu Photonics KK*) including a 150 W CW Xenon lamp, manually adjustable monochromator, a photonic multichannel analyzer PMA-12 and an Ulbricht integrations sphere with a diameter of 8.4 cm and spectralon®-coating. The quantum yield was measured as an average of three measurements performed at a minimum of three different excitation wavelengths.

Nuclear magnetic resonance (NMR) spectroscopy was performed on an Avance III HD 400 MHz spectrometer (*Bruker Daltonic GmbH*). Chemical shifts (δ) are listed in parts per

million (ppm) relative to the residual solvent signal. The multiplicities for proton signals are abbreviated as s, d and t for singlet, doublet and triplet, respectively.

High-resolution mass spectrometry (HRMS) was measured either with electrospray ionization – time of flight (ESI-TOF) on a microTOF-Q III mass spectrometer (*Bruker Daltonic GmbH*) or matrix-assisted laser desorption/ionization – time of flight (MALDI-TOF) on a ultrafleXtreme mass spectrometer (*Bruker Daltonic GmbH*). Trans-2-[3-(4-*tert*-butylphenyl)-2-methyl-2-propenylidene]-malononitrile (DCTB) was used as the matrix.

Differential Scanning Calorimetry (DSC) was performed on a DSC 8000 with the cooling accessory Intracooler 2 (both *PerkinElmer Inc.*) in aluminum pans and covers with a temperature rate of 10 K min^{-1}. For better visibility, some of the baselines were corrected in Origin. The evaluation was performed prior to baseline correction using the Pyris software (*PerkinElmer Inc.*).

Melting points (for solids and crystals) and **clearing points** (for liquid crystals) were determined either by POM (see next section) or DSC (see section above) with a temperature rate of 10 K min^{-1}.

Polarized optical microscopy (POM) with photoluminescence (PL) spectroscopy of liquid crystal textures was performed on an Axio Imager 2 polarization microscope (*Carl Zeiss AG*). The microscope is equipped with a LTS420 stage and T95-HS+LNP95 temperature control system (*Linkam Scientific Instruments Ltd.*). PL spectroscopy was performed with the microscope and a HXP R 120 W/45 C UV mercury short-arc lamp (*OSRAM*) as excitation source, a filter cube system 424931 *(Carl Zeiss AG)* for different excitation and detection ranges (see Figure S11) and a Maya 2000Pro® spectrometer (*Ocean Insights - formerly Ocean Optics Inc.*) with a back-thinned CCD detector (*Hamamatsu Photonics KK*). Spectra are not corrected against lamp intensity and sensitivity of the detector.

Single crystal X-ray diffraction measurements were performed on a D8 Quest diffractometer with a 2D Photon II CPAD detector (both *Bruker AXS GmbH*) at 100 K. The diffraction data was processed with APEX3 and APEX4 software and the structure was solved and subsequently further processed using Fourier techniques with SHELXT software.[S2] Crystallographic data is deposited at the Cambridge Crystallographic Data Centre (CCDC) as supplementary publication number 2494985 available via www.ccdc.cam.ac.uk/data_request/cif.

Wide angle X-ray scattering (WAXS) measurements were performed either on a Nanostar X-ray diffractometer (*Bruker AXS GmbH*) with a Microfocus IμSCu copper X-ray source (*Incoatec GmbH*) and a Vantec-2000 detector (*Bruker AXS GmbH*) in 21 cm distance and 14° angle of detection (WAXS pattern in reddish) or a D8 Quest diffractometer with a 2D Photon II 14 CPAD detector (both *Bruker AXS GmbH*) in 10 cm distance and 0° angel of detection (WAXS pattern in white/black). WAXS data was processed and evaluated with the X-ray analysis program Datasqueeze (version 3.0.23) with calibration to AgBh.[S3,S4] Peak position and FWHM were determined by Datasqueeze fitting with Voigt and Gaussian functions and a polynomial fitting for background correction was used as baseline.

Glovebox UNILAB pro (*M. Braun Inertgas-Systeme GmbH*) with antechamber total vacuum 5×10^{-2} mbar and inert gas nitrogen was used for reactions and sample preparation with monitored operating values for H_2O < 0.5 ppm and O_2 < 0.5 ppm.

Ball Mill MM 400 (*Retsch GmbH*) with sealable stainless steel grinding jars and stainless steel grinding balls was used for reaction.

Computational methods were used to calculate the frontier molecular orbitals of the molecules and excited state energies. Calculations for HOMO and LUMO values were carried out with Gaussian 16. Geometry optimizations were carried out at the B3LYP-D3BJ/def2-TZVP level of theory.[S5,S6] Calculations for excited state energies were carried out with ORCA 6 at the B3LYP-D3BJ/def2-TZVP(-f) level of theory for geometry optimization and TD-B3LYP/def2-TZVP(-f) for vertically excited state energies and difference densities. Images were rendered with UCSF Chimera. Alkyl chains were replaced with methyl groups.

2. Synthesis

The compounds utilized in this article were synthesized according to the routes depicted in the following schemes.

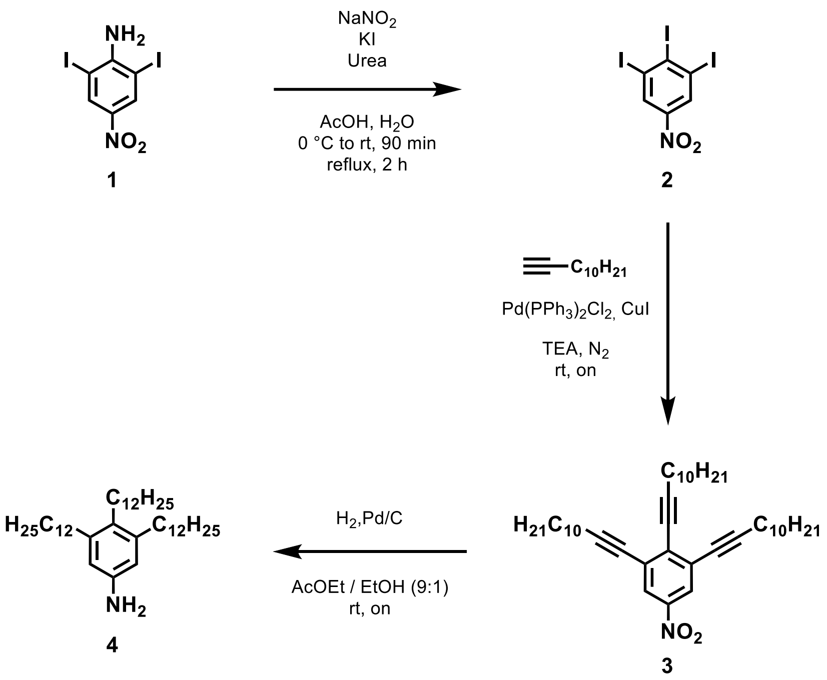

Scheme S1. Synthesis route of the mesogenic unit tridodecyl aniline **4**.[S7]

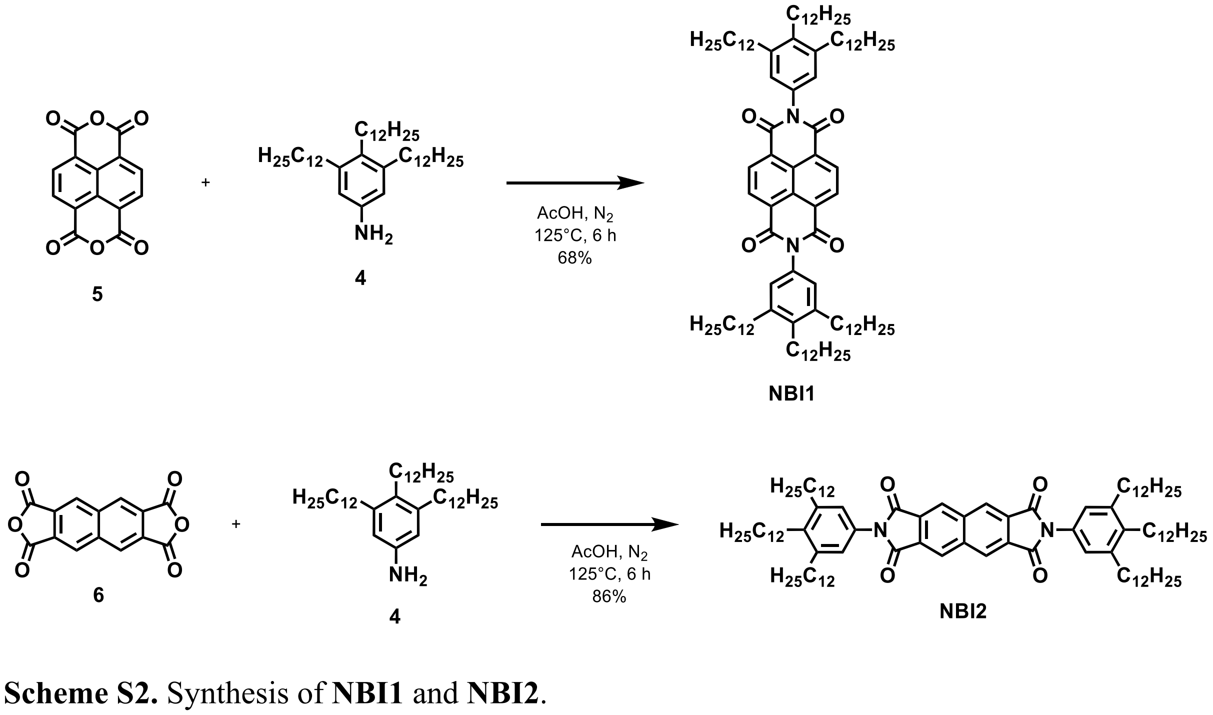

Scheme S2. Synthesis of **NBI1** and **NBI2**.

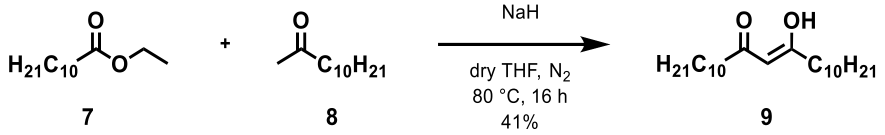

Scheme S3. Synthesis of precursors **9** for the Pt(II) complex synthesis.

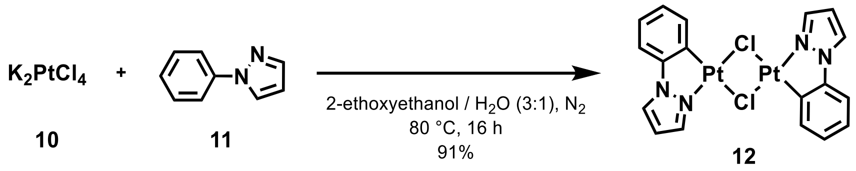

Scheme S4. Synthesis of precursors **12** for the Pt(II) complex synthesis.[S8]

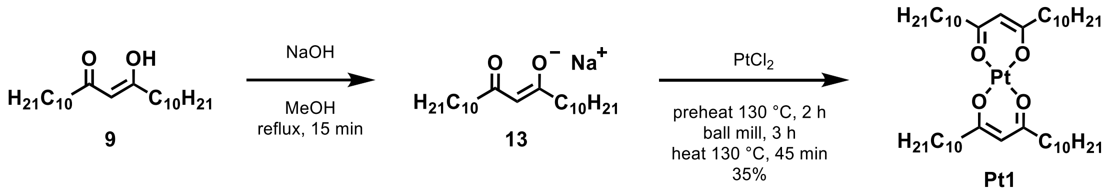

Scheme S5. Synthesis of the Pt(II) complex **Pt1**.

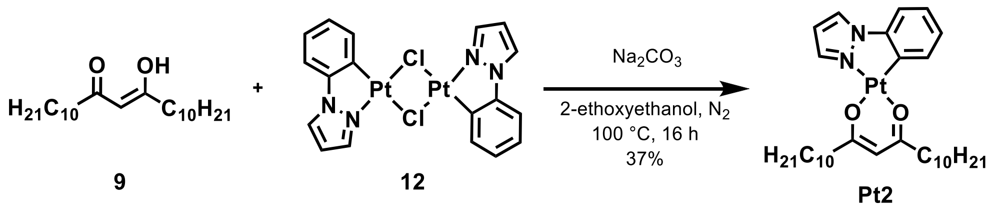

Scheme S6. Synthesis of the Pt(II) complex **Pt2**.

***N,N′*-bis(3,4,5-tridodecylbenzyl)-naphthalene-1,8:4,5-bis(dicarboximide) (NBI1)**

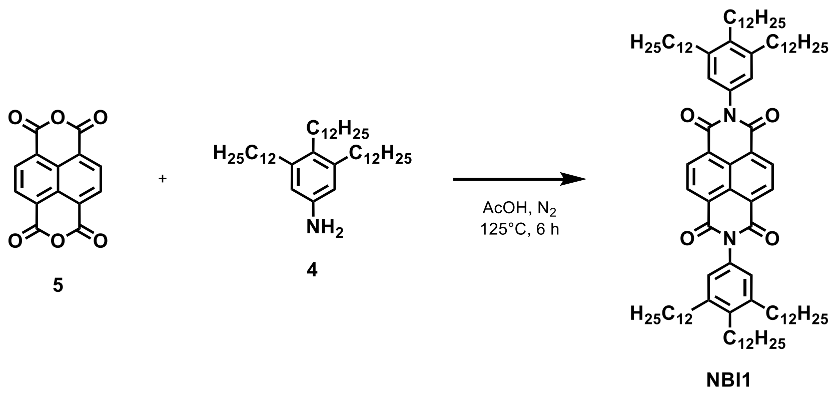

Naphthalene-1,8:4,5-tetracarboxylic dianhydride (**5**) (470 mg, 1.75 mmol, 1.0 eq.) and 3,4,5-tri(dodecyl)aniline (**4**) (2.30 g, 3.68 mmol, 2.2 eq.) were placed in a Schlenk-flask and set under nitrogen. Glacial acetic acid (100 mL) was added, and the reaction mixture was stirred for 6 h at reflux temperature. Thereafter the mixture was cooled to room temperature and extracted with dichloromethane (3 × 150 mL) and water (3 × 150 mL). The combined organic layer was dried over Na_2SO_4 and concentrated under reduced pressure. The crude product was purified by silica column chromatography eluted in dichloromethane/cyclohexane (*v*/*v*, 1:1) and recrystallized in methanol/chloroform (*v*/*v*, 2:3).

Yield: 1,7 g (1,19 mmol, 68%) of a faint yellow powder.

^{1}H NMR (400 MHz, $CDCl_3$, 295 K): δ 8.83 (s, 4H), 6.96 (s, 4H), 2.76–2.55 (m, 12H), 1.70–1.15 (m, 120H), 0.97–0.79 (m, 18H) ppm.

^{13}C NMR (101 MHz, $CDCl_3$): δ 163.3, 142.5, 139.9, 131.8, 131.4, 127.24, 127.22, 126.2, 33.3, 32.2, 32.1, 32.07, 31.9, 31.7, 31.4, 30.9, 30.6, 30.1, 29.9, 29.88, 29.85, 29.81, 29.76, 29.7, 29.56, 29.55, 29.5, 29.0, 23.0, 22.9, 22.8, 14.29, 14.27, 14.1 ppm.

HRMS (MALDI-TOF, pos. mode, $CHCl_3$/DCTB, 1:3): (*m*/*z*), calcd. for $C_{98}H_{159}N_2O_4^+$ $[M+H]^+$: 1428.2294, found: 1428.2252.

Clearing point: 46.2 °C (DSC).

UV/Vis: λ_{max} = 380 nm, ε = 25 000 $M^{-1}cm^{-1}$ (CH_2Cl_2).

N,N'-bis(3,4,5-tridodecylbenzyl)-naphthalene-2,3:6,7-bis(dicarboximide) (**NBI2**)

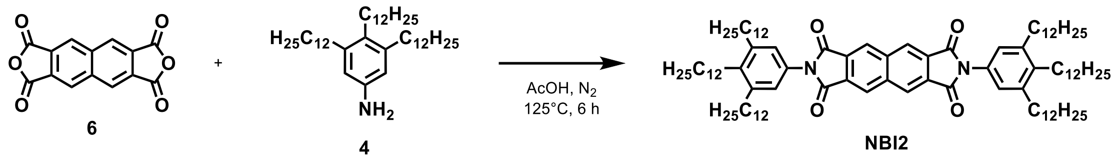

Naphthalene-2,3:6,7-tetracarboxylic dianhydride (**5**) (150 mg, 0.56 mmol, 1.0 eq.) and 3,4,5-tri(dodecyl)aniline (**4**) (736 mg, 1.23 mmol, 2.2 eq.) were placed in a Schlenk-flask and set under nitrogen. Glacial acetic acid (30 mL) was added, and the reaction mixture was stirred for 6 h at reflux temperature. Thereafter the mixture was cooled to room temperature and extracted with dichloromethane (3 × 50 mL) and water (3 × 50 mL). The combined organic layer was dried over Na_2SO_4 and concentrated under reduced pressure. The crude product was purified by silica column chromatography eluted in dichloromethane /cyclohexane (*v*/*v*, 1:2).

Yield: 916 mg (641 µmol, 86%) of a brown viscous solid.

^{1}H NMR (400 MHz, $CDCl_3$, 295 K): δ 8.66 (s, 4H), 7.08 (s, 4H), 2.76–2.55 (m, 12H), 1.70–1.15 (m, 120H), 0.97–0.79 (m, 18H) ppm.

^{13}C NMR (101 MHz, $CDCl_3$): δ 166.5, 142.3, 139.7, 138.6, 130.7, 128.7, 126.6, 124.8, 33.3, 32.1, 32.08, 31.5, 31.0, 30.5, 30.1, 29.89, 29.87, 29.85, 29.82, 29.78, 29.7, 29.54, 29.52, 28.9, 22.86, 22.85, 14.3 ppm.

HRMS (MALDI-TOF, pos. mode, $CHCl_3$/DCTB, 1:3): (*m*/*z*), calcd. for $C_{98}H_{158}N_2NaO_4^+$ $[M+Na]^+$: 1450.2114, found: 1450.2088.

Clearing point: 77.7 °C (DSC).

UV/Vis: λ_{max} = 376 nm, ε = 6 000 $M^{-1}cm^{-1}$ (CH_2Cl_2).

Dichloro-bridged Pt(II) 1-phenylpyrazol dimer [Pt(µ-Cl)(ppz)]$_2$ (12)

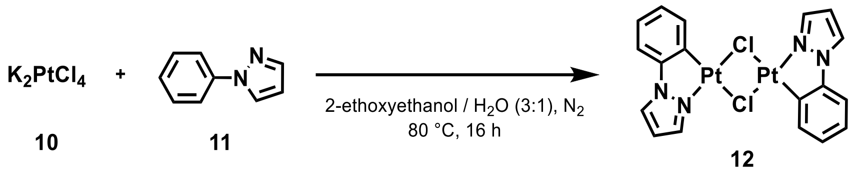

This compound was synthesized by an adjusted procedure, originally reported in literature.[S8] K_2PtCl_4 (**10**) (300 mg, 722 µmol, 1.0 eq.) and 2-phenylpyrazole (**11**) (208 mg, 1.45 mmol, 2.0 eq.) were added to a mixture of 2-ethoxyethanol (4.5 mL) and deionized water (1.5 mL) (*v*/*v*, 3/1) and set under nitrogen. The reaction mixture was stirred at 80 °C for 16 h. After the reaction mixture cooled down to room temperature, water (15 mL) was added, and the precipitate was filtered and washed with water (3 × 30 mL). The crude product was then dried under reduced pressure and used in the next step without further purification and characterization.

Yield: 491 mg (91%) of a gray solid.

13-hydroxy-12-tricosen-11-one (9)

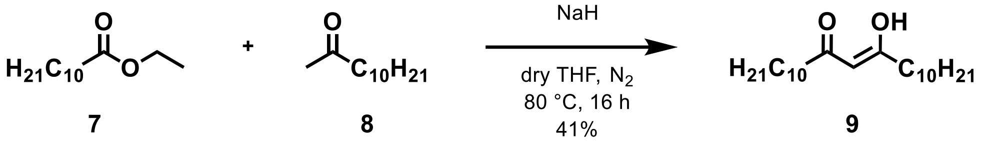

NaH (60% dispersion in mineral oil, 3.36 g, 140 mmol, 5.0 eq.) was added to a round bottom flask under nitrogen atmosphere and cooled in an ice-bath. Then a solution of ethyl undecanoate (**7**) (6.00 g, 28.0 mmol, 1.0 eq.) and 2-dodecanone (**8**) (5.68 g, 30.8 mmol, 1.1 eq.) in dry THF (30 mL) was slowly added under nitrogen counterflow. The reaction mixture was heated to 80 °C and stirred for 16 h. After cooling down to room temperature, the mixture was poured on ice and left for 2 h. HCl was added until neutral and the organic phase was extracted with dichloromethane (3 × 30 mL) and water (2 × 30 mL). The combined organic phase was dried over Na_2SO_4 and concentrated under reduced pressure. The crude product was recrystallized from refluxing methanol (300 mL).

Yield: 4.06 g (11.5 mmol, 41%) of white crystals.

^{1}H NMR (400 MHz, $CDCl_3$, 295 K): (0.15H cannot be unambiguously assigned) δ 15.56 (s, 0,75H), 5.47 (s, 0.85H), 3.54 (s, 0.25H), 2.49 (t, J = 7.5 Hz, 0.6H), 2.26 (t, J = 7.5 Hz, 3.4H), 1.64–1.56 (m, 4H), 1.34–1.22 (m, 28H), 0.88 (t, J = 7.0 Hz, 6H) ppm.

^{13}C NMR (101 MHz, $CDCl_3$): δ 204.7, 194.7, 99.2, 57.4, 43.9, 38.6, 32., 29.71, 29.70, 29.6, 29.6, 29.5, 29.45, 29.4, 29.2, 25.9, 23.5, 22.8, 14.3 ppm.

HRMS (ESI-TOF, pos. mode, $CHCl_3$/MeCN 1:1): (m/z), calcd. for $C_{23}H_{44}NaO_2^+$ $[M+Na]^+$: 375.3239, found: 375.3248.

Melting Point: 46 – 48 °C (from CH_2Cl_2).

Platinum(II) bis(tricosane-11,13-dione) [Pt(tcd)$_2$] (Pt1)

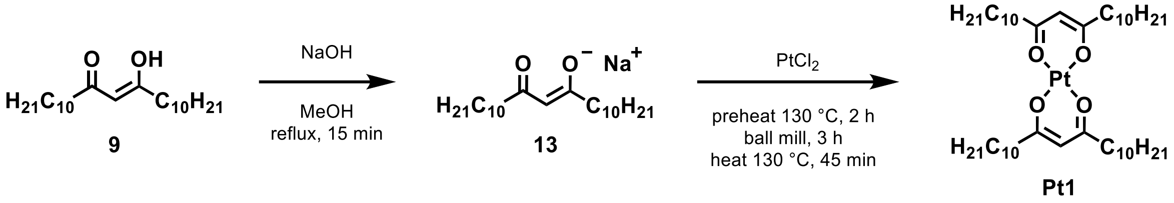

NaOH (226 mg, 5.68 mmol, 2.0 eq.) was dissolved in methanol (100 mL) and 13-hydroxy-12-tricosen-11-one (**9**) (1.00 g, 2.84 mmol, 1.0 eq.) was added. The reaction mixture was stirred at reflux for 15 min. The mixture was cooled down to −18 °C and the precipitate was filtered. The obtained solid was washed with cold (−18 °C from freezer) methanol (50 mL) and subsequently dried. The crude sodium enolate (**13**) (500 mg, 1.38 mmol, 50%) was obtained as a white powder and was used without further purification.

A grinding jar was filled with the sodium enolate (**13**) (500 mg, 1.38 mmol, 1.0 eq.) and $PtCl_2$ (141 mg, 550 µmol, 0.4 eq.) and sealed in a glovebox under nitrogen atmosphere. The reaction vessel was placed in an oven at 130 °C for 2 h, followed by ball mill activation at 30 Hz for 3 h. The activated mixture was then reacted in an oven at 130 °C for 45 min and cooled down to room temperature. The crude product was purified by silica column chromatography eluted in dichloromethane/cyclohexane (*v*/*v*, 1:1) and recrystallized from hexane.

Yield: 175 mg (247.01 µmol, 35 %) of faint yellow powder.

^{1}H NMR (400 MHz, $CDCl_3$, 295 K): δ 5.47 (s, 2H), 2.25–2.13 (m, 8H), 1.67–1.54 (m, 8H), 1.38–1.18 (m, 56H), 0.88 (t, J = 7.0 Hz, 12H) ppm.

^{13}C NMR (101 MHz, $CDCl_3$): δ 189.2, 101.3, 39.5, 32.1, 29.74, 29.65, 29.50, 29.48, 29.4, 26.96, 22.84, 14.28 ppm.

HRMS (MALDI-TOF, pos. mode, $CHCl_3$/DCTB, 1:3): (*m*/*z*), calcd. for $C_{46}H_{86}O_4NaPt^+$ $[M+Na]^+$: 920.6066, found: 920.6088.

Melting point: 85.2 °C (DSC).

UV/Vis: λ_{max} = 350 nm, ε = 4 400 $M^{-1}cm^{-1}$ (CH_2Cl_2).

Platinum(II) (1-phenylpyrazole)(tricosane-11,13-dione) [Pt(ppz)(tcd)] (Pt2)

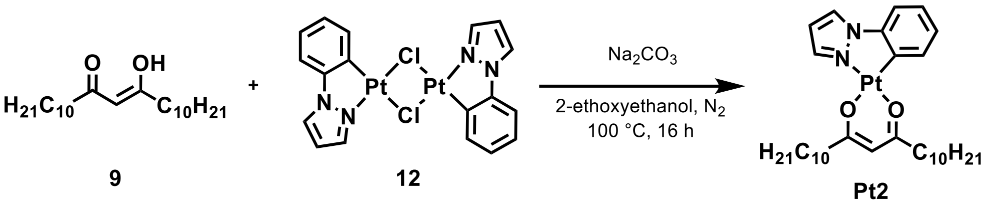

9 12 Pt2

13-hydroxy-12-tricosen-11-one (**9**) (354 mg, 1.00 mmol, 3.0 eq.), $[Pt(\mu\text{-}Cl)(ppz)]_2$ (**12**) (250 mg, 334 µmol, 1.0 eq.) and Na_2CO_3 (355 mg, 3.34 mmol, 10.00 eq.) were taken in a Schlenk-tube. Subsequently, 2-ethoxyethanole (4 mL) was added and the reaction was set under nitrogen atmosphere. The reaction mixture was stirred at 100 °C for 16 h. After cooling down to room temperature the precipitate was extracted with dichloromethane (3 × 20 mL) and water (5 × 20 mL). The combined organic phase was dried over Na_2SO_4 and concentrated under reduced pressure. The crude product was purified by silica column chromatography eluted in dichloromethane /hexane (*v*/*v*, 1:1) and recrystallized from hexane.

Yield: 170 mg (247 µmol, 37%) of faint green crystals.

^{1}H NMR (400 MHz, $CDCl_3$, 295 K): δ 7.92 (dd, J = 2.8, 0.6 Hz, 1H), 7.77 (dd, J = 2.2, 0.6 Hz, 1H), 7.66–7.46 (m, 1H), 7.18–7.03 (m,3H), 6.52 (t, J = 2.6 Hz, 1H), 5.46 (s, 1H), 2.30–2.18 (m, 4H), 1.77–1.63 (m, 4H), 1.42–1.19 (m, 26H), 0.88 (dd, J = 7.0, 6.4 Hz, 6H) ppm.

^{13}C NMR (101 MHz, $CDCl_3$): δ 188.9, 186.9, 144.6, 137.5, 131.9, 125.6, 125.2, 123.9, 122.8, 110.0, 106.3, 101.6, 41.2, 40.5, 32.1, 29.80, 29.78, 29.72, 29.65, 29.6, 29.51, 29.46, 26.7, 26.4, 22.9, 14.9 ppm.

HRMS (ESI-TOF, pos. mode, $CHCl_3$/MeCN 1:1): (*m*/*z*), calcd. for $C_{32}H_{50}N_2NaO_2Pt^+$ $[M+Na]^+$: 712.3412, found: 712.3416.

Melting point: 75.8 °C (from CH_2Cl_2).

UV/Vis: λ_{max} = 315 nm, ε = 11 000 $M^{-1}cm^{-1}$ (CH_2Cl_2).

3. Experimental Data

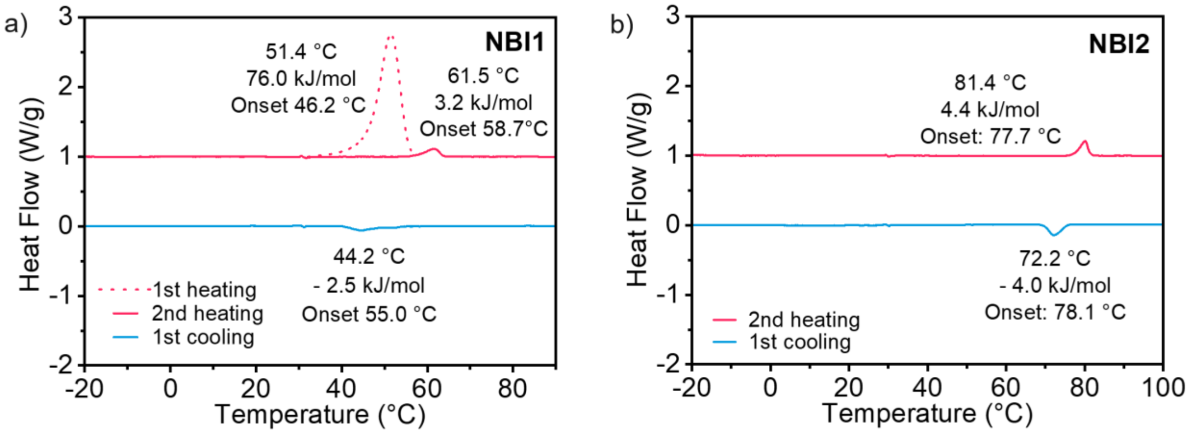

Figure S1. DSC traces of the NBI host molecules a) **NBI1** and b) **NBI2** from the second heating (red) and first cooling (blue) cycles with a rate of 10 K min^{-1}, endo up. For **NBI1** the first cooling (red dashed) is also shown.

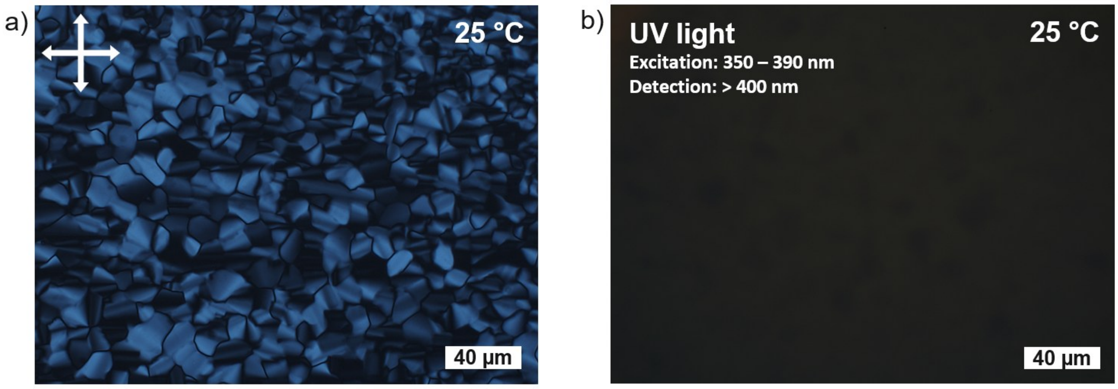

Figure S2. a) POM image of **NBI1** at 298 K recorded in transmission mode with crossed polarizers after heating to the isotropic liquid and b) PL-POM image recorded in reflection mode under UV light excitation showing negligible emission.

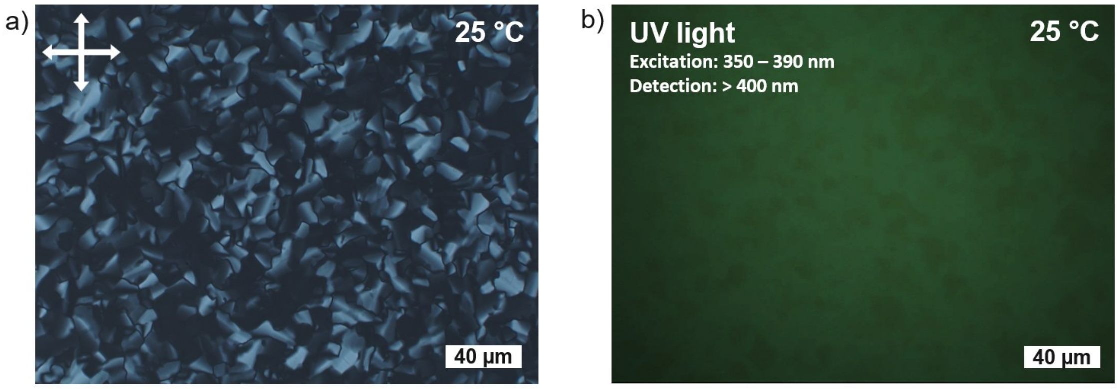

Figure S3. a) POM image of **NBI2** at 298 K recorded in transmission mode with crossed polarizers after heating to the isotropic liquid and b) PL-POM image recorded in reflection mode under UV light excitation showing weak emission.

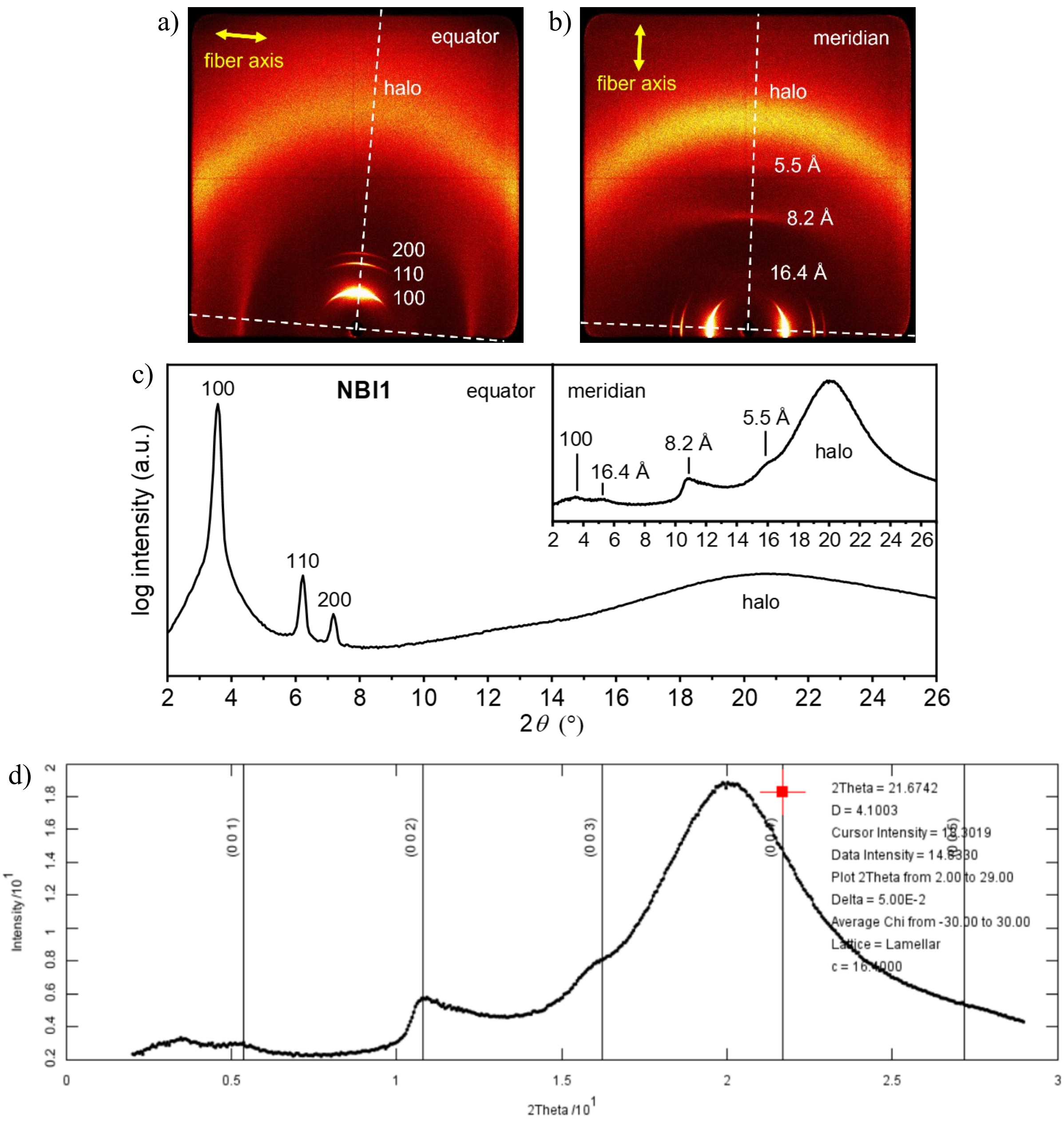

Figure S4. WAXS patterns of **NBI1** at 298 K of a) a lying fiber and b) a standing fiber. The position of the equator and meridian are indicated with white dashed lines. The direction of the fiber is indicated by yellow arrows. c) Integrated intensities along the equator and meridian (inlay) of the WAXS pattern. d) Integrated intensities along the meridian with lamellar order in the columns. The red cursor is located at 4.10 Å.

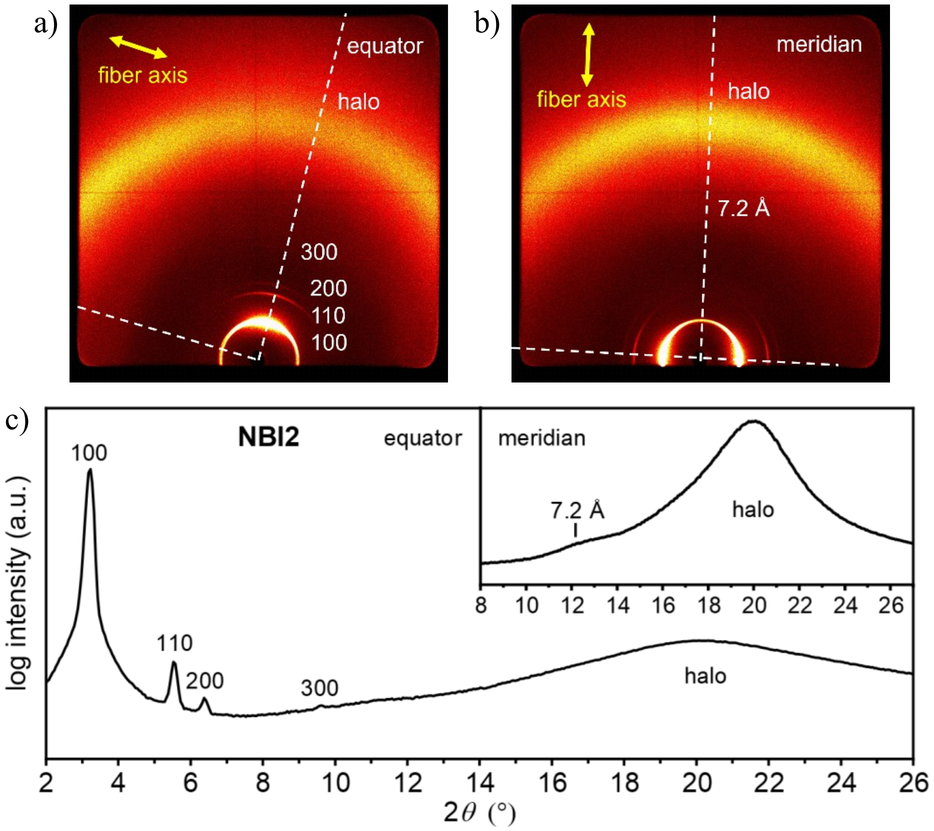

Figure S5. WAXS patterns of **NBI2** at 298 K of a) a lying fiber and b) a standing fiber. The position of the equator and meridian are indicated with white dashed lines. The direction of the fiber is indicated by yellow arrows. c) Integrated intensities along the equator and meridian (inlay) of the WAXS pattern.

a)

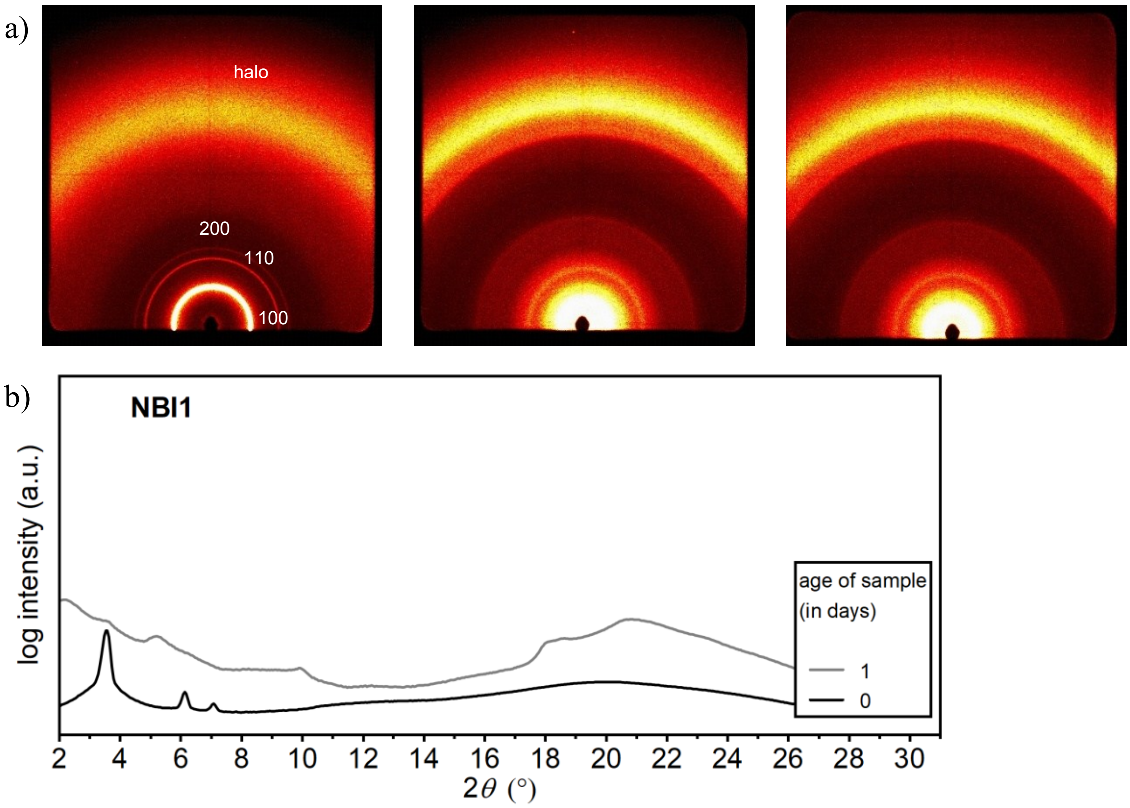

Figure S6. WAXS pattern of unaligned **NBI1** at 298 K a) after sample preparation (left), 1 day (middle) and 10 days (right) and b) integrated intensities of the WAXS pattern after sample preparation (black) and 1 day (gray).

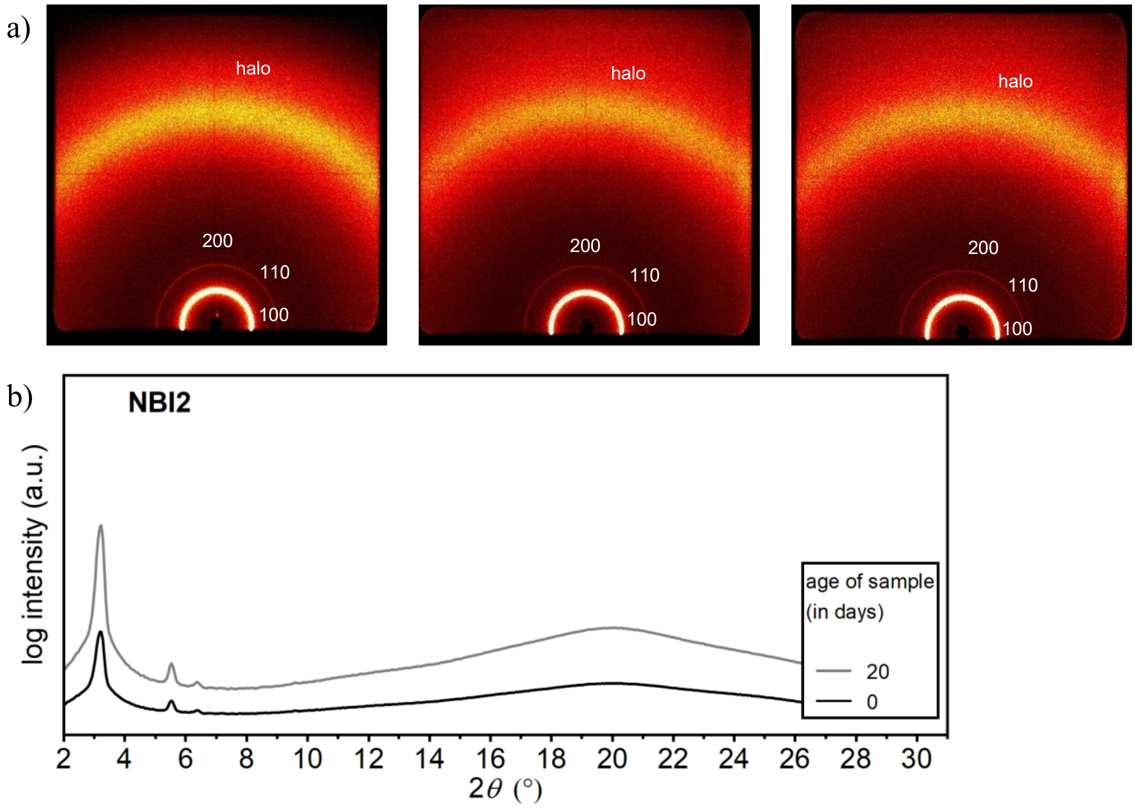

Figure S7. WAXS pattern of unaligned **NBI2** at 298 K a) after sample preparation (left), after 20 days (right) b) integrated intensities of the WAXS pattern after sample preparation (black) and 20 days (gray).

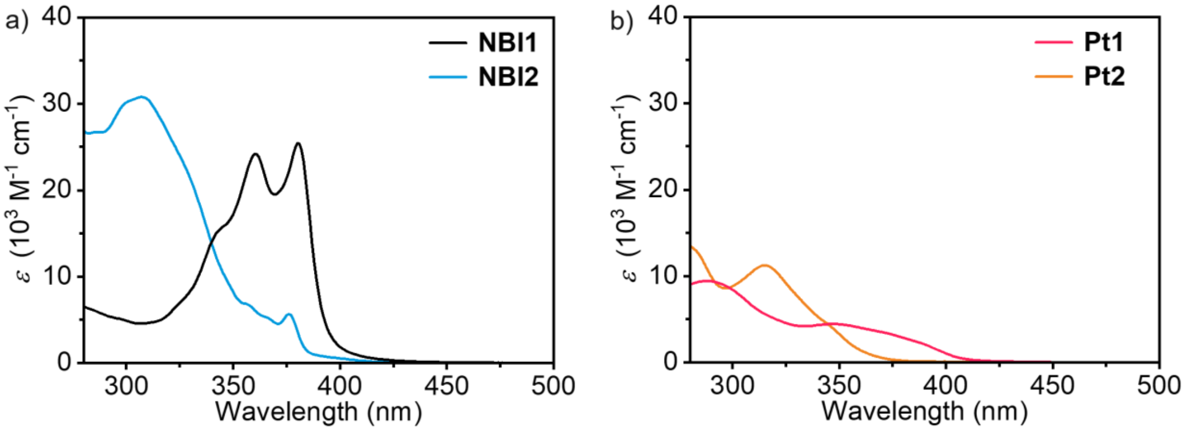

Figure S8. UV/Vis absorption spectra of a) **NBI1** (black) and **NBI2** (blue) and b) **Pt1** (red) and **Pt2** (orange) in $CHCl_3$ solution (10^{-5} M) at 298 K.

Table S1. Summary of the structural properties of **NBI1** and **NBI2** as well as mixtures with guest molecules **MCz**, **Pyr**, **TPh**, **Pt1** and **Pt2**.

Mixture (ratio)	Phase (T [°C])	a_{obs} [Å]	ξ [Å]	d_{obs} [Å]	*hk*	Transition temp. [°C]	Enthalpy [kJ/mol]
**NBI1**	Col_h (25)	28.6	504	24.80	10	H: −20 Col_h 59 Iso	3.2
				14.21	11	C: Iso 55 Col_h −20	−2.5
				12.31	20		
**NBI1·Pyr** (5:1)	Col_h (25)	28.3	443	24.54	10	H: −20 Col_h 71 Iso	3.7
				14.14	11	C: Iso 64 Col_h −20	−3.3
				12.25	20		
**NBI1·Pt1** (5:1)	Col_h (25)	28.9	496	25.01	10	H: −20 Col_h 61 Iso	4.9
				14.35	11	C: Iso 54 Col_h −20	−5.2
				12.43	20		
**NBI1·TPh** (5:1)	Col_h (25)	28.8	385	24.96	10	H: −20 Col_h 66 Iso	3.8
				14.39	11	C: Iso 66 Col_h −20	−3.5
				12.47	20		
**NBI2**	Col_h (25)	31.8	453	27.57	10	H: −20 Col_h 78 Iso	4.4
				15.95	11	C: Iso 78 Col_h −20	−4.0
				13.83	20		
				8.23	30		
**NBI2·Pyr** (5:1)	Col_h (25)	32.6	389	28.23	10	H: −20 Col_h 71 Iso	4.1
				16.30	11	C: Iso 69 Col_h −20	−4.1
**NBI2·MCz** (5:1)	Col_h (25)	32.6		28.20	10	H: −20 Col_h 71 Iso	4.0
				16.32	11	C: Iso 70 Col_h −20	−4.1
**NBI2·Pt2** (5:1)	Col_h (25)	31.8		27.56	10	H: −20 Col_h 47 Iso	3.7
				15.91	11	C: Iso 60 Col_h −20	−3.4
**(NBI2:NBI1)·Pyr** (5:1)	Col_h (25)	32.5	372	28.15	10	H: −20 Col_h 73 Iso	4.5
(100:1)				16.28	11	C: Iso 72 Col_h −20	−4.5
**(NBI2:NBI1)·Pyr** (5:1)	Col_h (25)	32.1	378	27.82	10	H: −20 Col_h 77 Iso	5.5
(100:5)				16.08	11	C: Iso 78 Col_h −20	−5.8
**(NBI2:NBI1)·Pyr** (5:1)	Col_h (25)	31.8	372	27.53	10	H: −20 Col_h 89 Iso	7.4
(100:10)				15.90	11	C: Iso 90 Col_h −20	−6.7
**(NBI2:NBI1)·Pyr** (5:1)	Col_h (25)	30.7	328	26.57	10	H: −20 Col_h 98 Iso	8.7
(100:100)				15.38	11	C: Iso 97 Col_h −20	−8.2

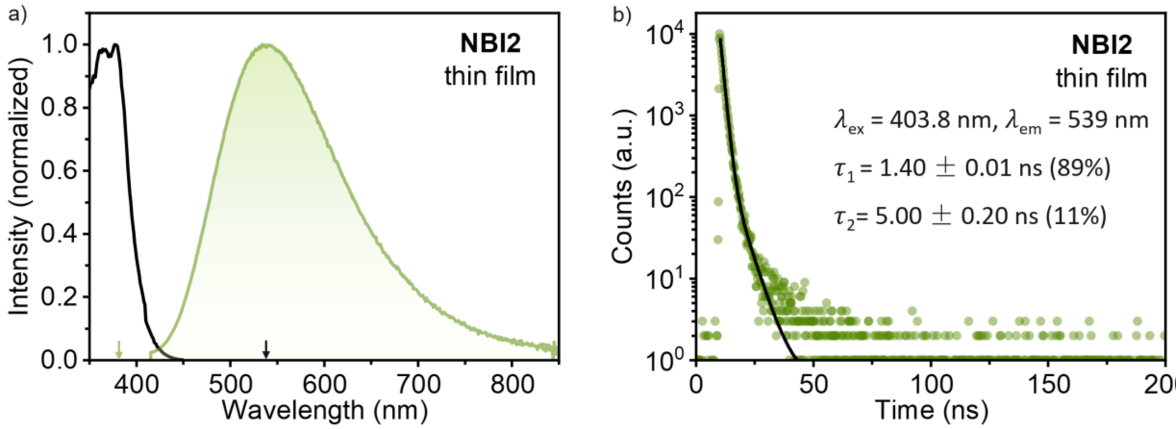

Figure S9. a) PL measurements of **NBI2** in the liquid crystal state of matter as thin film at room temperature on quartz substrate. Normalized excitation (black) and emission spectrum (green) of **NBI2** (λ_{em} = 539 nm, λ_{ex} = 380 nm). The arrows indicate λ_{em} of the excitation spectrum (black) and λ_{ex} of the emission spectrum (green). b) PL lifetime decay (symbol) of **NBI2** detected at the maximum of emission with the best fit (black line). The experimental conditions for lifetime measurements λ_{ex} and λ_{em} and the lifetime components of the decay are given next to the graph.

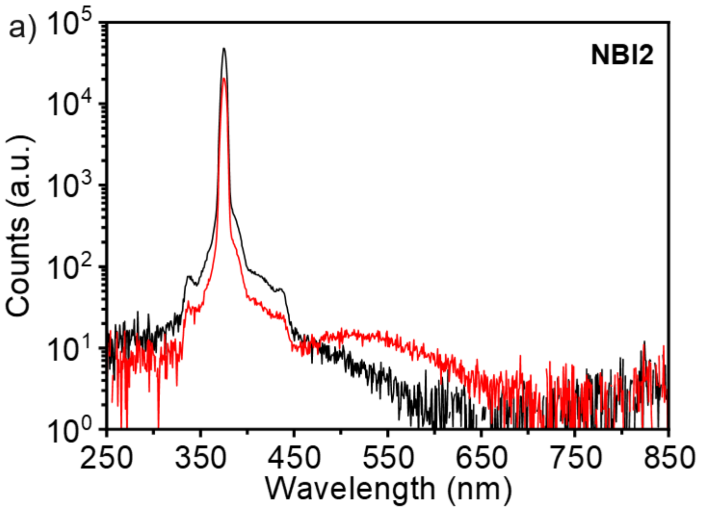

Figure S10. Optical profile of the integrating sphere measurement of reference (black line) and **NBI2** (red line) at ambient conditions upon excitation with λ_{ex} = 375 nm.

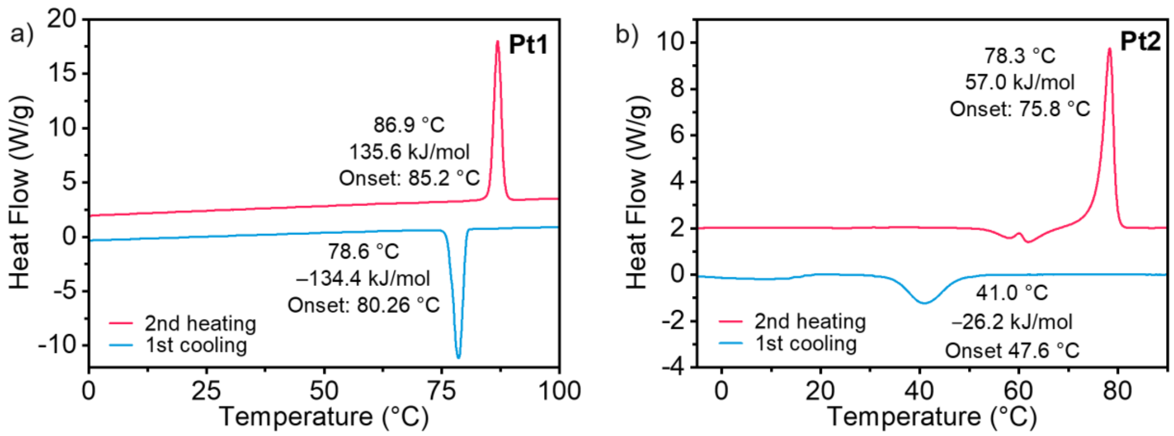

Figure S11. DSC traces of the Pt(II) complexes a) **Pt1** and b) **Pt2** from the second heating (red) and first cooling (blue) cycles with a rate of 10 K min^{-1}, endo up.

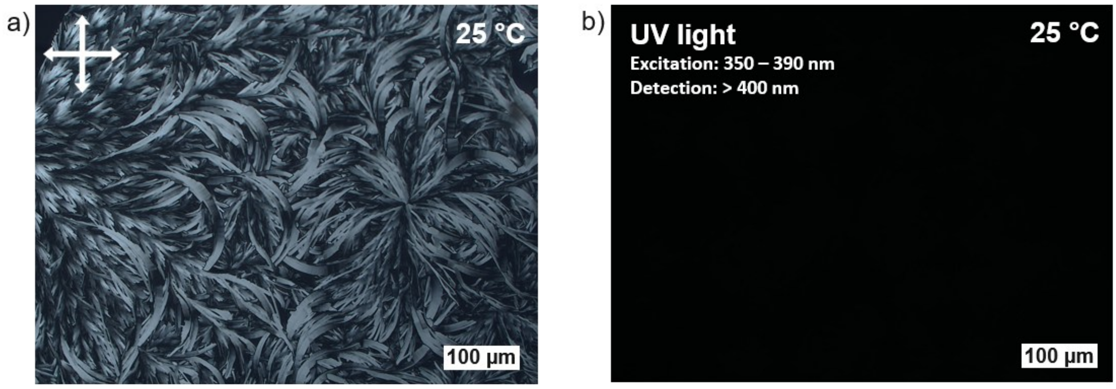

Figure S12. a) POM image of **Pt1** at 298 K recorded in transmission mode with crossed polarizers after heating to the isotropic liquid and b) PL-POM image recorded in reflection mode under UV light excitation showing no emission.

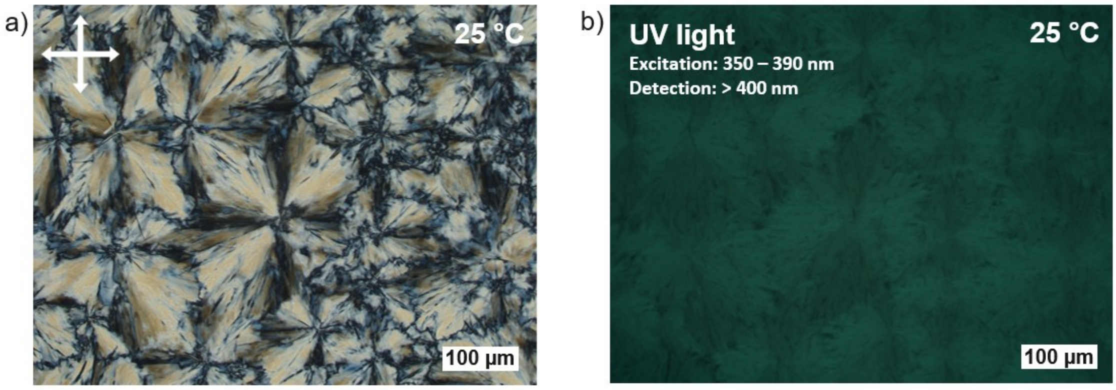

Figure S13. a) POM image of **Pt2** at 298 K recorded in transmission mode with crossed polarizers after heating to the isotropic liquid and b) PL-POM image recorded in reflection mode under UV light excitation showing weak emission.

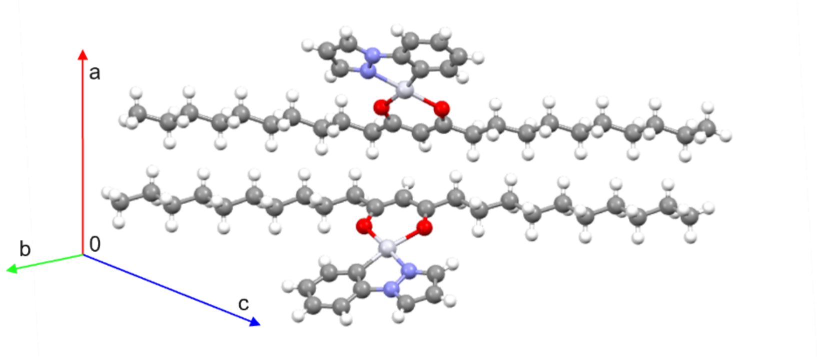

Figure S14. Molecular structure of **Pt2** according to single crystal X-ray analysis.

Table S2: Crystal structure and refinement data for a single crystal of **Pt2**.

	**Pt2**
**Empirical formula**	$C_{32}H_{50}N_2O_2Pt$
$\boldsymbol{M_{\mathrm{empirical}}}$ **[g mol^{-1}]**	689.83
Wavelength **[Å]**	1.54178
T **[K]**	100
Description of the crystal:	
**Color**	colourless
**Habit**	Needle
**Crystal System**	Monoclinic
**Space group**	P 21
Unit cell dimension:	
a **[Å]**	14.5828(14)
b **[Å]**	5.1262(5)
c **[Å]**	21.435(2)
a **[°]**	90
b **[°]**	109.748(3)
g **[°]**	90
Volume **[Å^3]**	1508.1(3)
Z	2
$\rho_{\mathrm{alc.}}$ **[g cm^{-3}]**	1519
***F*(000)**	700
Goodness of Fit	1.062
**CCDC**	2494985

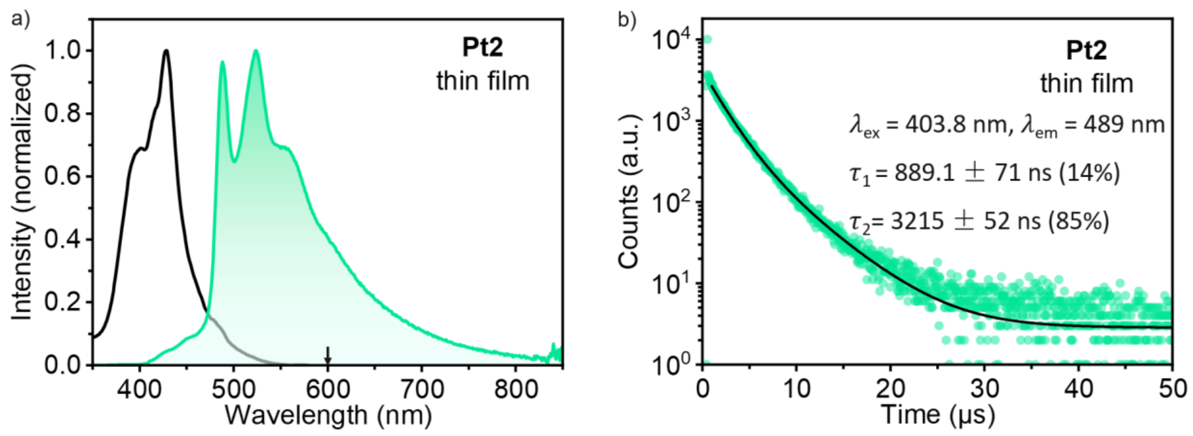

Figure S15. a) PL measurement for **Pt2** as thin films at room temperature on quartz substrates. Normalized excitation (black) and emission spectrum (green) (λ_{em} = 600 nm, λ_{ex} = 320 nm). The arrow indicates λ_{em} of the excitation spectrum (black). b) PL lifetime decay (symbol) detected at the maximum of emission with the best fit (black line). The experimental conditions for lifetime measurements λ_{ex} and λ_{em} and the lifetime components of the decay are given next to the graph.

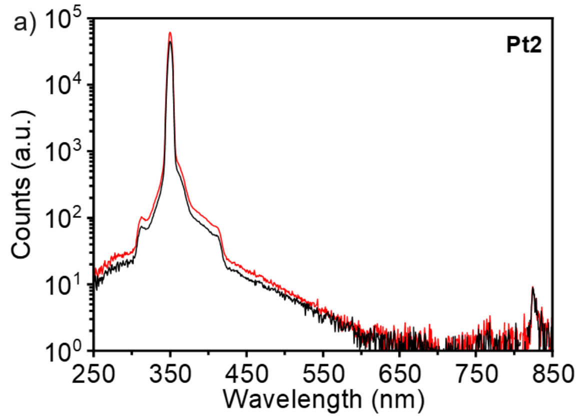

Figure S16. Optical profile of the integrating sphere measurement of reference (black line) and **Pt2** (red line) at ambient conditions upon excitation with λ_{ex} = 350 nm.

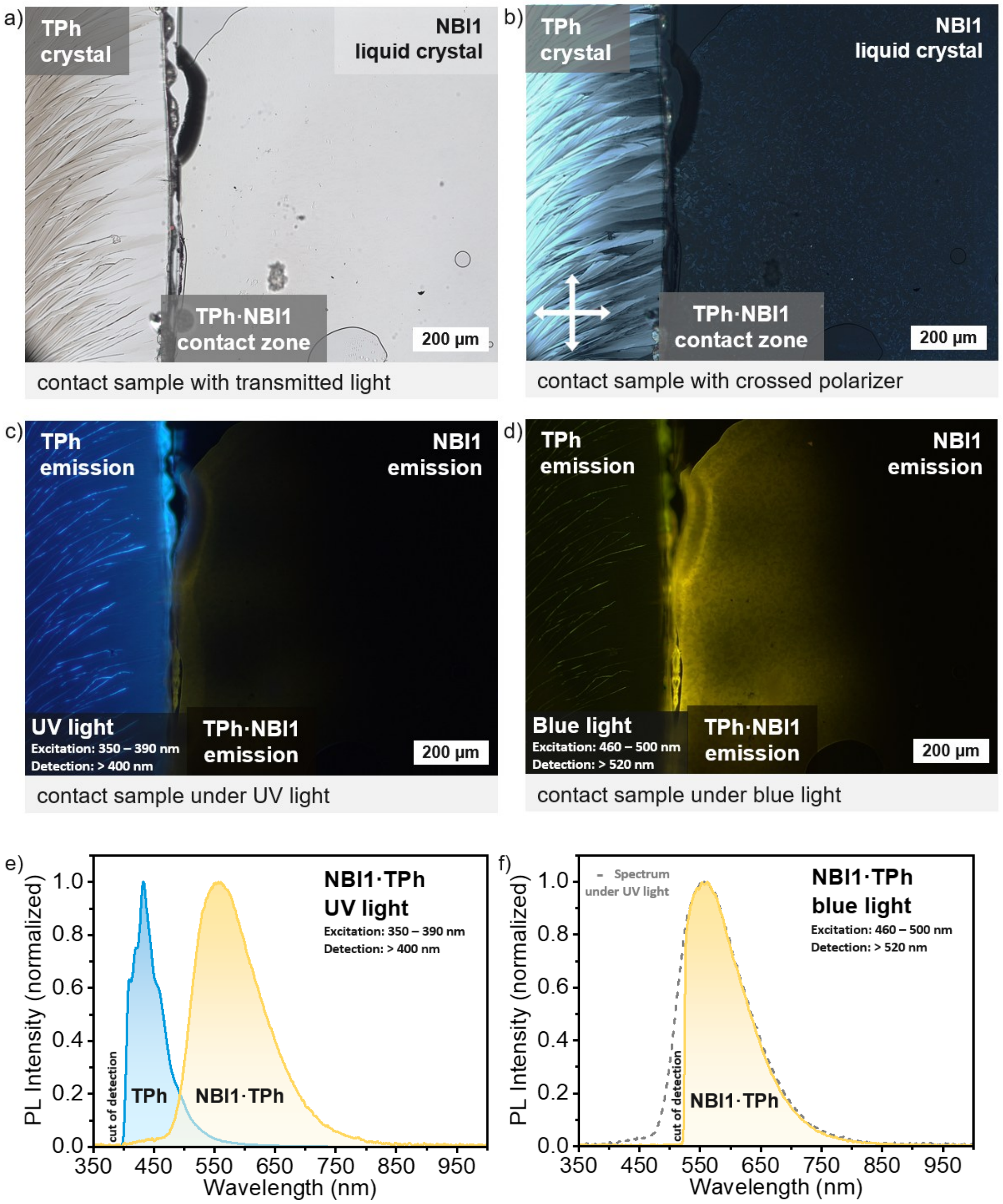

Figure S17. Contact experiment of **NBI1** host and **TPh** guest at ambient conditions (298 K) after melting the substances together. Contact zone recorded in transmission mode a) without polarizers and b) with crossed polarizers. Contact zone recorded in reflection mode c) under UV light and d) under blue light excitation. Emission spectra (not corrected) of **TPh** (blue) and **NBI1·TPh** contact zone (yellow) e) under UV light and f) under blue light irradiation of the sample shown in c) and d), respectively. The cut of detection by the filter is visible in e) and f).

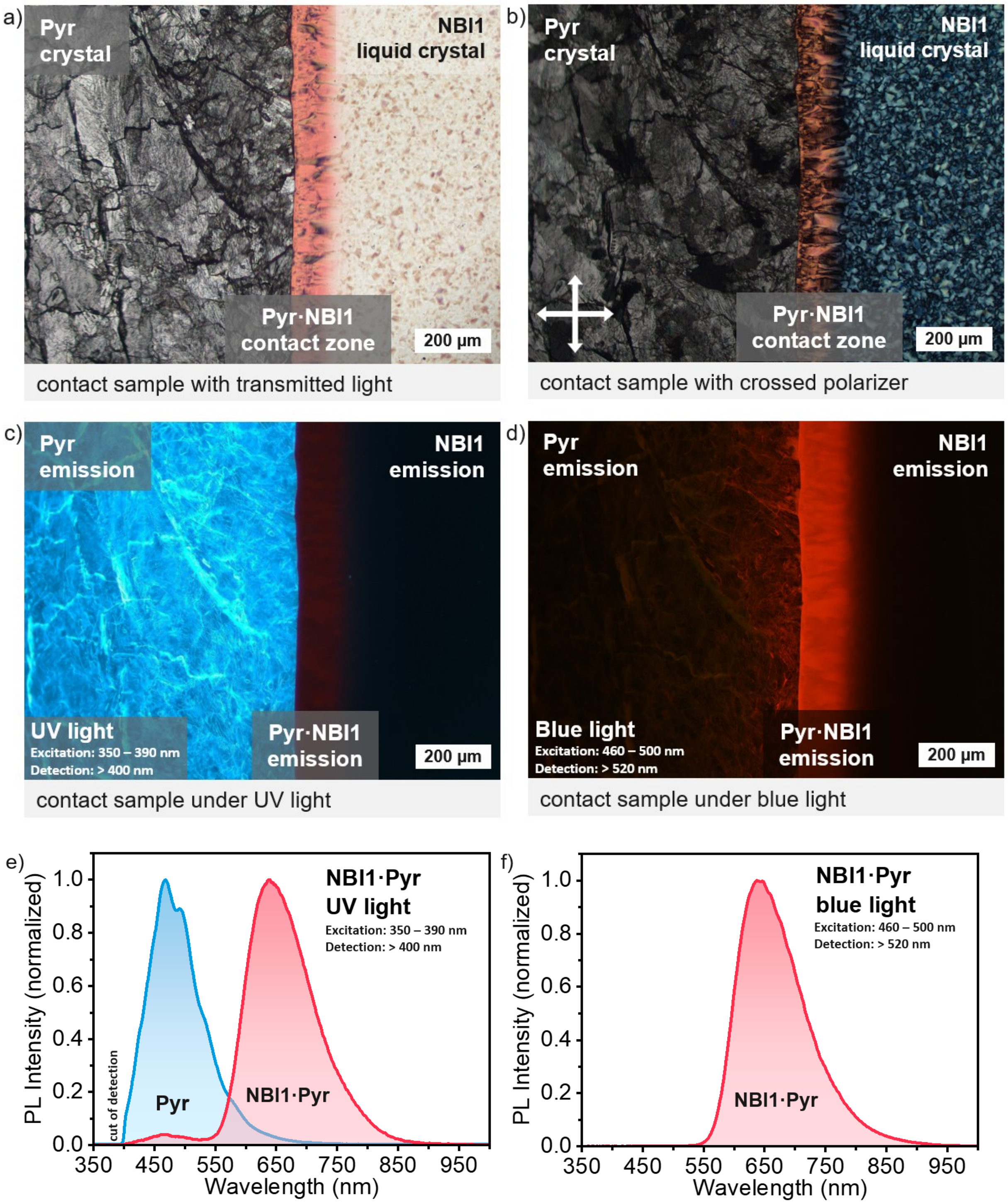

Figure S18. Contact experiment of **NBI1** host and **Pyr** guest at ambient conditions (298 K) after melting the substances together. Contact zone recorded in transmission mode a) without polarizers and b) with crossed polarizers. Contact zone recorded in reflection mode c) under UV light and d) under blue light excitation. Emission spectra (not corrected) of **Pyr** (blue) and **NBI1·Pyr** contact zone (red) e) under UV light and f) under blue light of the sample shown in c) and d), respectively. The cut of detection by the filter is visible in e).

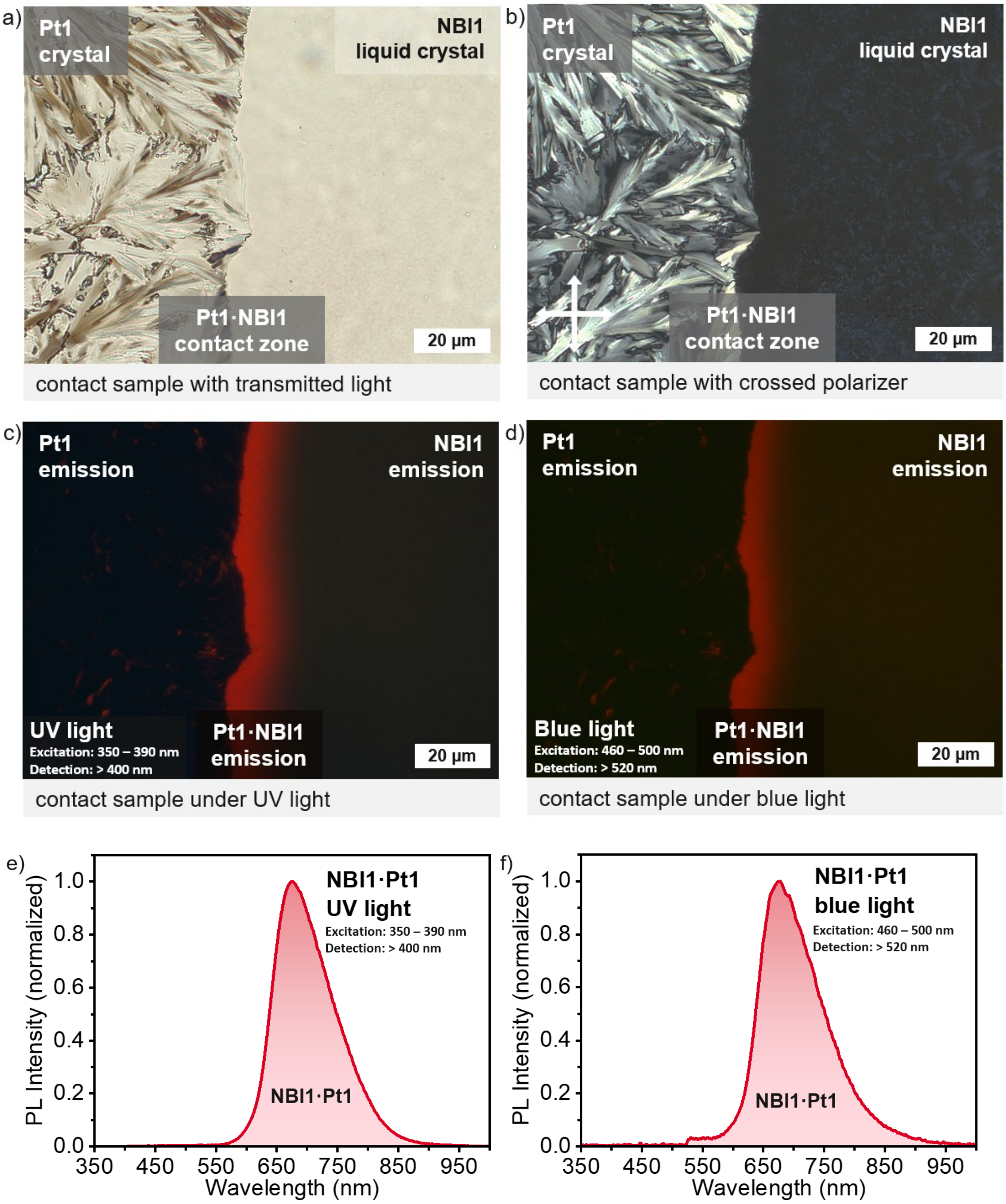

Figure S19. Contact experiment of **NBI1** host and **Pt1** guest at ambient conditions (298 K) after melting the substances together. Contact zone recorded in transmission mode a) without polarizers and b) with crossed polarizers. Contact zone recorded in reflection mode c) under UV light and d) under blue light excitation. Emission spectra (not corrected) of the **NBI1·Pt1** contact zone (dark red) e) under UV light and f) under blue light of the sample shown in c) and d), respectively.

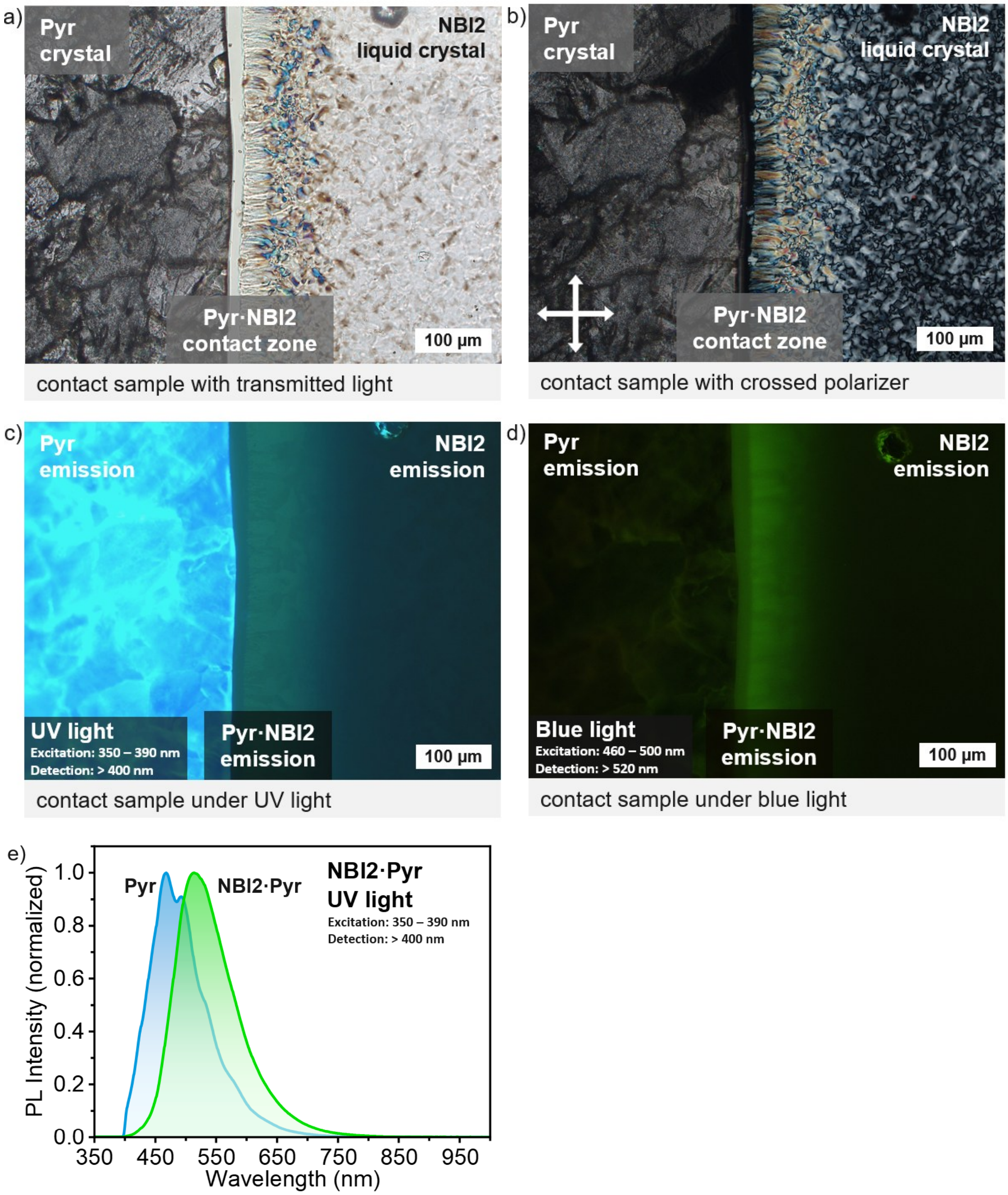

Figure S20. Contact experiment of **NBI2** host and **Pyr** guest at ambient conditions (298 K) after melting the substances together. Contact zone recorded in transmission mode a) without polarizers and b) with crossed polarizers. Contact zone recorded in reflection mode c) under UV light and d) under blue light excitation. Emission spectra (not corrected) of **Pyr** (blue) and **NBI2·Pyr** contact zone (green) e) under UV light of the sample shown in c).

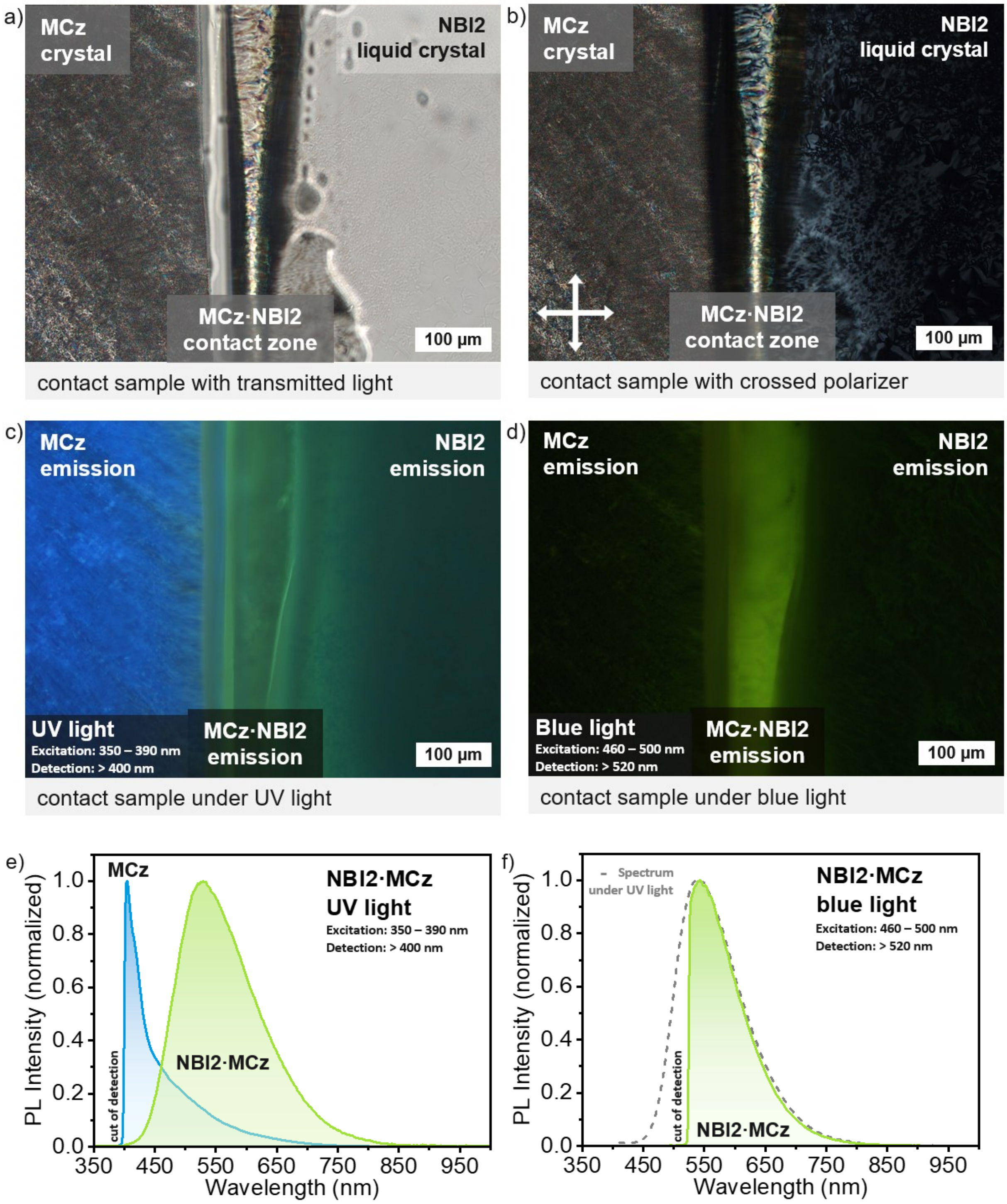

Figure S21. Contact experiment of **NBI2** host and **MCz** guest at ambient conditions (298 K) after melting the substances together. Contact zone recorded in transmission mode a) without polarizers and b) with crossed polarizers. Contact zone recorded in reflection mode c) under UV light and d) under blue light excitation. Emission spectra (not corrected) of **MCz** (blue) and **NBI2·MCz** contact zone (light green) e) under UV light and f) under blue light of the sample shown in c) and d), respectively. The cut of detection by the filter is visible in e) and f).

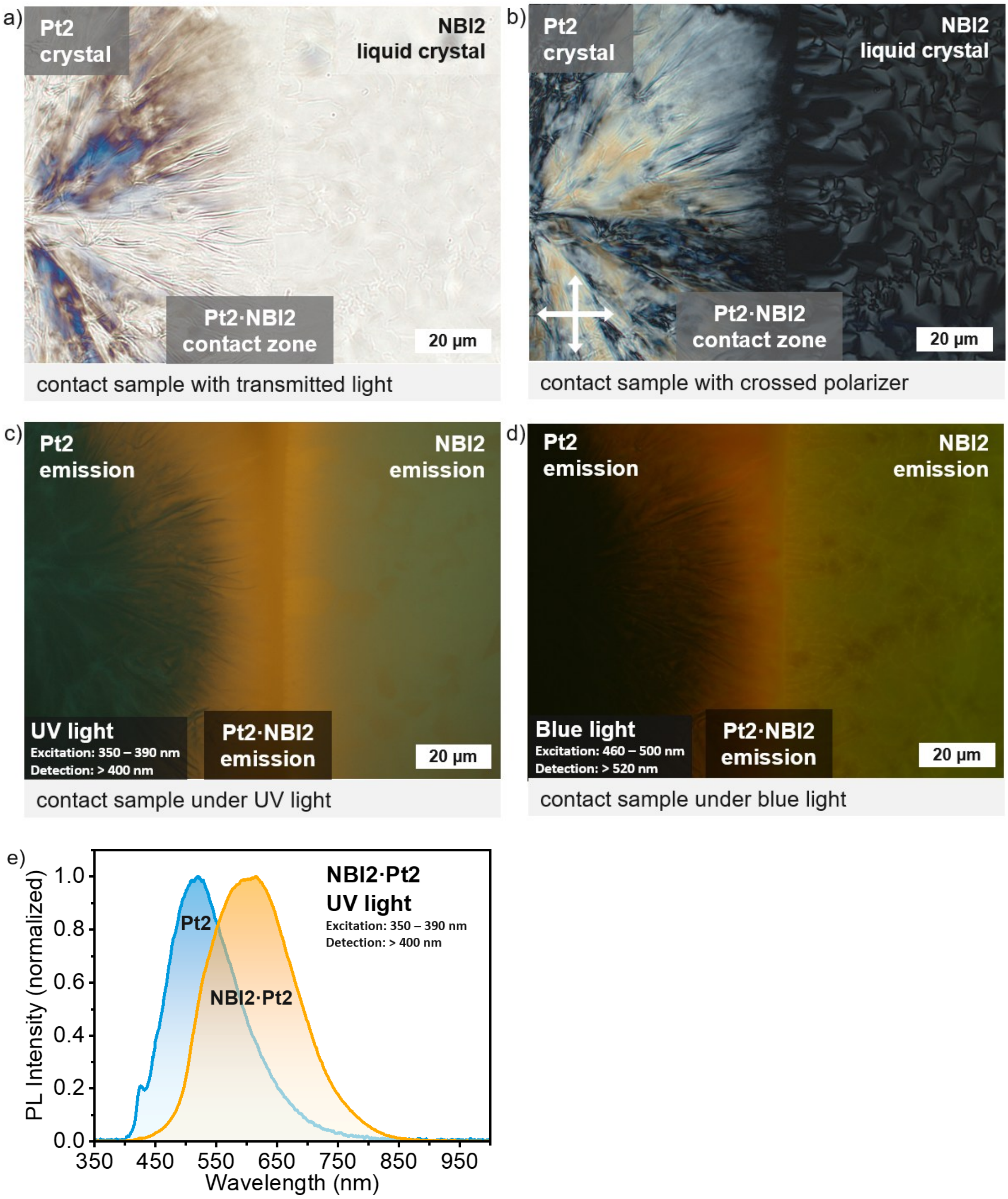

Figure S22. Contact experiment of **NBI2** host and **Pt2** guest at ambient conditions (298 K) after melting the substances together. Contact zone recorded in transmission mode a) without polarizers and b) with crossed polarizers. Contact zone recorded in reflection mode c) under UV light and d) under blue light excitation. Emission spectra (not corrected) of **Pt2** (blue) and **NBI2·Pt2** contact zone (orange) e) under UV light of the sample shown in c).

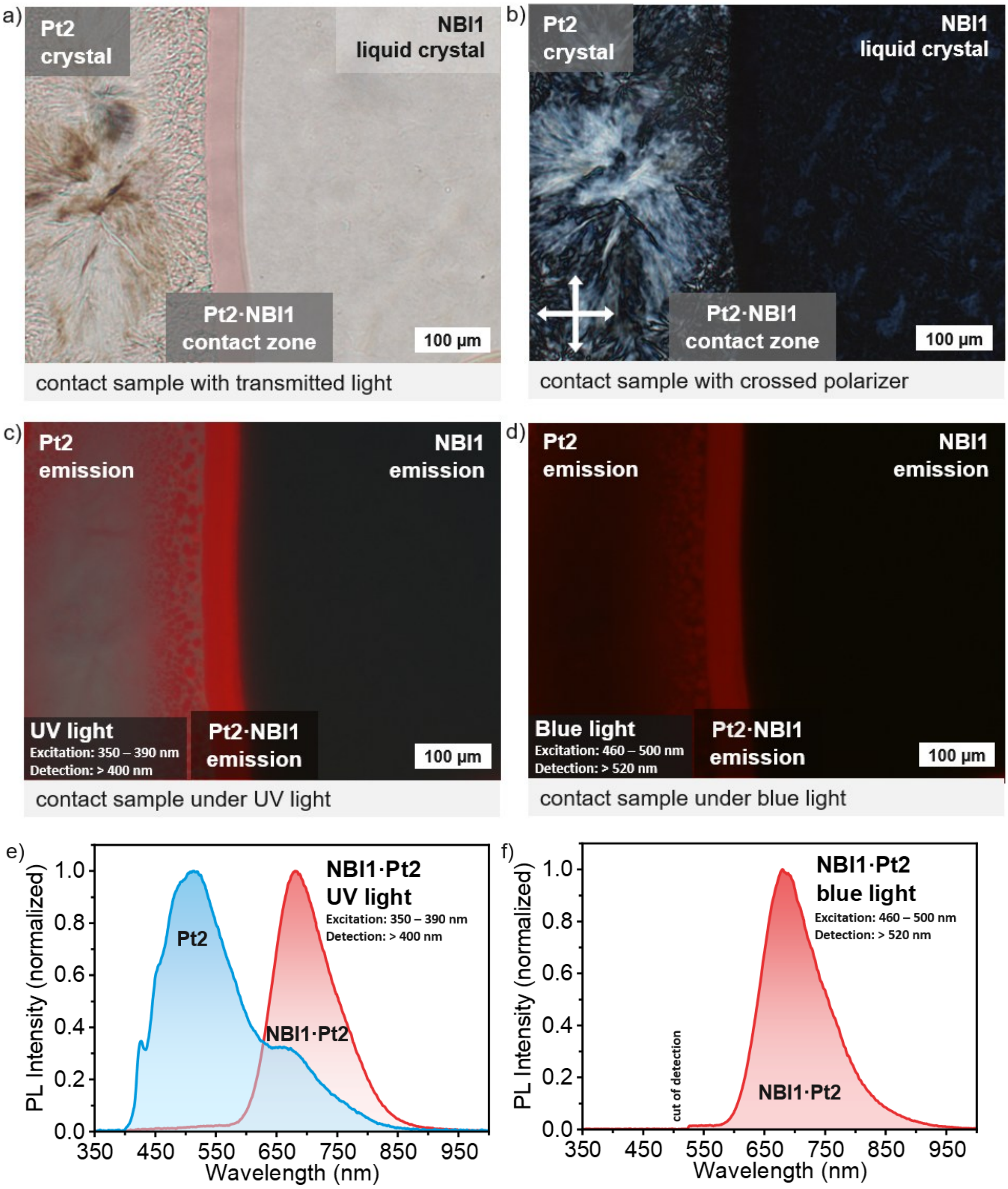

Figure S23. Contact experiment of **NBI1** host and **Pt2** guest at ambient conditions (298 K) after melting the substances together. Contact zone recorded in transmission mode a) without polarizers and b) with crossed polarizers. Contact zone recorded in reflection mode c) under UV light and d) under blue light excitation. Emission spectra (not corrected) of **Pt2** and overshine of the contact zone (blue) and **NBI1·Pt2** contact zone (red) e) under UV light and f) under blue light of the sample shown in c) and d), respectively. The cut of detection by the filter is visible in f).

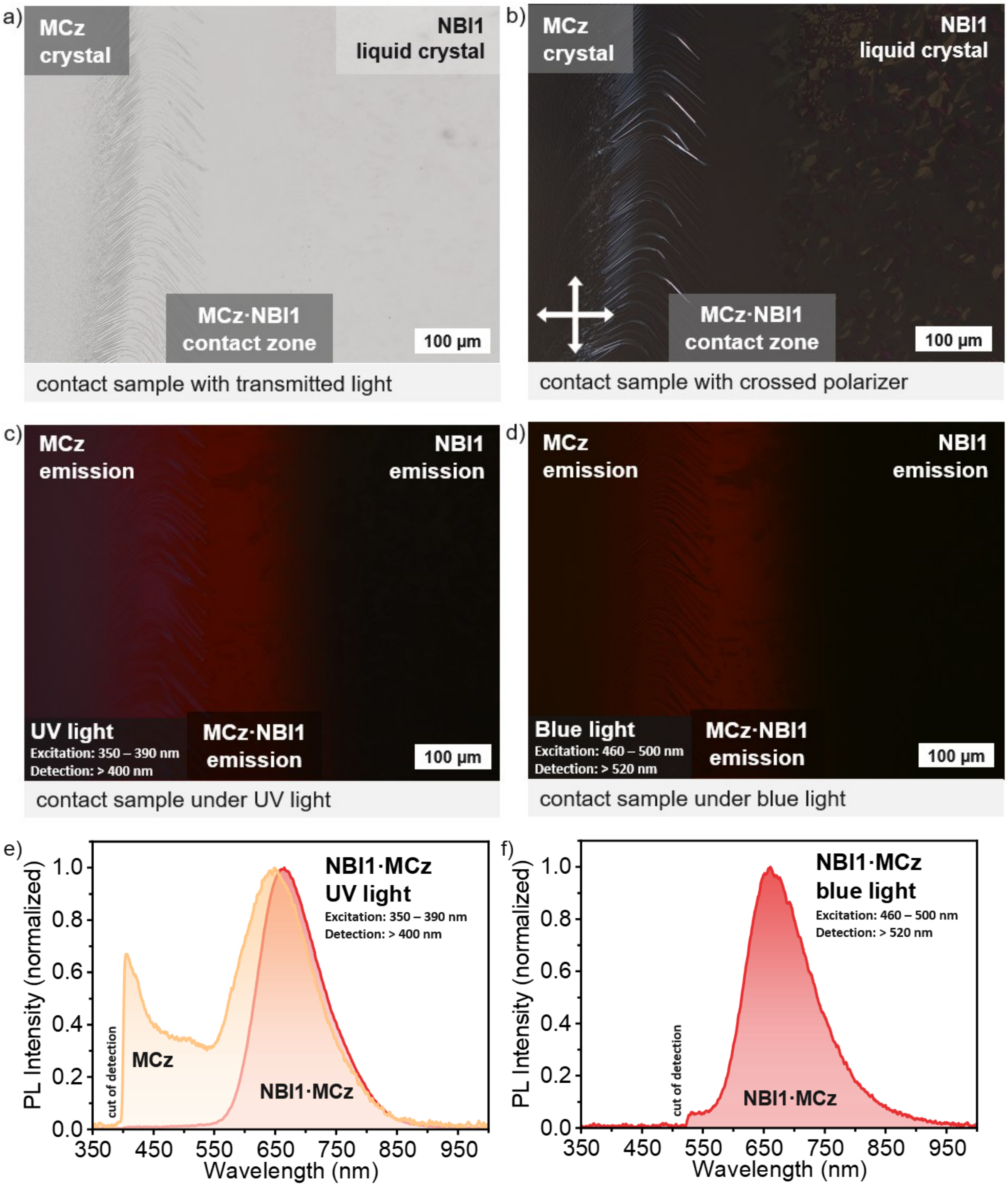

Figure S24. Contact experiment of **NBI1** host and **MCz** guest at ambient conditions (298 K) after melting the substances together. Contact zone recorded in transmission mode a) without polarizers and b) with crossed polarizers. Contact zone recorded in reflection mode c) under UV light and d) under blue light excitation. Emission spectra (not corrected) of **MCz** and overshine of the contact zone (blue) and **NBI1·MCz** contact zone (red) e) under UV light and f) under blue light of the sample shown in c) and d), respectively. The cut of detection by the filter is visible in e) and f).

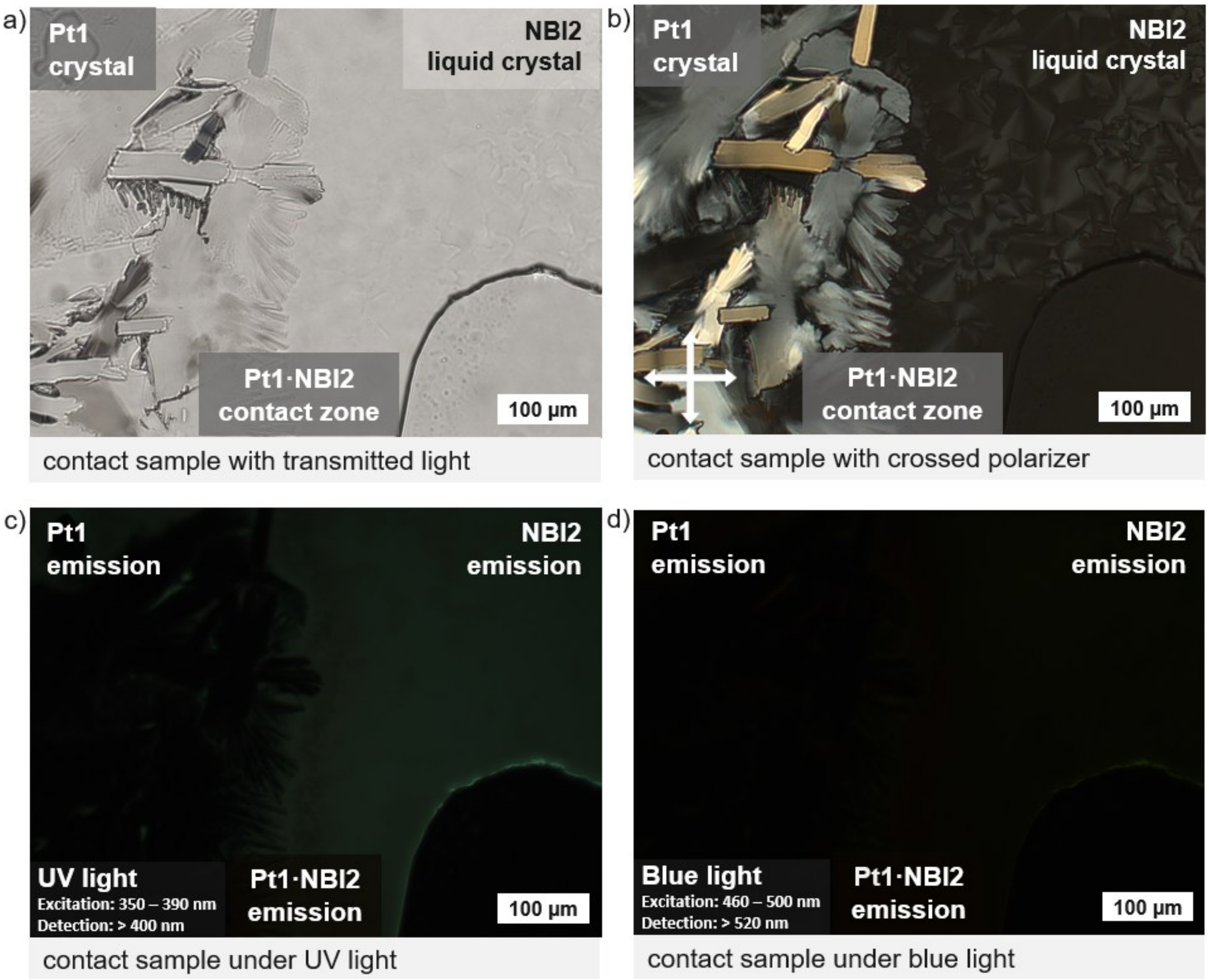

Figure S25. Contact experiment of **NBI2** host and **Pt1** guest at ambient conditions (298 K) after melting the substances together. Contact zone recorded in transmission mode a) without polarizers and b) with crossed polarizers. Contact zone recorded in reflection mode c) under UV light and d) under blue light excitation. The contact experiment does not show emission from the contact zone of the substances.

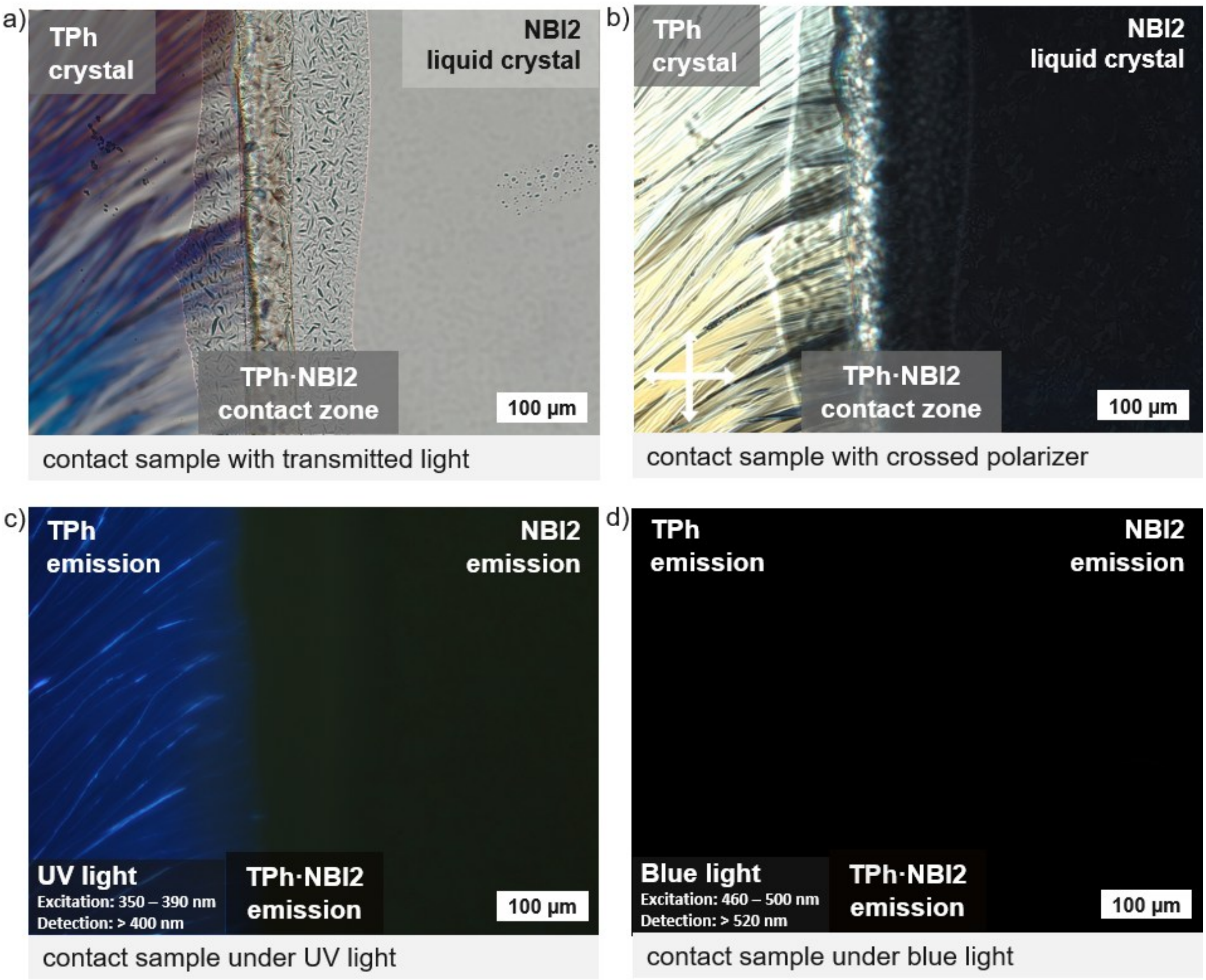

Figure S26. Contact experiment of **NBI2** host and **TPh** guest at ambient conditions (298 K) after melting the substances together. Contact zone recorded in transmission mode a) without polarizers and b) with crossed polarizers. Contact zone recorded in reflection mode c) under UV light and d) under blue light excitation. The contact experiment does not show emission from the contact zone of the substances.

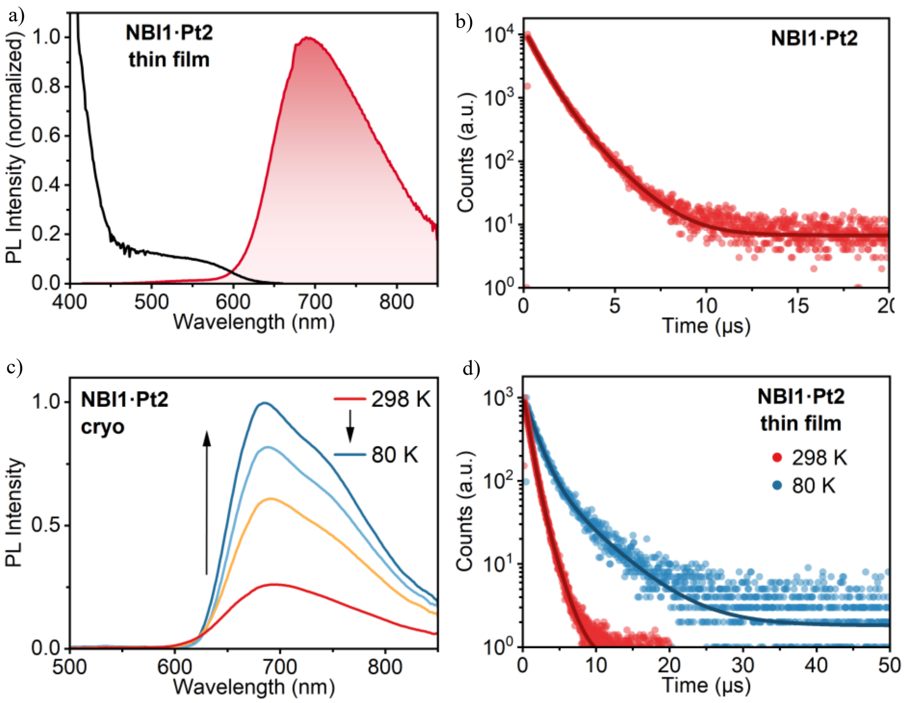

298 K:

λ_{ex} = 505.8 nm, λ_{em} = 691 nm

τ_1 = 0.71 ± 0.01 μs (65%)

τ_2 = 1.50 ± 0.05 μs (35%)

80 K:

λ_{ex} = 505.8 nm, λ_{em} = 691 nm

τ_1 = 1.46 ± 0.04 μs (58%)

τ_2 = 5.20 ± 0.15 μs (42%)

Figure S27. PL measurements of the LC mixture **NBI1·Pt2** (5:1) as thin film at room temperature on quartz substrate. a) Normalized excitation (black line) and emission spectrum (in the color of emission, λ_{em} = 691 nm, λ_{ex} = 400 nm). b) PL lifetime decay (symbol) with the best fit (solid line). The experimental conditions for lifetime measurements λ_{ex} and λ_{em} and the lifetime components of the decay are given next to the graph. c) Relative PL intensities of the temperature-dependent PL measurements displayed at 80 K (dark blue), 150 K (light blue), 220 K (orange) and 298 K (red) with λ_{ex} = 400 nm and d) PL lifetime decay at 298 K (symbol, red) and 80 K (symbol, blue) and the best fit (solid lines). The experimental conditions for lifetime measurements λ_{ex} and λ_{em} and the lifetime components of the decay are given next to the graph.

Table S3. Comparison of the different **NBI1** and **NBI2** LC host–guest mixtures and their corresponding PL properties.

Guest \ Host	**NBI1**<br>Photoluminescence	**NBI2**<br>Photoluminescence
**Pyr**	Red TADF	Green Eciplex
**MCz**	Red (very weak)	Green TADF
**TPh**	Yellow Exciplex	No
**Pt1**	Red TADF + RTP	No
**Pt2**	Red TADF + RTP (weak)	Orange TADF + RTP

Table S4. Host–guest ratio dependent Φ_{PL} and phase behavior of **NBI1·Pyr** and **NBI1·Pt1**.

Material	Φ_{PL} [%]	LC	Material	Φ_{PL} [%]	LC
**NBI1·Pyr** (1:1)	3	No	**NBI1·Pt1** (1:1)	3	No
**NBI1·Pyr** (2:1)	3	No	**NBI1·Pt1** (2:1)	4	No
**NBI1·Pyr** (5:1)	3	Yes	**NBI1·Pt1** (5:1)	4	Yes
**NBI1·Pyr** (10:1)	2	Yes	**NBI1·Pt1** (10:1)	3	Yes
**NBI1·Pyr** (100:1)	2	Yes	**NBI1·Pt1** (100:1)	2	Yes

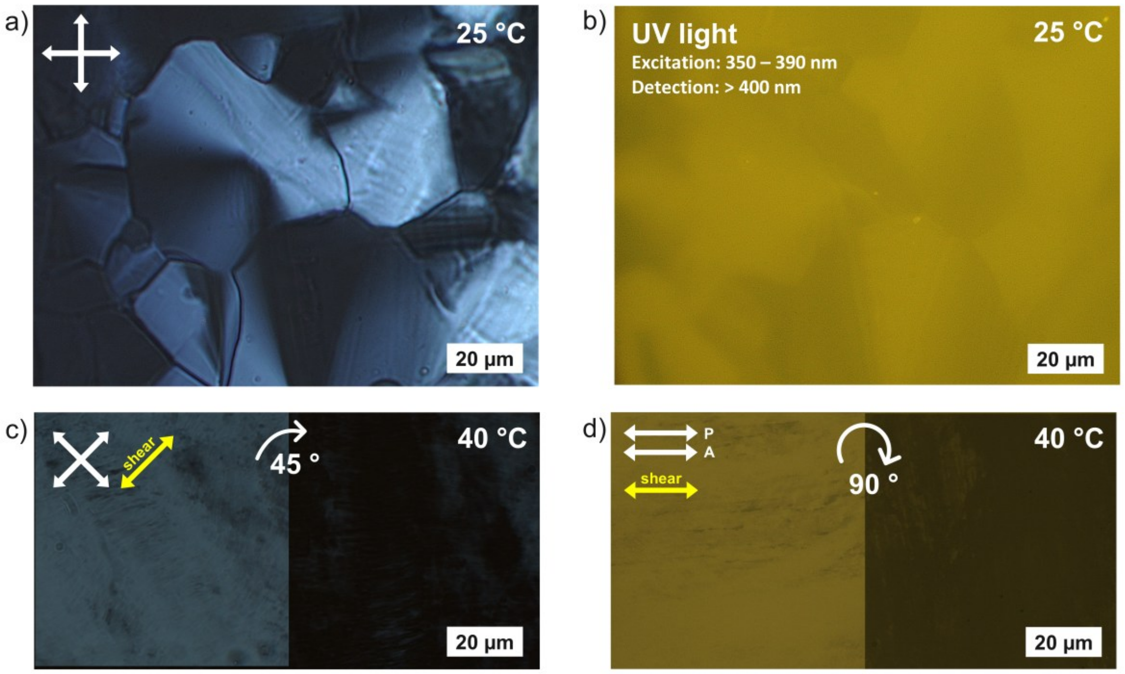

Figure S28. POM images of **NBI1·TPh** (5:1) recorded in transmission mode with crossed polarizers of a) LC texture at 25 °C after melting and c) aligned sample by shearing at 40 °C. PL-POM image recorded in reflection mode under UV light irradiation of b) PL of texture and d) linear polarized light of aligned sample. Direction of shear is indicated with a yellow arrow.

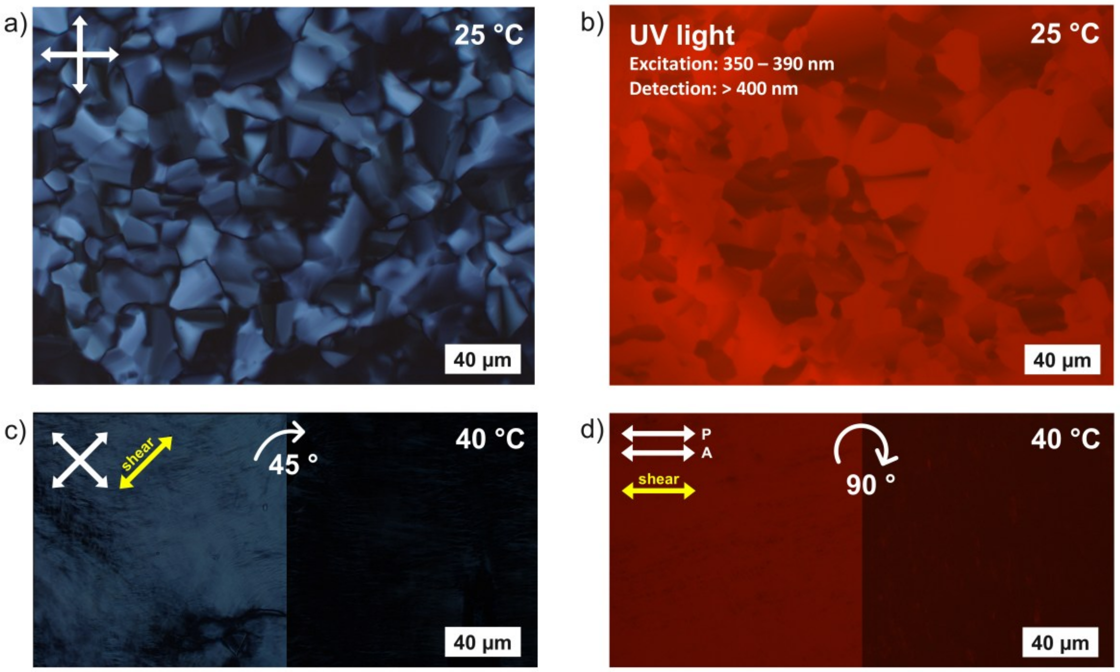

Figure S29. POM images of **NBI1·Pyr** (5:1) recorded in transmission mode with crossed polarizers of a) LC texture at 25 °C after melting and c) aligned sample by shearing at 40 °C. PL-POM image recorded in reflection mode under UV light irradiation of b) PL of texture and d) linear polarized light of aligned sample. Direction of shear is indicated with a yellow arrow.

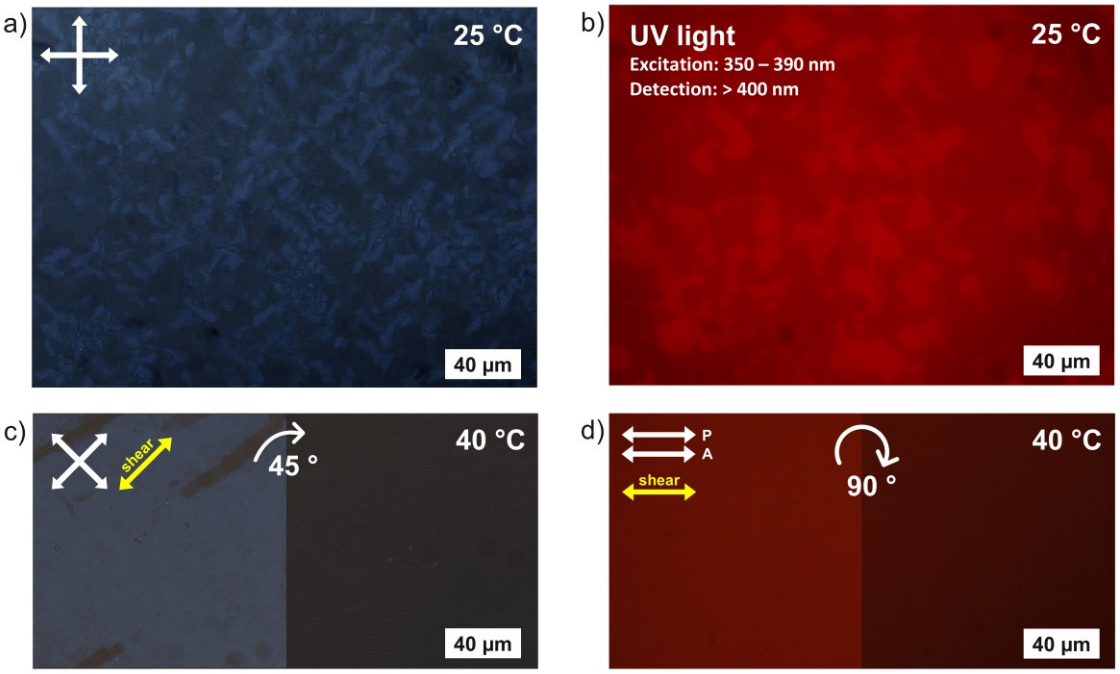

Figure S30. POM images of **NBI1·Pt1** (5:1) recorded in transmission mode with crossed polarizers of a) LC texture at 25 °C after melting and c) aligned sample by shearing at 40 °C. PL-POM image recorded in reflection mode under UV light irradiation of b) PL of texture and d) linear polarized light of aligned sample. Direction of shear is indicated with a yellow arrow.

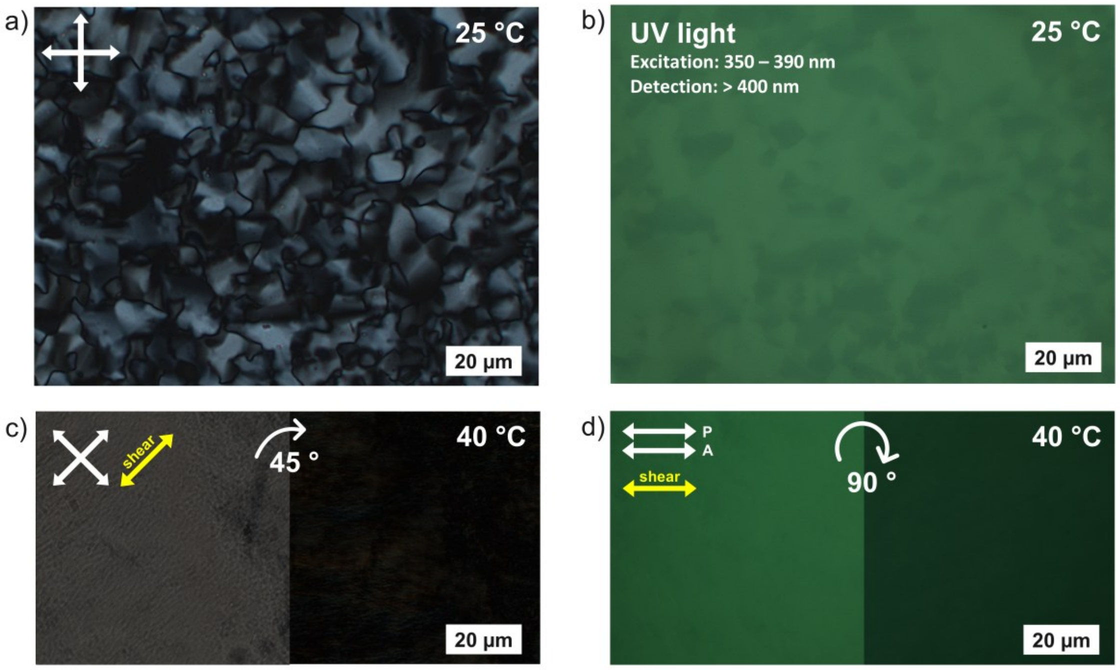

Figure S31. POM images of **NBI2·Pyr** (5:1) recorded in transmission mode with crossed polarizers of a) LC texture at 25 °C after melting and c) aligned sample by shearing at 40 °C. PL-POM image recorded in reflection mode under UV light irradiation of b) PL of texture and d) linear polarized light of aligned sample. Direction of shear is indicated with a yellow arrow.

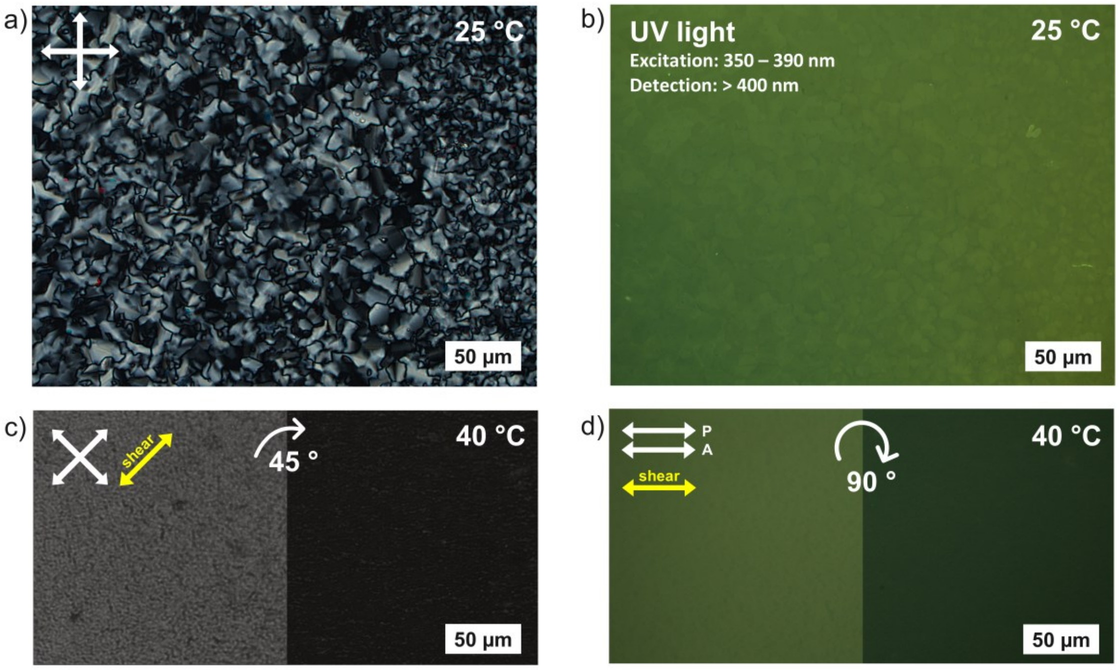

Figure S32. POM images of **NBI2·MCz** (5:1) recorded in transmission mode with crossed polarizers of a) LC texture at 25 °C after melting and c) aligned sample by shearing at 40 °C. PL-POM image recorded in reflection mode under UV light irradiation of b) PL of texture and d) linear polarized light of aligned sample. Direction of shear is indicated with a yellow arrow.

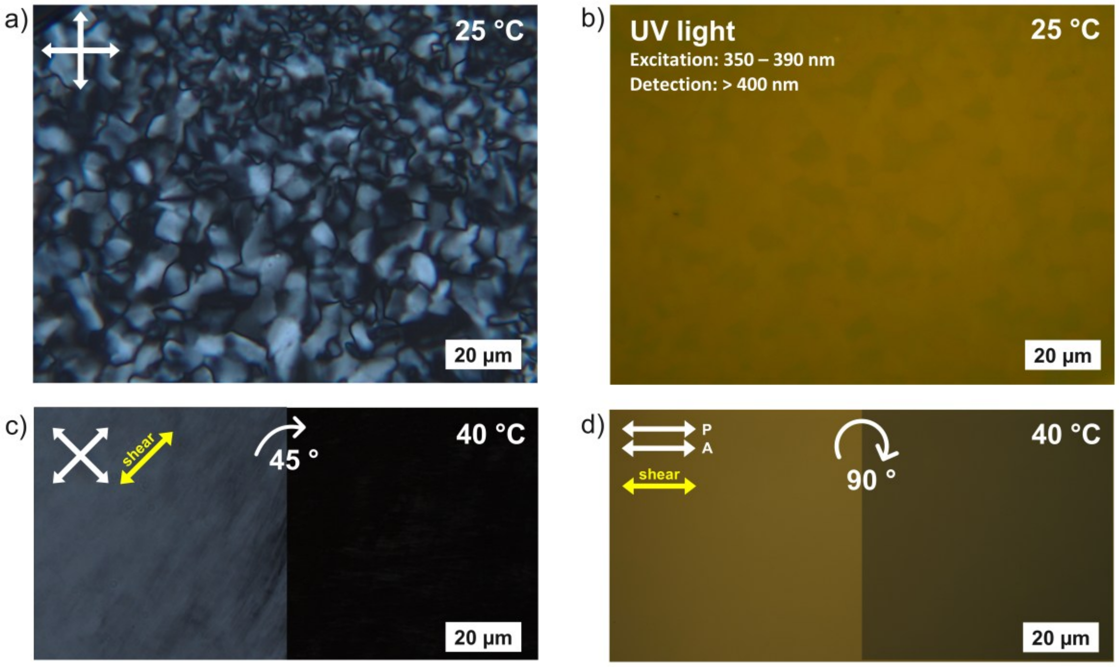

Figure S33. POM images of **NBI2·Pt2** (5:1) recorded in transmission mode with crossed polarizers of a) LC texture at 25 °C after melting and c) aligned sample by shearing at 40 °C. PL-POM image recorded in reflection mode under UV light irradiation of b) PL of texture and d) linear polarized light of aligned sample. Direction of shear is indicated with a yellow arrow.

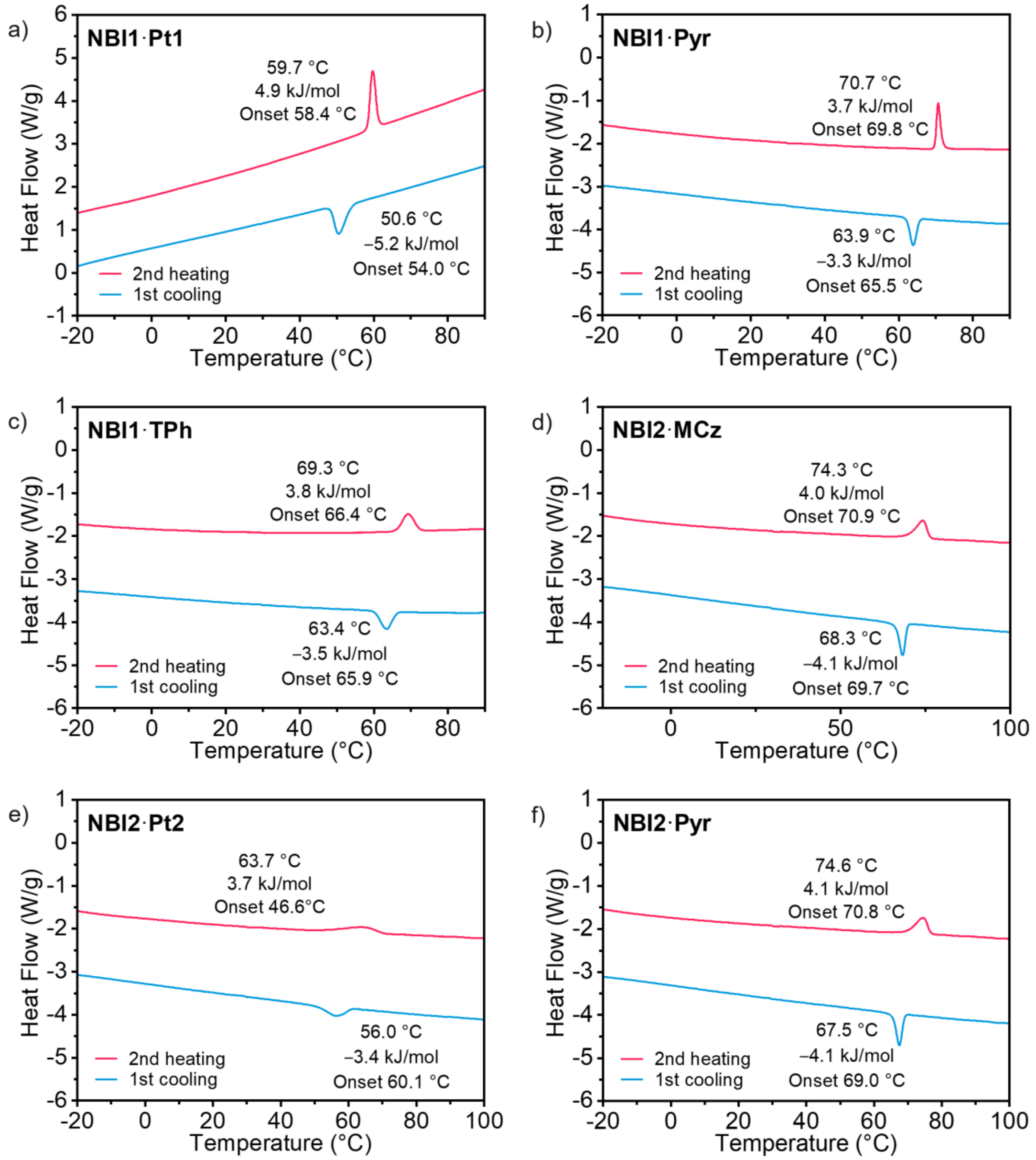

Figure S34. DSC traces of the mixtures (5:1) of a) **NBI1·Pt1**, b) **NBI1·Pyr**, c) **NBI1·TPh**, d) **NBI2·MCz**, e) **NBI2·Pt2** and f) **NBI2·Pyr** from the second heating (red) and first cooling (blue) cycles with a rates of 10 K min^{-1}, endo up.

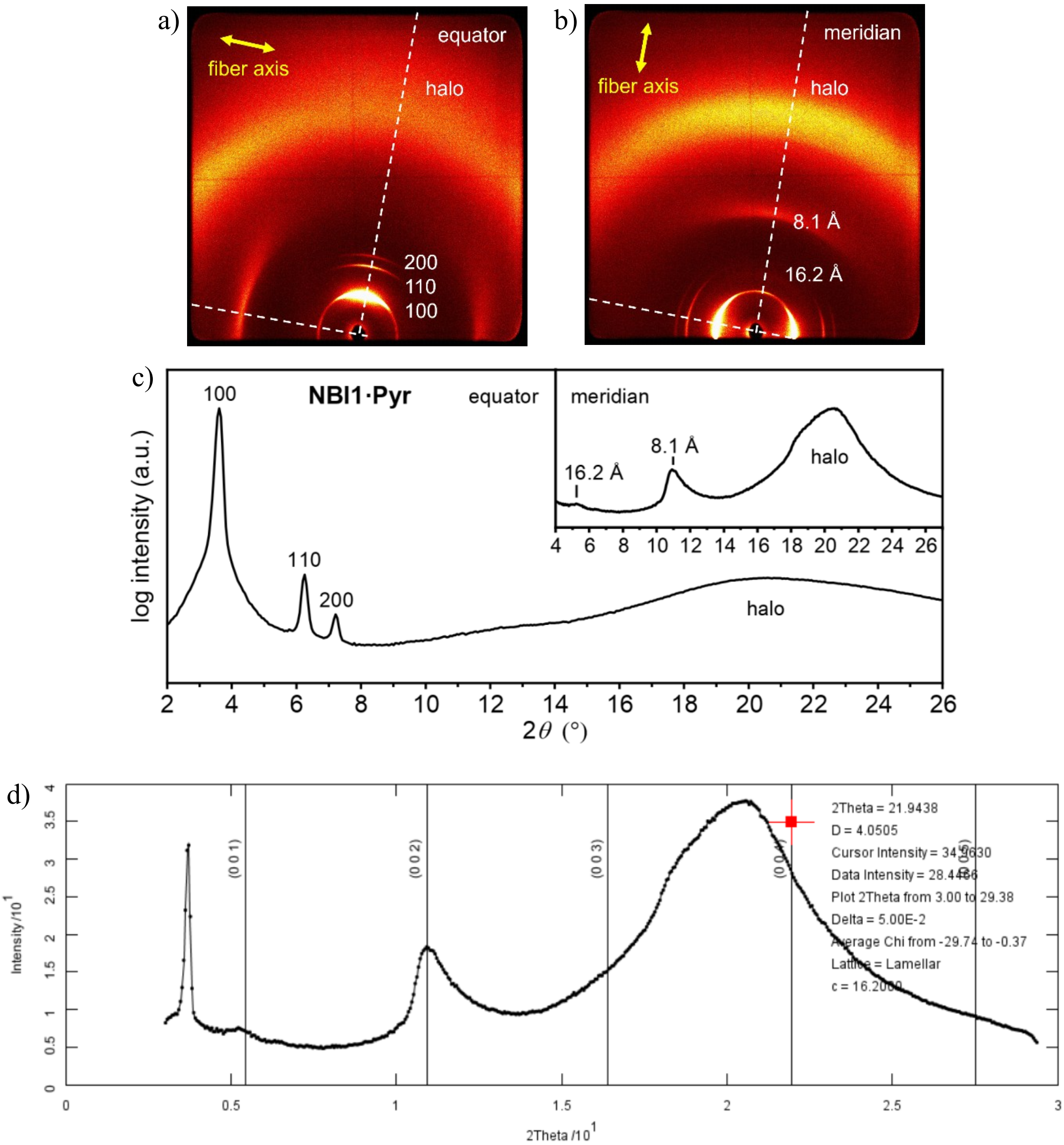

Figure S35. WAXS patterns of **NBI1·Pyr** (5:1) at 298 K of a) a lying fiber and b) a standing fiber. The position of the equator and meridian are indicated with white dashed lines. The direction of the fiber is indicated by yellow arrows. c) Integrated intensities along the equator and meridian (inlay) of the WAXS pattern. d) Integrated intensities along the meridian with lamellar order in the columns. The red cursor is located at 4.05 Å.

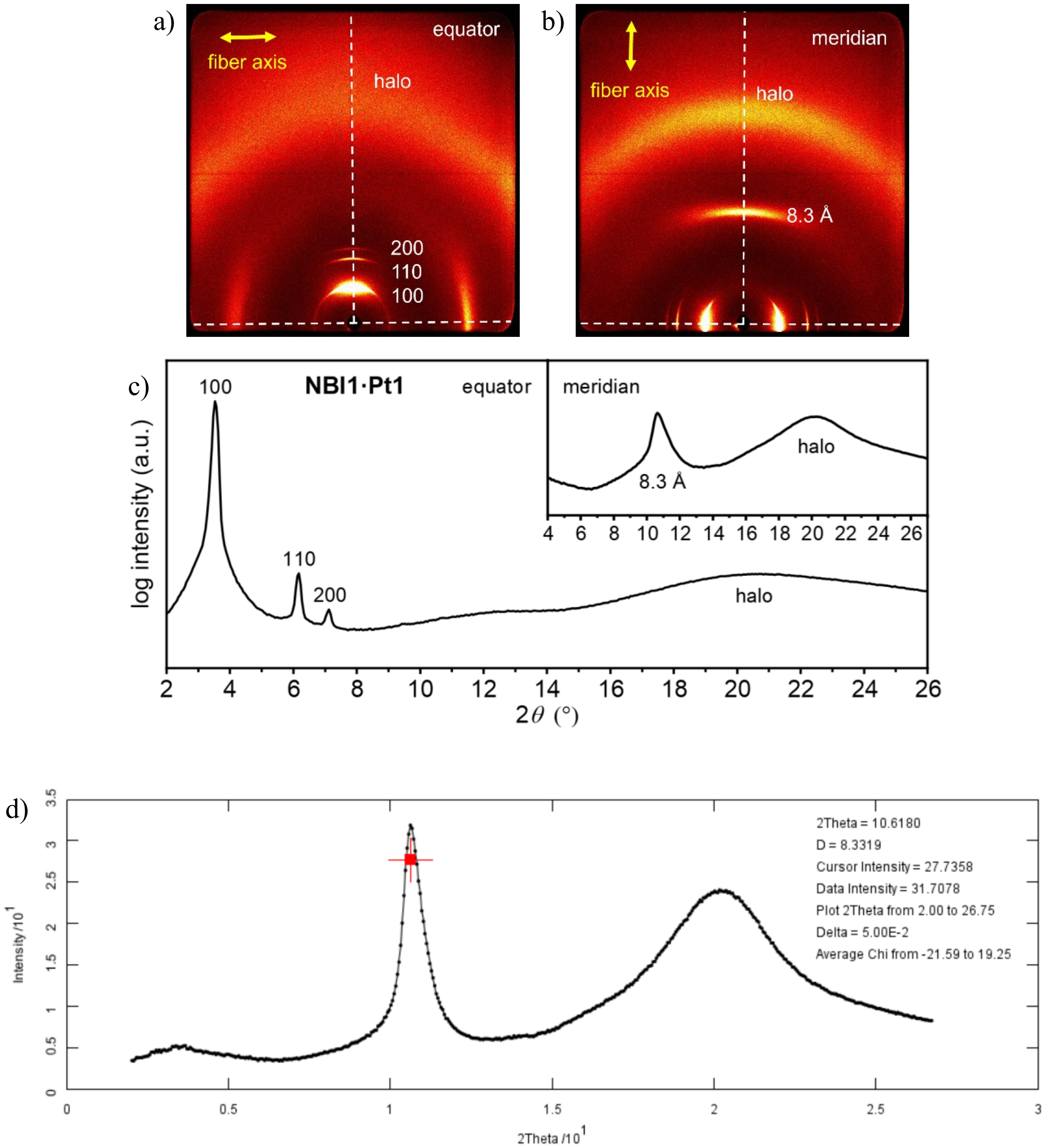

Figure S36. WAXS patterns of **NBI1·Pt1** (5:1) at 298 K of a) a lying fiber and b) a standing fiber. The position of the equator and meridian are indicated with white dashed lines. The direction of the fiber is indicated by yellow arrows. c) Integrated intensities along the equator and meridian (inlay) of the WAXS pattern. d) Integrated intensities along the meridian with lamellar order in the columns. The red cursor is located at 8.33 Å.

a)

b)

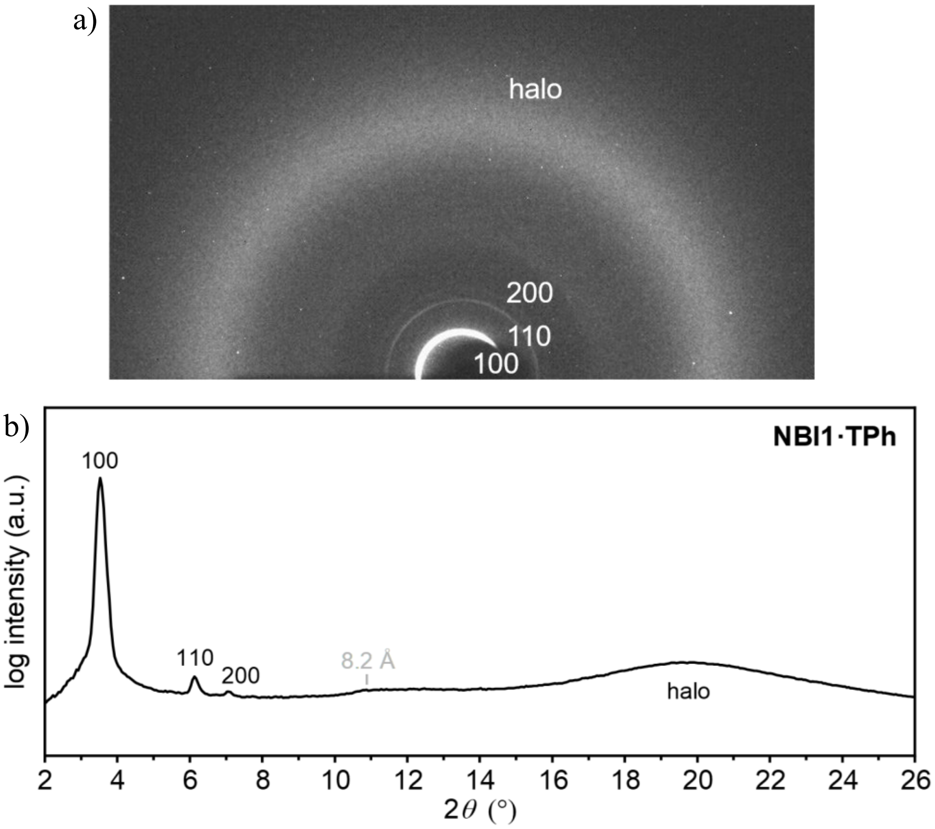

Figure S37. WAXS pattern of a) unaligned **NBI1·TPh** (5:1) at 298 K and b) integrated intensities. The peak on the meridian of the aligned host **NBI1** assigned to intracolumnar order is shown in gray. Random spots in the pattern (zingers) are sudden, localized energy-deposition events that appear as isolated, spurious signals caused by high-energy radiation (e.g., cosmic rays or scattered X-rays) and are unrelated to the true measurement; upon intensity integration, these spots disappear.[S9]

a)

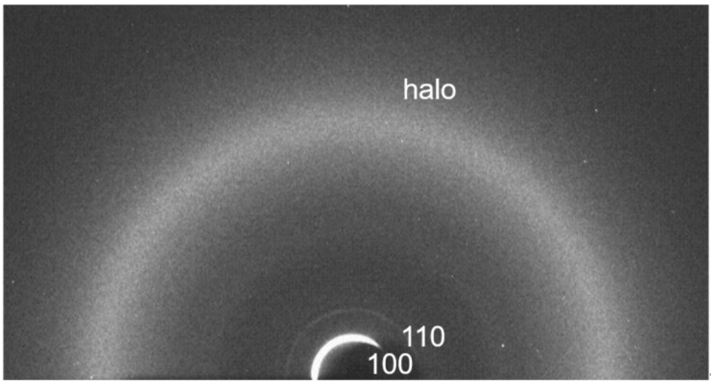

b)

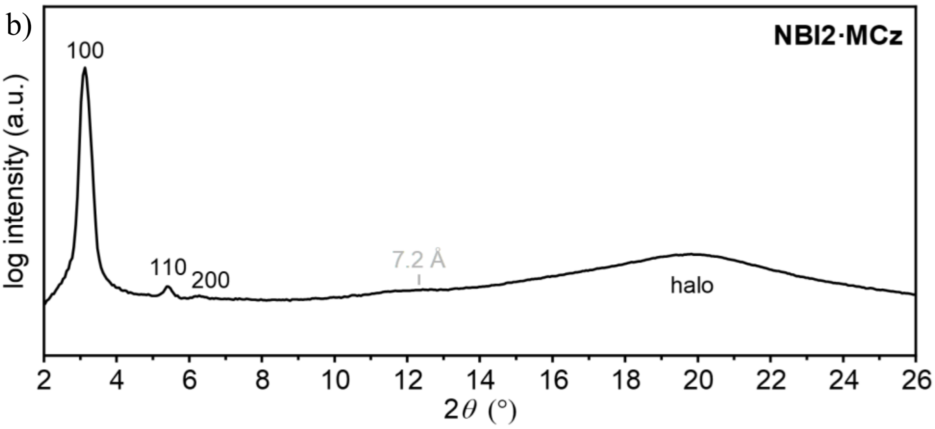

Figure S38. WAXS pattern of a) unaligned **NBI2·MCz** (5:1) at 298 K and b) integrated intensities. The peak on the meridian of the aligned host **NBI2** assigned to intracolumnar order is shown in gray. Random spots in the pattern (zingers) are sudden, localized energy-deposition events that appear as isolated, spurious signals caused by high-energy radiation (e.g., cosmic rays or scattered X-rays) and are unrelated to the true measurement; upon intensity integration, these spots disappear.[S9]

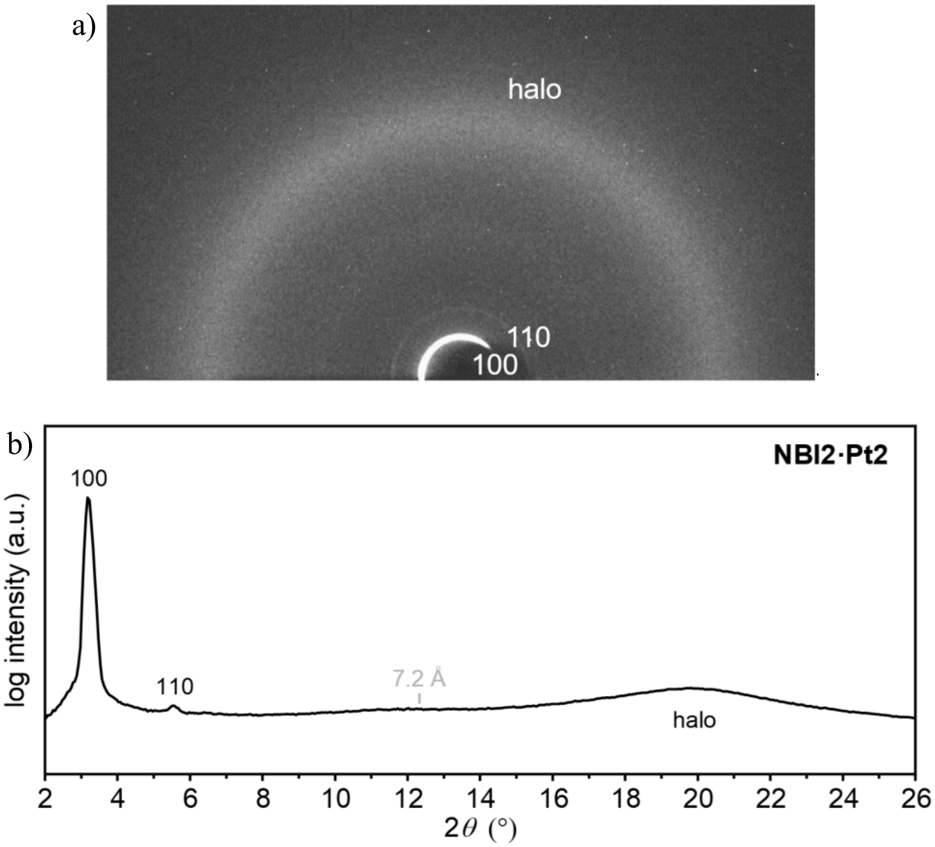

Figure S39. WAXS pattern of a) unaligned **NBI2·Pt2** (5:1) at 298 K and b) integrated intensities. The peak on the meridian of the aligned host **NBI2** assigned to intracolumnar order is shown in gray. Random spots in the pattern (zingers) are sudden, localized energy-deposition events that appear as isolated, spurious signals caused by high-energy radiation (e.g., cosmic rays or scattered X-rays) and are unrelated to the true measurement; upon intensity integration, these spots disappear.[S9]

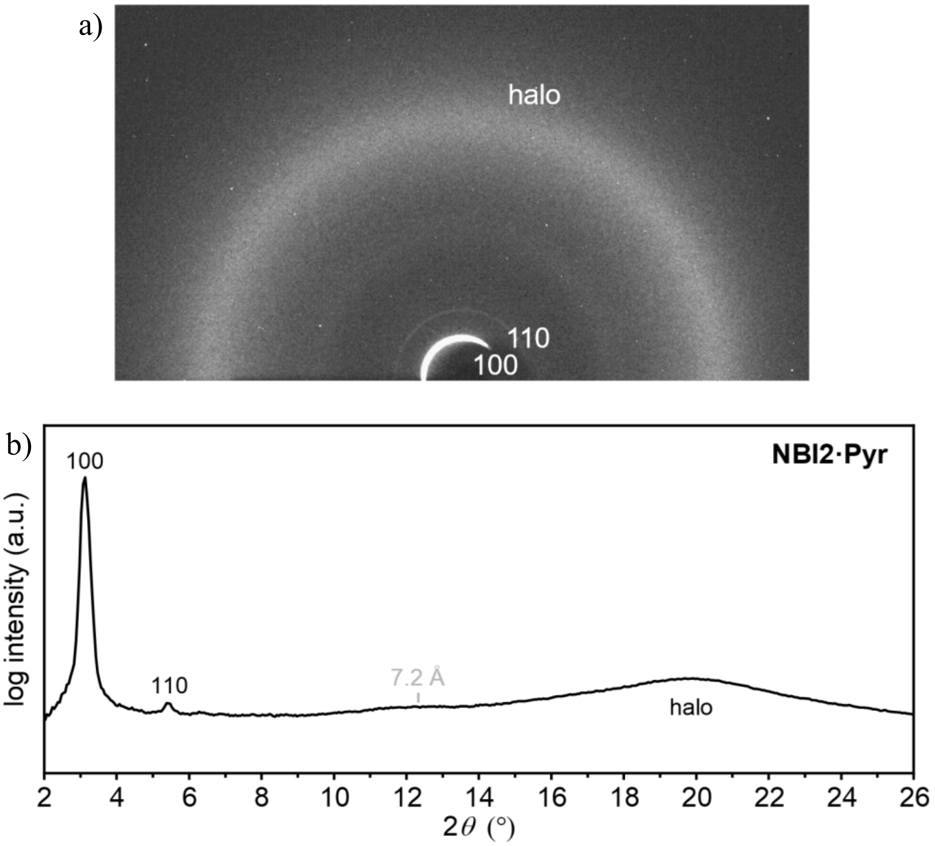

Figure S40. WAXS pattern of a) unaligned **NBI2·Pyr** (5:1) at 298 K and b) integrated intensities. The peak on the meridian of the aligned host **NBI2** assigned to intracolumnar order is shown in gray. Random spots in the pattern (zingers) are sudden, localized energy-deposition events that appear as isolated, spurious signals caused by high-energy radiation (e.g., cosmic rays or scattered X-rays) and are unrelated to the true measurement; upon intensity integration, these spots disappear.[S9]

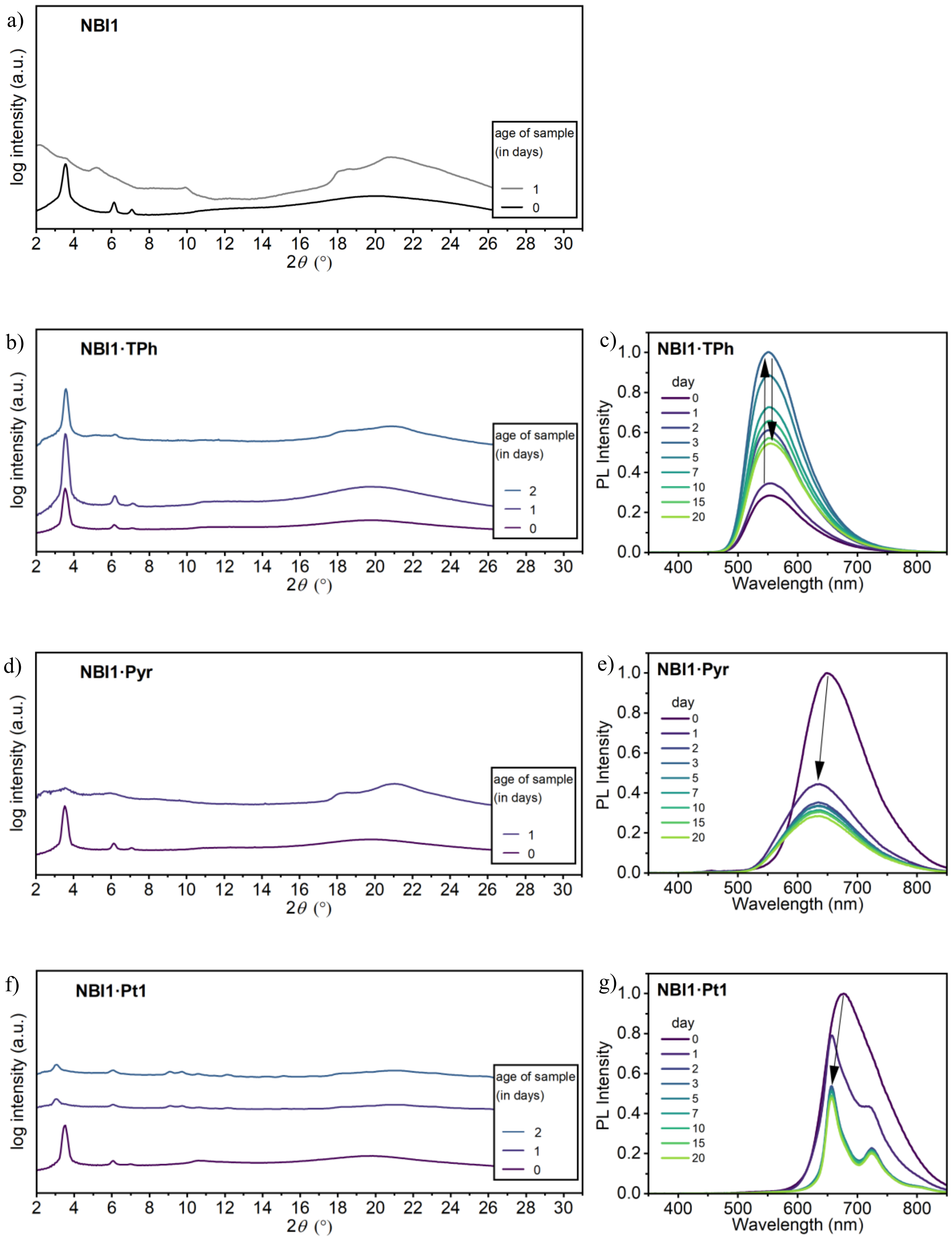

Figure S41. Stability and photoluminescence (PL) performance studies of the LC host–guest systems with WAXS and spectroscopic measurements over twenty days. Integrated intensities of the WAXS measurement of a) **NBI1**, b) **NBI1·TPh**, d) **NBI1·Pyr**, d) **NBI1·Pt1** and emission spectra of c) **NBI1·TPh**, e) **NBI1·Pyr**, g) **NBI1·Pt1**.

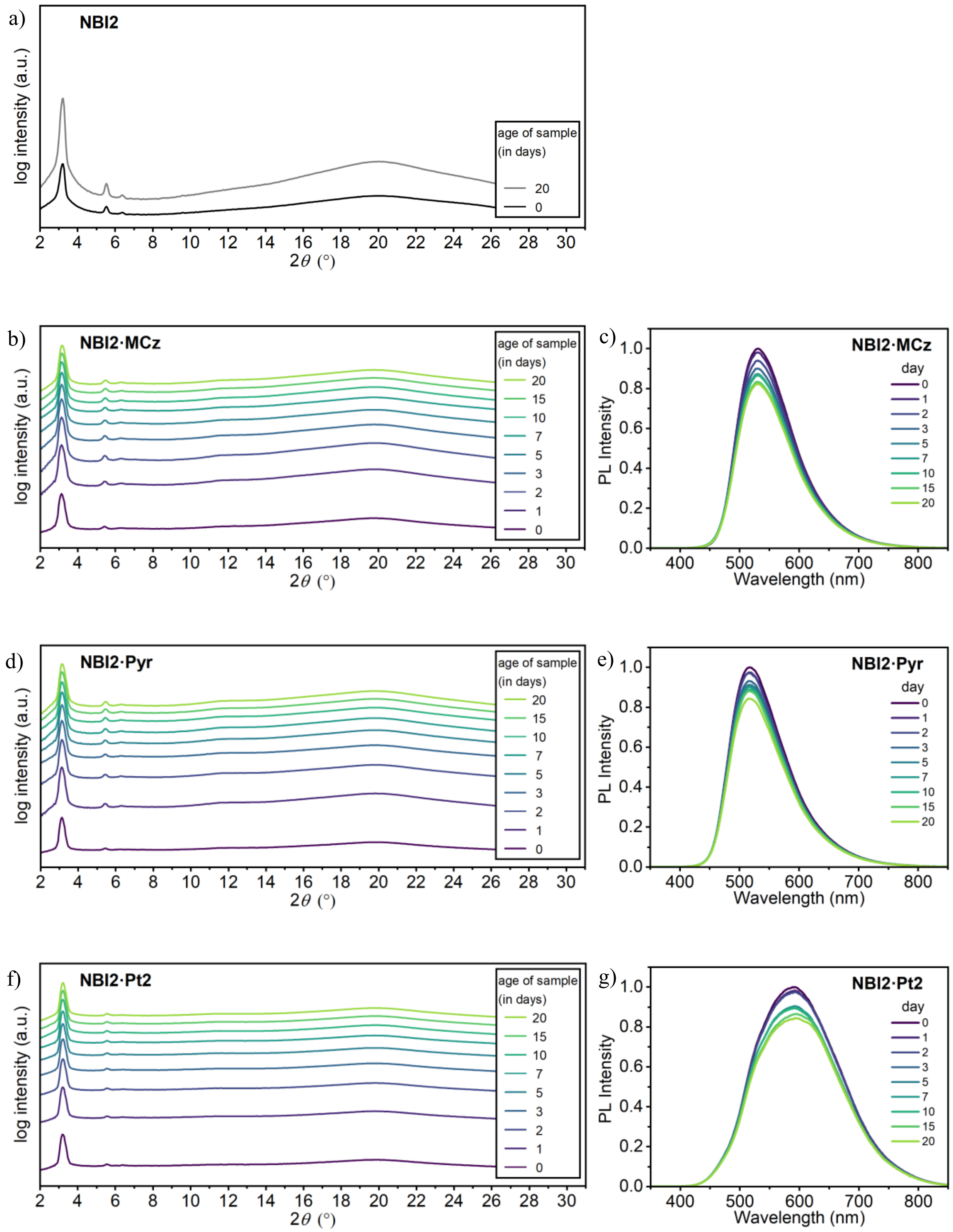

Figure S42. Stability and photoluminescence (PL) performance studies of the LC host–guest systems with WAXS and spectroscopic measurements over twenty days. Integrated intensities of the WAXS measurement of a) **NBI2**, b) **NBI2·MCz**, d) **NBI2·Pyr**, d) **NBI2·Pt2** and emission spectra of c) **NBI2·MCz**, e) **NBI2·Pyr**, g) **NBI2·Pt2**.

Table S5. Stability and PL performance of the LC host–guest systems, summarizing Col_h phase stability and changes in emission behavior over the time period of 20 days.

Host–guest mixtures	Stability of Col_h phase [in days]	PL	
		emission changed	Intensity
**NBI1**	< 1		
**NBI1·TPh**	1	no	fluctuation
**NBI1·Pyr**	< 1	yes	decreased
**NBI1·Pt1**	< 1	yes	decreased
**NBI2**	20		
**NBI2·MCz**	20	no	slight decrease
**NBI2·Pyr**	20	no	slight decrease
**NBI2·Pt2**	20	no	slight decrease
**(NBI2:NBI1)·Pyr** (100:1)	20	no	slight decrease
**(NBI2:NBI1)·Pyr** (100:5)	20	no	slight decrease
**(NBI2:NBI1)·Pyr** (100:10)	20	no	slight decrease
**(NBI2:NBI1)·Pyr** (100:100)	< 5	no	slight decrease

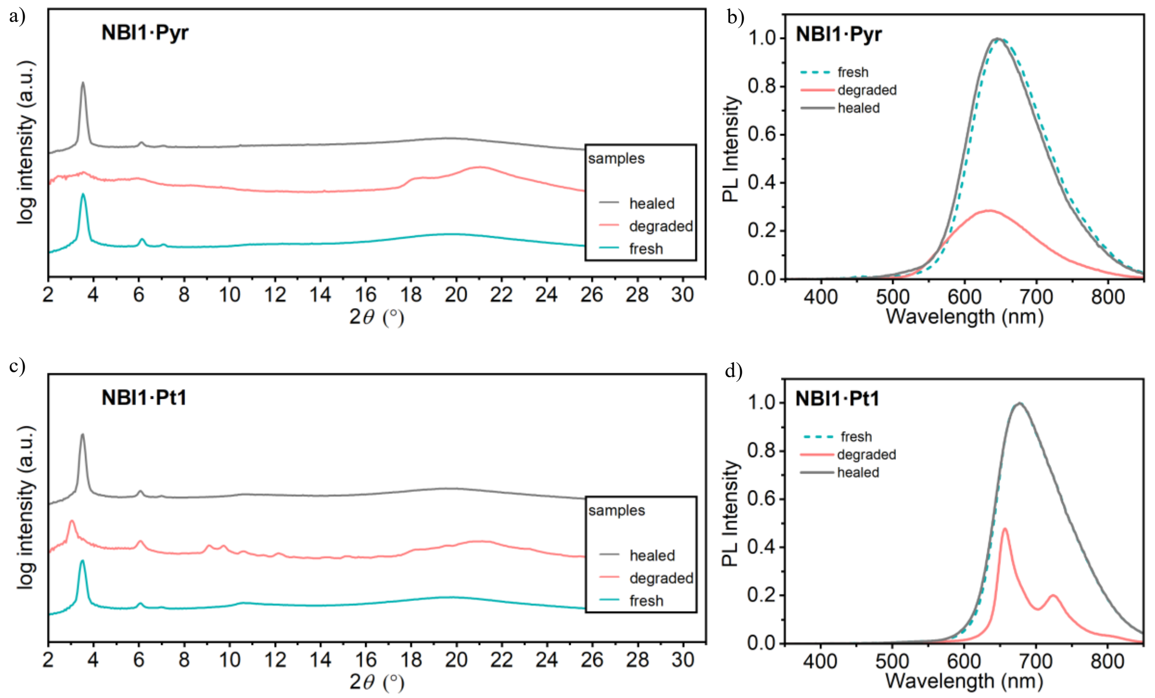

Figure S43. Stability and photoluminescence (PL) performance studies of the mixtures **NBI1·Pyr** and **NBI1·Pt1** with WAXS and spectroscopic measurements. Integrated intensities of the WAXS measurement of a) **NBI1·Pyr** and c) **NBI1·Pt1** and emission spectra of the fresh, degraded as well as regenerated sample of b) **NBI1·Pyr** and d) **NBI1·Pt1**.

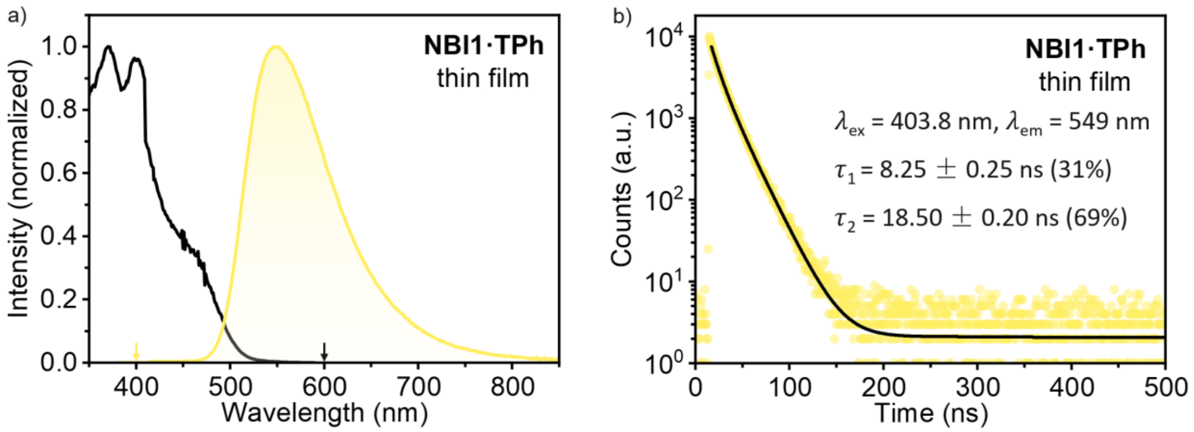

Figure S44. PL measurements of the LC mixture **NBI1·TPh** (5:1) as thin film at room temperature on quartz substrate. a) Normalized excitation (black line) and emission spectrum (in the color of emission, λ_{em} = 600 nm, λ_{ex} = 400 nm). The arrows indicate λ_{em} of the excitation spectrum (black) and λ_{ex} of the emission spectrum (colored). b) PL lifetime decay (symbol) detected at the maximum of emission with the best fit (black line). The experimental conditions for lifetime measurements λ_{ex} and λ_{em} and the lifetime components of the decay are given next to the graph.

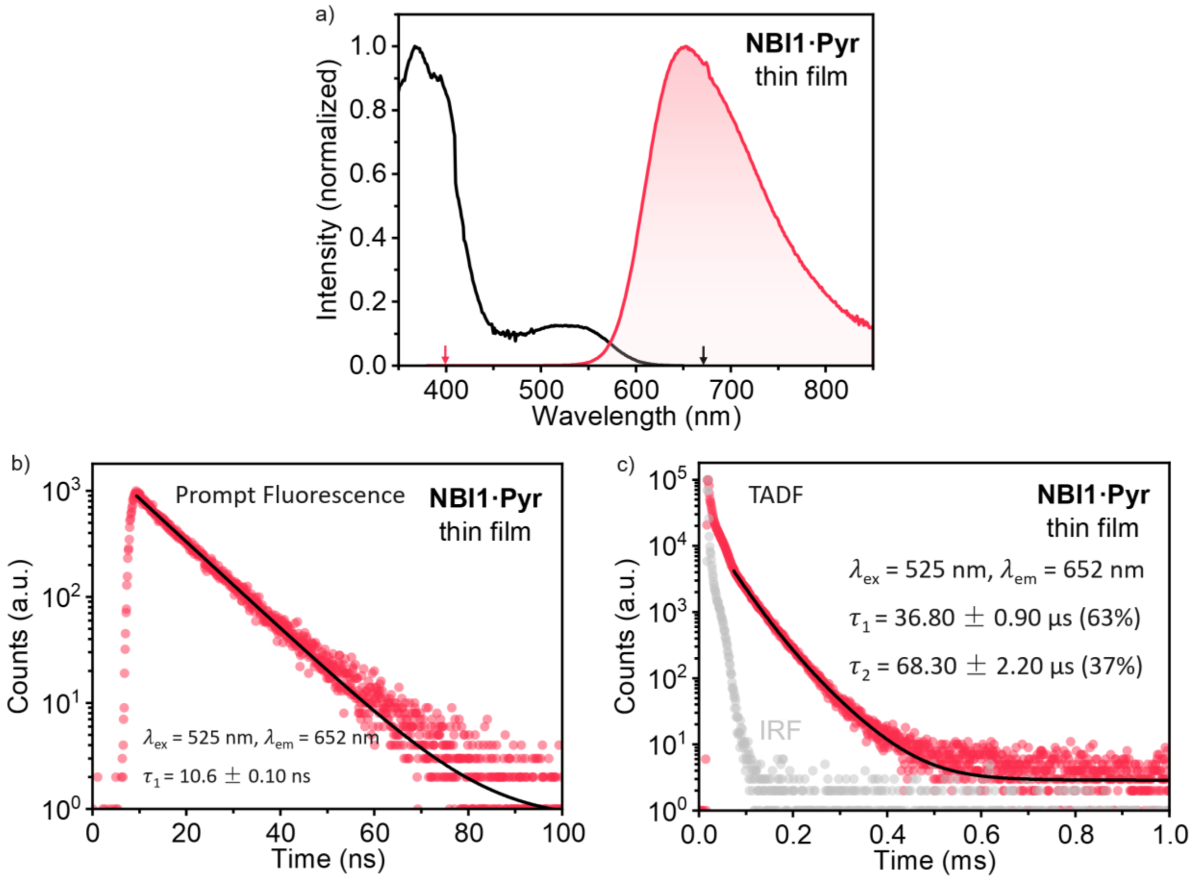

Figure S45. PL measurements of the LC mixture **NBI1·Pyr** (5:1) as thin film at room temperature on quartz substrate. a) Normalized excitation (black line) and emission spectrum (in the color of emission, λ_{em} = 670 nm, λ_{ex} = 400 nm). The arrows indicate λ_{em} of the excitation spectrum (black) and λ_{ex} of the emission spectrum (colored). b),c) PL lifetime decay (symbol) detected at the maximum of emission (the instrument response function, IRF is shown in gray) with the best fit (black line). The experimental conditions for lifetime measurements λ_{ex} and λ_{em} and the lifetime components of the decay are given next to the graph.

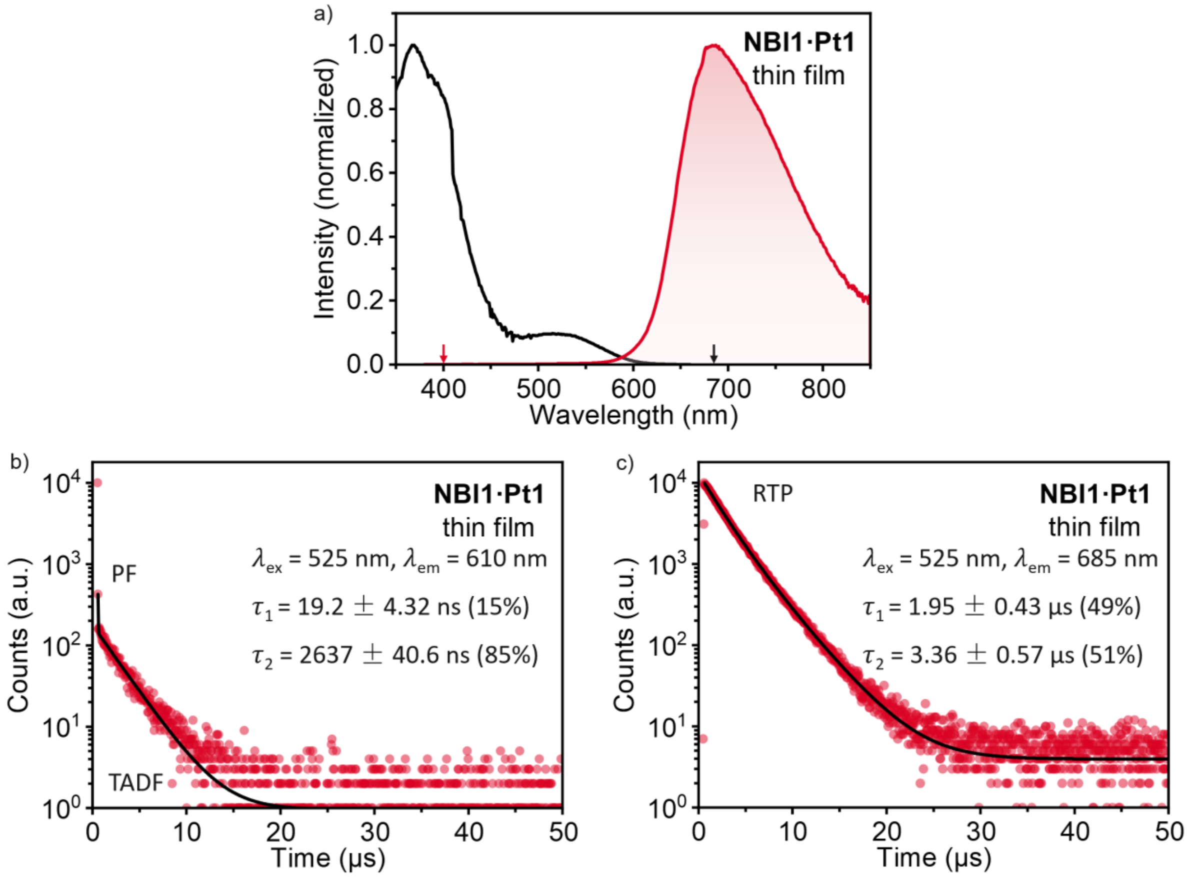

Figure S46. PL measurements of the LC mixture **NBI1·Pt1** (5:1) as thin film at room temperature on quartz substrate. a) Normalized excitation (black line) and emission spectrum (in the color of emission, λ_{em} = 685 nm, λ_{ex} = 400 nm). The arrows indicate λ_{em} of the excitation spectrum (black) and λ_{ex} of the emission spectrum (colored). b),c) PL lifetime decay (symbol) with the best fit (black line). The experimental conditions for lifetime measurements λ_{ex} and λ_{em} and the lifetime components of the decay are given next to the graph.

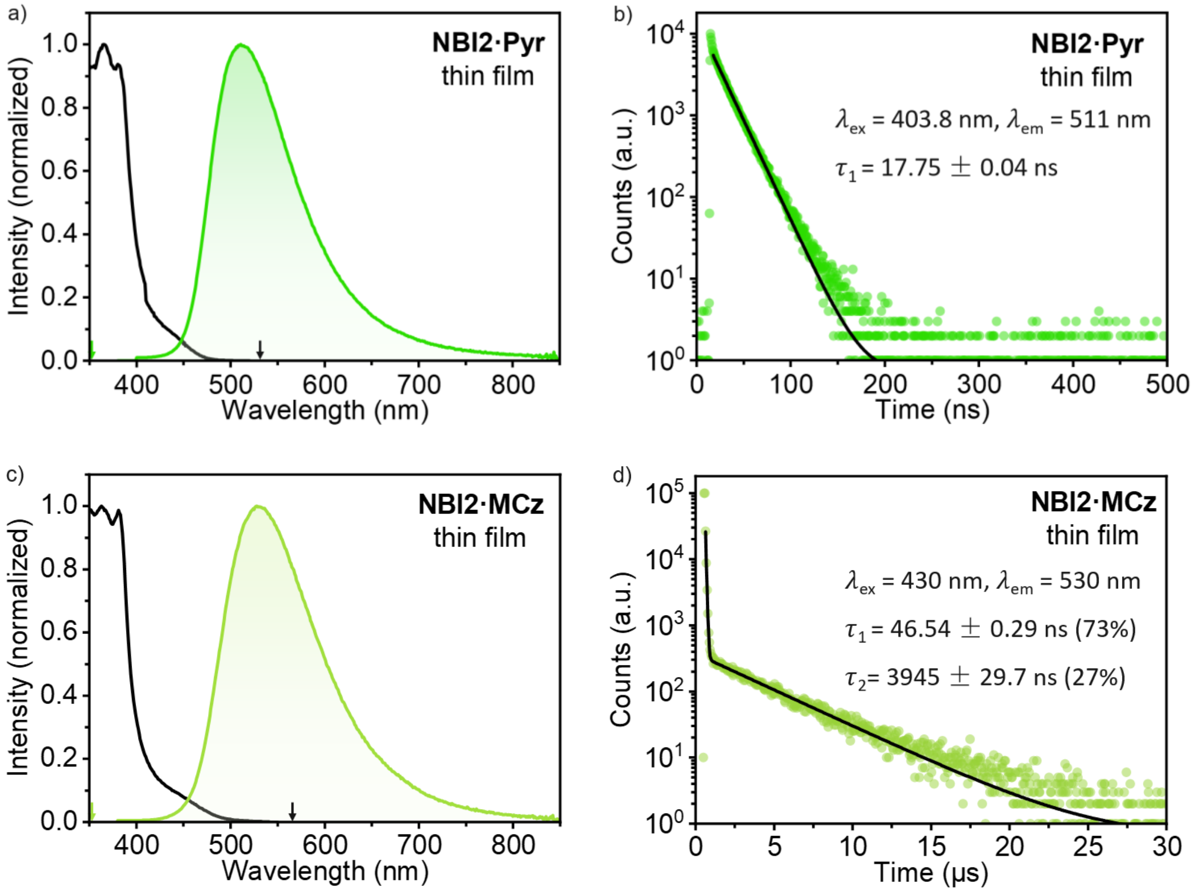

Figure S47. PL measurements the **NBI2** LC mixtures (5:1) **NBI2·Pyr** (a, b), **NBI2·MCz** (c, d) and as thin films at room temperature on quartz substrates. Normalized excitation (black line) and emission spectrum (in the color of emission) of a) **NBI2·Pyr** (λ_{em} = 530 nm, λ_{ex} = 350 nm), c) **NBI2·MCz** (λ_{em} = 560 nm, λ_{ex} = 350 nm). The arrows indicate λ_{em} of the excitation spectrum (black) and λ_{ex} of the emission spectrum (colored). PL lifetime decay (symbol) of b) **NBI2·Pyr**, d) **NBI2·MCz** detected at the maximum of emission with the best fit (black line). The experimental conditions for lifetime measurements λ_{ex} and λ_{em} and the lifetime components of the decay are given next to the graph.

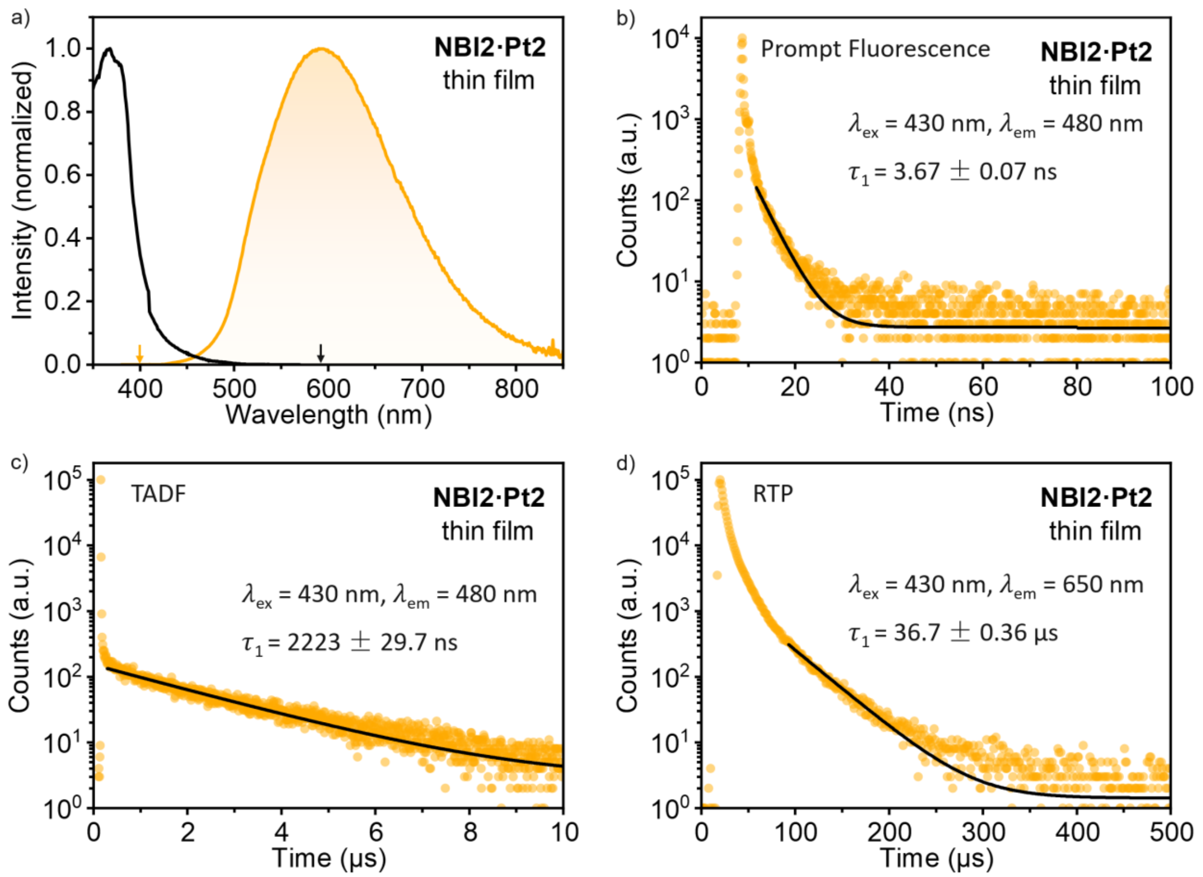

Figure S48. PL measurements of the LC mixture **NBI2·Pt2** (5:1) as thin film at room temperature on quartz substrate. a) Normalized excitation (black line) and emission spectrum (in the color of emission, λ_{em} = 593 nm, λ_{ex} = 400 nm). The arrows indicate λ_{em} of the excitation spectrum (black) and λ_{ex} of the emission spectrum (colored). b),c),d) PL lifetime decay (symbol) with the best fit (black line). The experimental conditions for lifetime measurements λ_{ex} and λ_{em} and the lifetime components of the decay are given next to the graph.

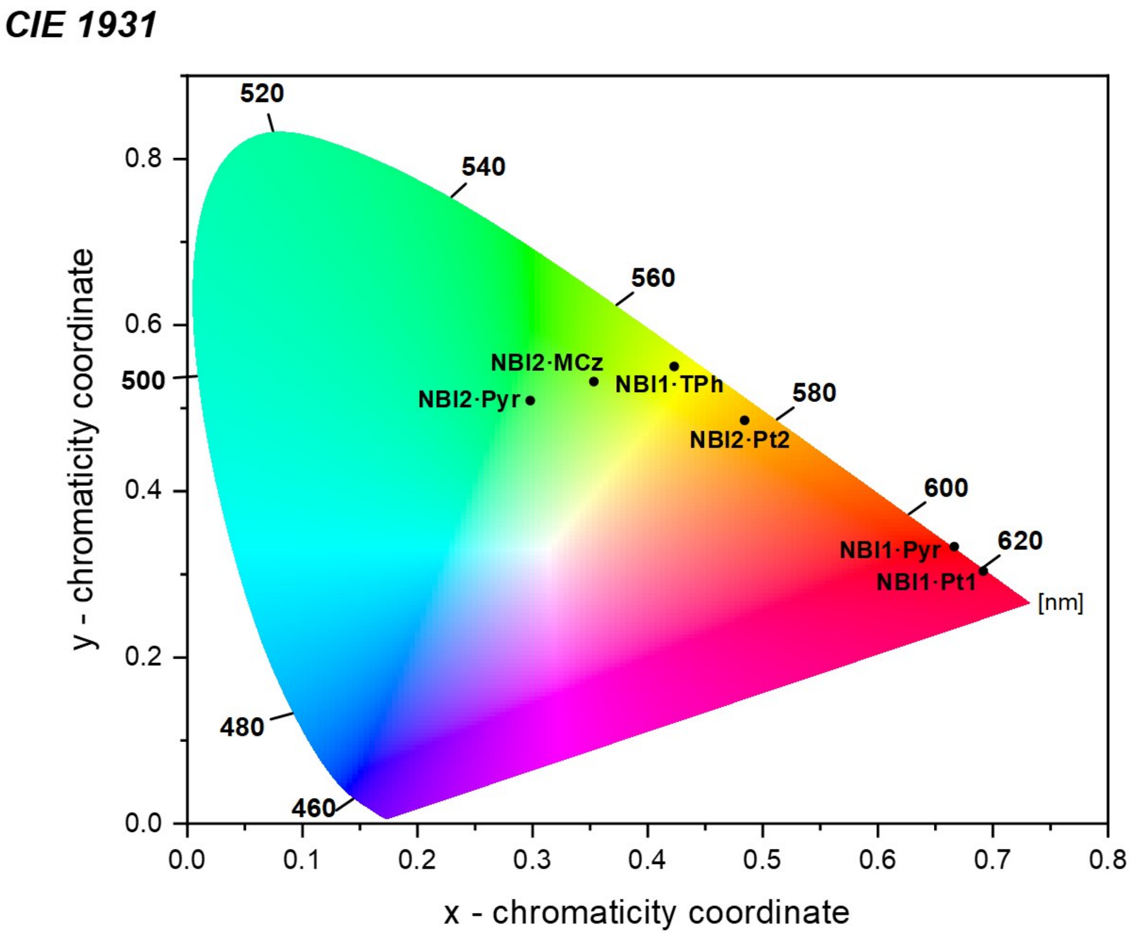

Figure S49. Luminescence of the LC mixtures (5:1) **NBI2·Pyr**, **NBI2·MCz**, **NBI1·TPh**, **NBI2·Pt2**, **NBI1·Pyr** and **NBI2·Pt1** in the CIE 1931 (x,y) diagram with a colorimetric representation of the human visible color space, showing the spectral colors from violet to red along the arc in nm.

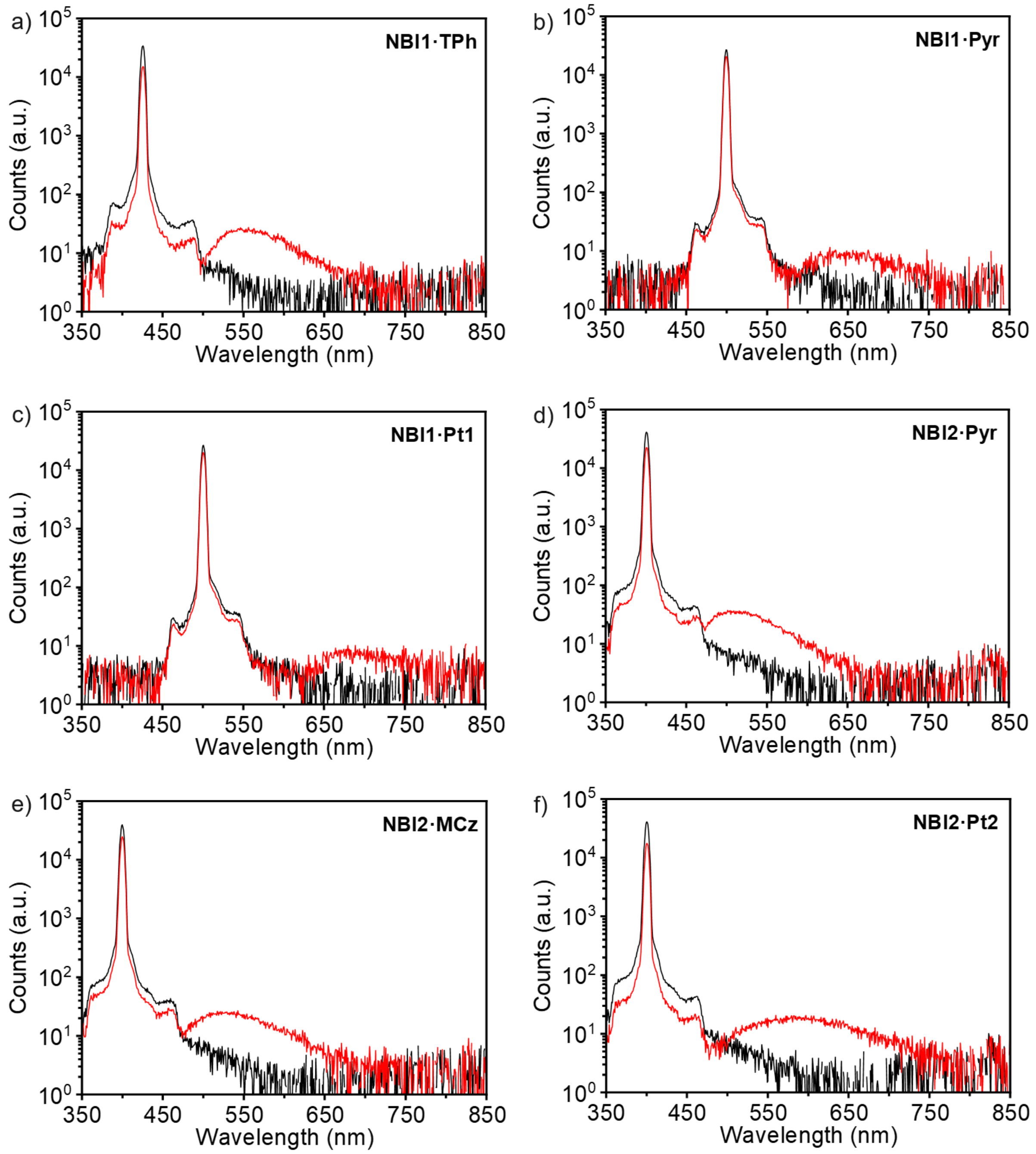

Figure S50. Optical profiles of the integrating sphere measurements of reference (black line) and the LC mixtures (5:1) (red line) of a) **NBI1·TPh** (λ_{ex} = 425 nm), b) **NBI1·Pyr** (λ_{ex} = 500 nm), c) **NBI1·Pt1** (λ_{ex} = 500 nm), d) **NBI2·Pyr** (λ_{ex} = 400 nm), e) **NBI2·MCz** (λ_{ex} = 400 nm) and f) **NBI1·Pt2** (λ_{ex} = 400 nm) at ambient conditions.

Table S6. Temperature-dependent Φ_{PL} of liquid crystal host–guest mixture.

Material	λ_{em} [nm]	Φ_{PL} [%] 298 K	Φ_{PL} [%] 220 K	Φ_{PL} [%] 150 K	Φ_{PL} [%] 80 K	PL
**NBI2·MCz**	530	4	11	17	20	TADF
**NBI2·Pt2**	593	3	14	34	46	TADF+RTP
**NBI1·Pyr**	652	3	4.5[a)]	5.2[b)]	6.3	TADF
**NBI1·Pt1**	685	4	7	9	10	TADF+RTP

Φ_{PL} at different temperatures calculated from the area of the PL emission curve and Φ_{PL} at 298 K from measurement. [a)] measured at 110 K, [b)] measured at 230 K.

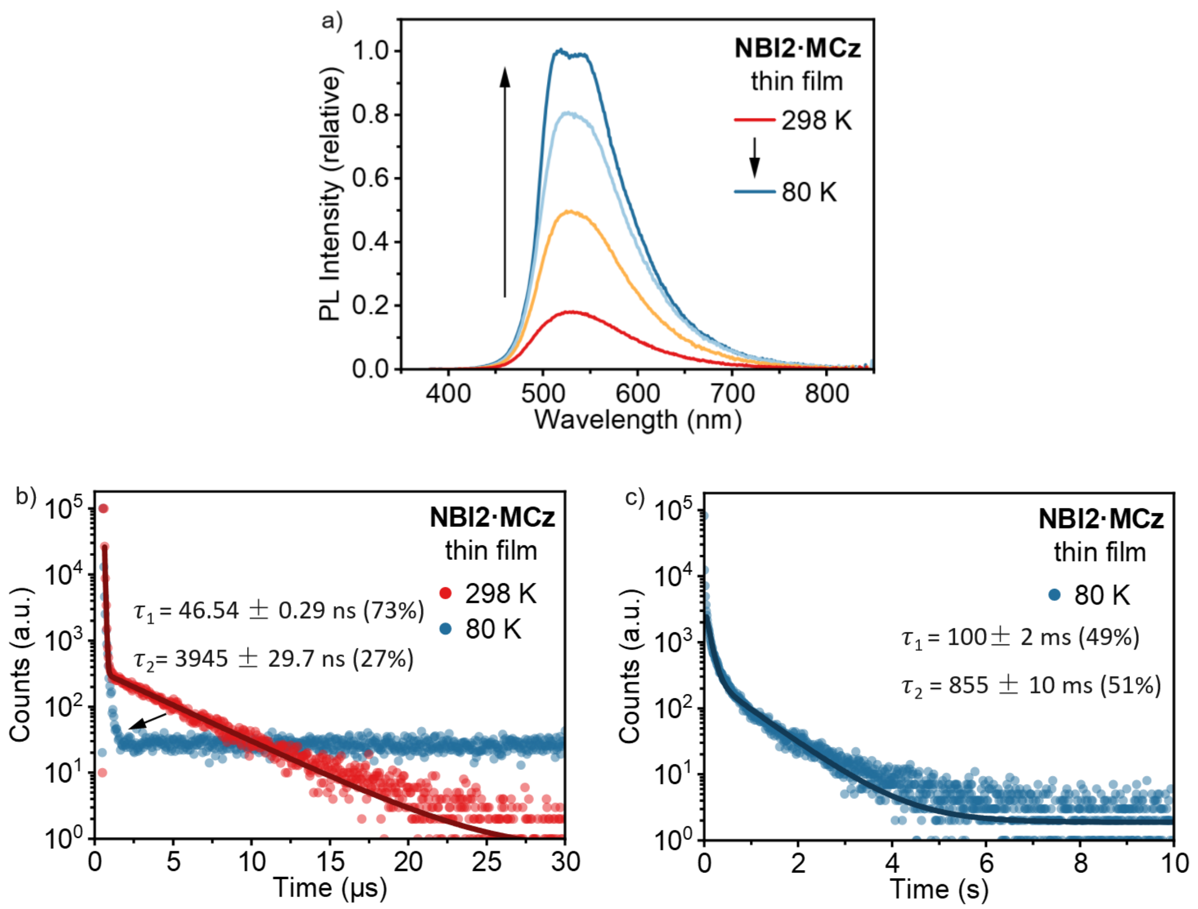

Figure S51. Temperature-dependent PL measurements of the LC mixture **NBI2·MCz** (5:1). a) Relative PL intensities displayed at 80 K (dark blue), 150 K (light blue), 220 K (orange) and 298 K (red) with λ_{ex} = 350 nm. PL lifetime decay b) at 298 K (symbol, red) and 80 K (symbol, blue) with λ_{ex} = 430 nm, λ_{em} = 530 nm and c) 80 K (symbol, blue) with λ_{ex} = 430 nm, λ_{em} = 518 nm and the best fit (dark red and dark blue line). The lifetime components of the decay are given next to the graph.

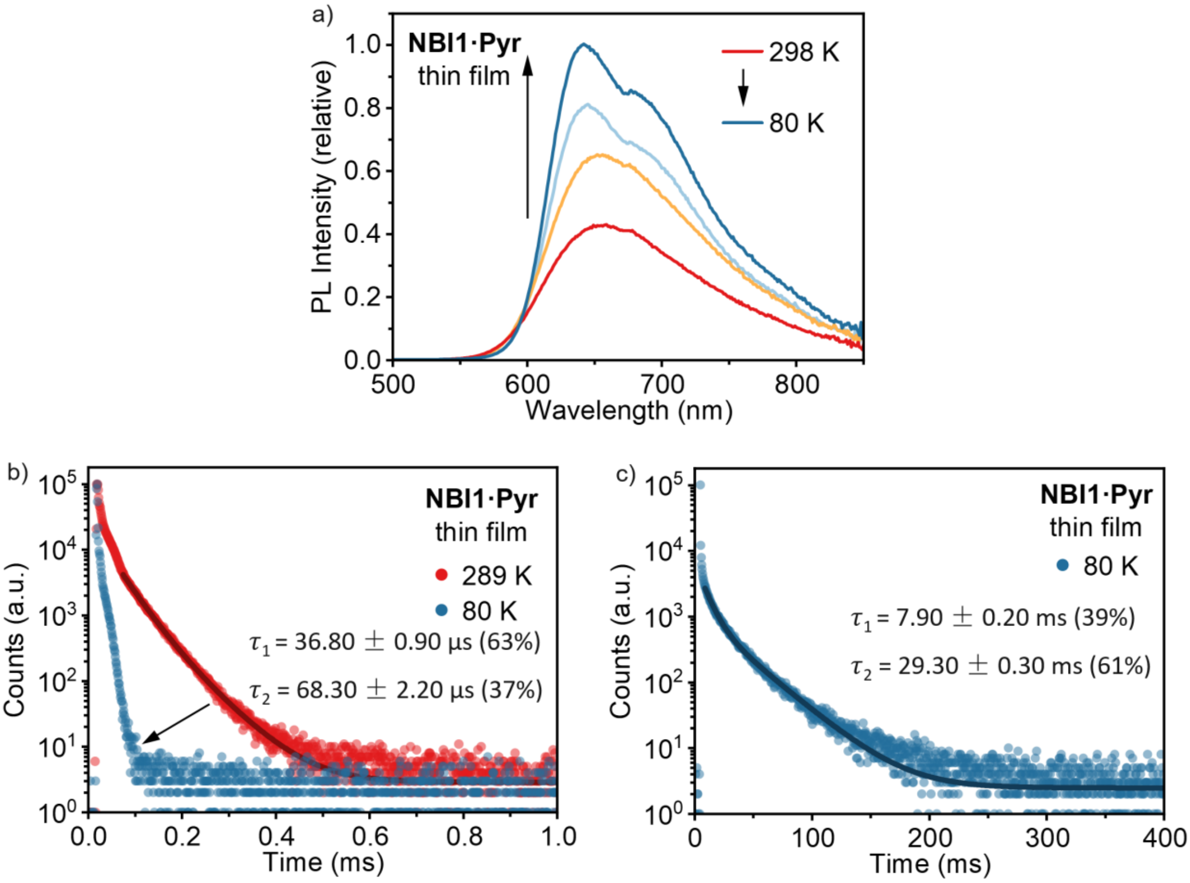

Figure S52. Temperature-dependent PL measurements of the LC mixture **NBI1·Pyr** (5:1). a) Relative PL intensities displayed at 80 K (dark blue), 110 K (light blue), 230 K (orange) and 298 K (red) with λ_{ex} = 400 nm. PL lifetime decay b) at 298 K (symbol, red) and 80 K (symbol, blue) with λ_{ex} = 525 nm, λ_{em} = 650 nm and c) 80 K (symbol, blue) with λ_{ex} = 525 nm, λ_{em} = 650 nm and the best fit (dark red and dark blue line). The lifetime components of the decay are given next to the graph.

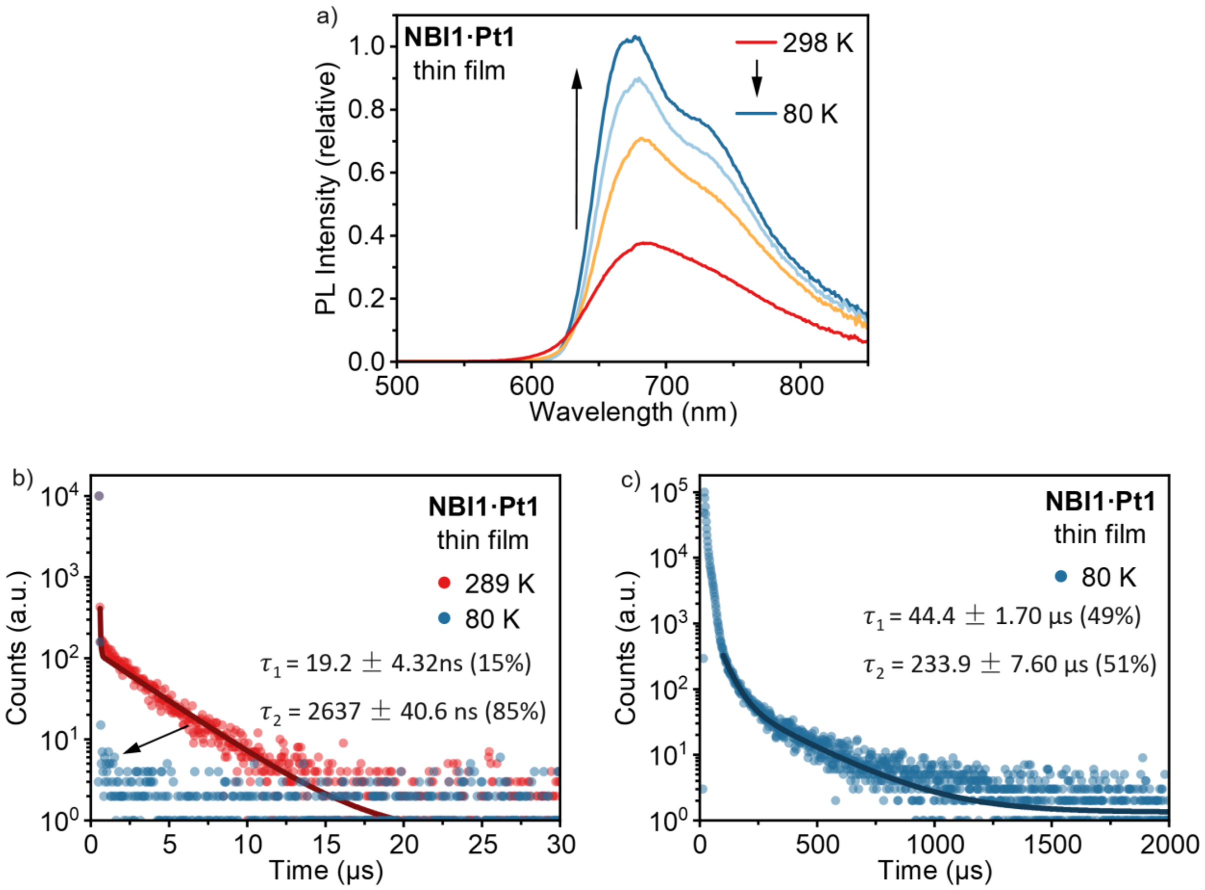

Figure S53. Temperature-dependent PL measurements of the LC mixture **NBI1·Pt1** (5:1). a) Relative PL intensities displayed at 80 K (dark blue), 150 K (light blue), 220 K (orange) and 298 K (red) with λ_{ex} = 400 nm. PL lifetime decay b) at 298 K (symbol, red) and 80 K (symbol, blue) with λ_{ex} = 525 nm, λ_{em} = 610 nm and c) 80 K (symbol, blue) with λ_{ex} = 525 nm, λ_{em} = 672 nm and the best fit (dark red and dark blue line). The lifetime components of the decay are given next to the graph.

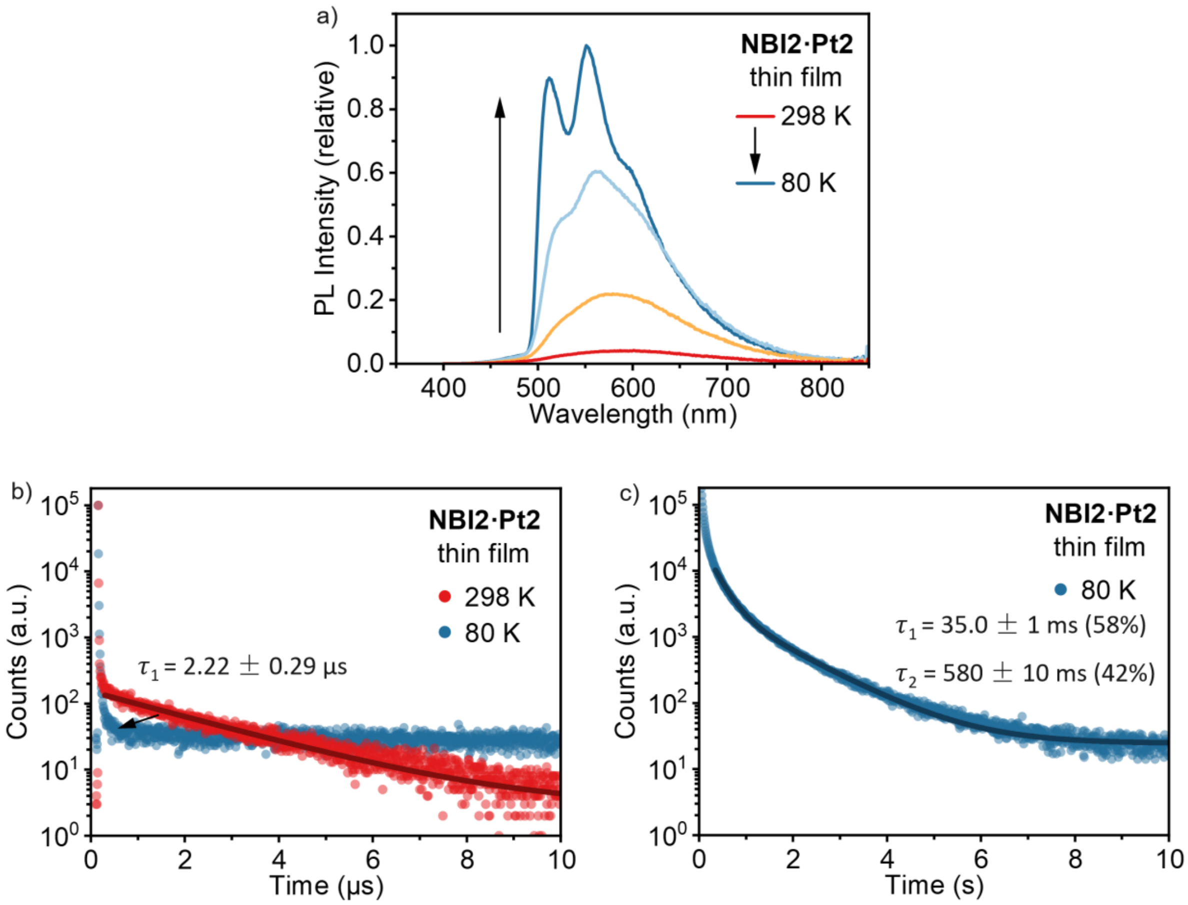

Figure S54. Temperature-dependent PL measurements of the LC mixture **NBI1·Pt2** (5:1). a) Relative PL intensities displayed at 80 K (dark blue), 150 K (light blue), 220 K (orange) and 298 K (red) with λ_{ex} = 400 nm. PL lifetime decay b) at 298 K (symbol, red) and 80 K (symbol, blue) with λ_{ex} = 430 nm, λ_{em} = 480 nm and c) 80 K (symbol, blue) with λ_{ex} = 400 nm, λ_{em} = 551 nm and the best fit (dark red and dark blue line). The lifetime components of the decay are given next to the graph.

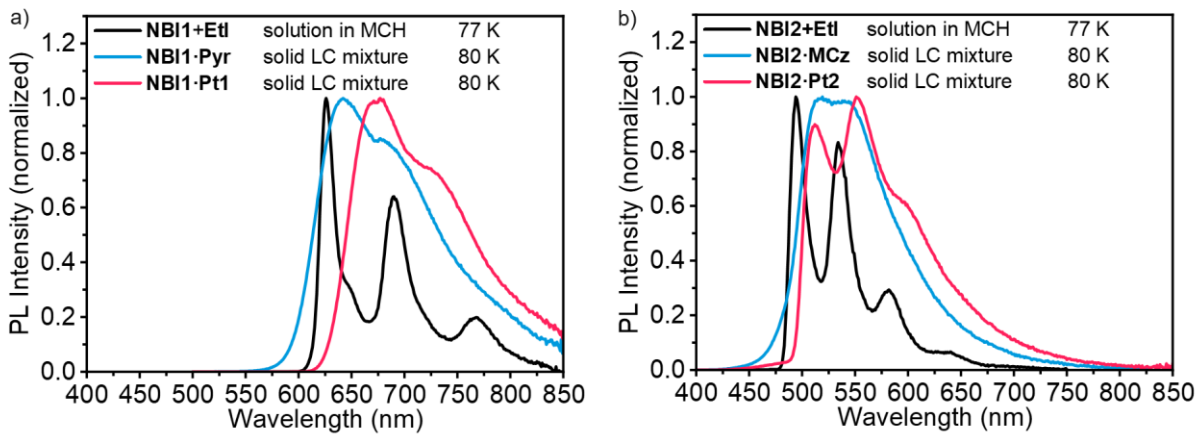

Figure S55. Low-temperature PL measurements of mixtures with triplet activation compared to the sensitization with ethyl iodide (**EtI**). a) **NBI1** with 2 mL **EtI** in MCH ($c_0 = 1 \times 10^{-5}$ M) at 77 K (black), **NBI1·Pyr** (blue) and **NBI1·Pt1** LC mixtures (5:1 ratio) as thin films at 80 K. b) a) **NBI2** with 2 mL **EtI** in MCH ($c_0 = 1 \times 10^{-5}$ M) at 77 K (black), **NBI2·MCz** (blue) and **NBI1·Pt2** LC mixtures (5:1 ratio) as thin films at 80 K.

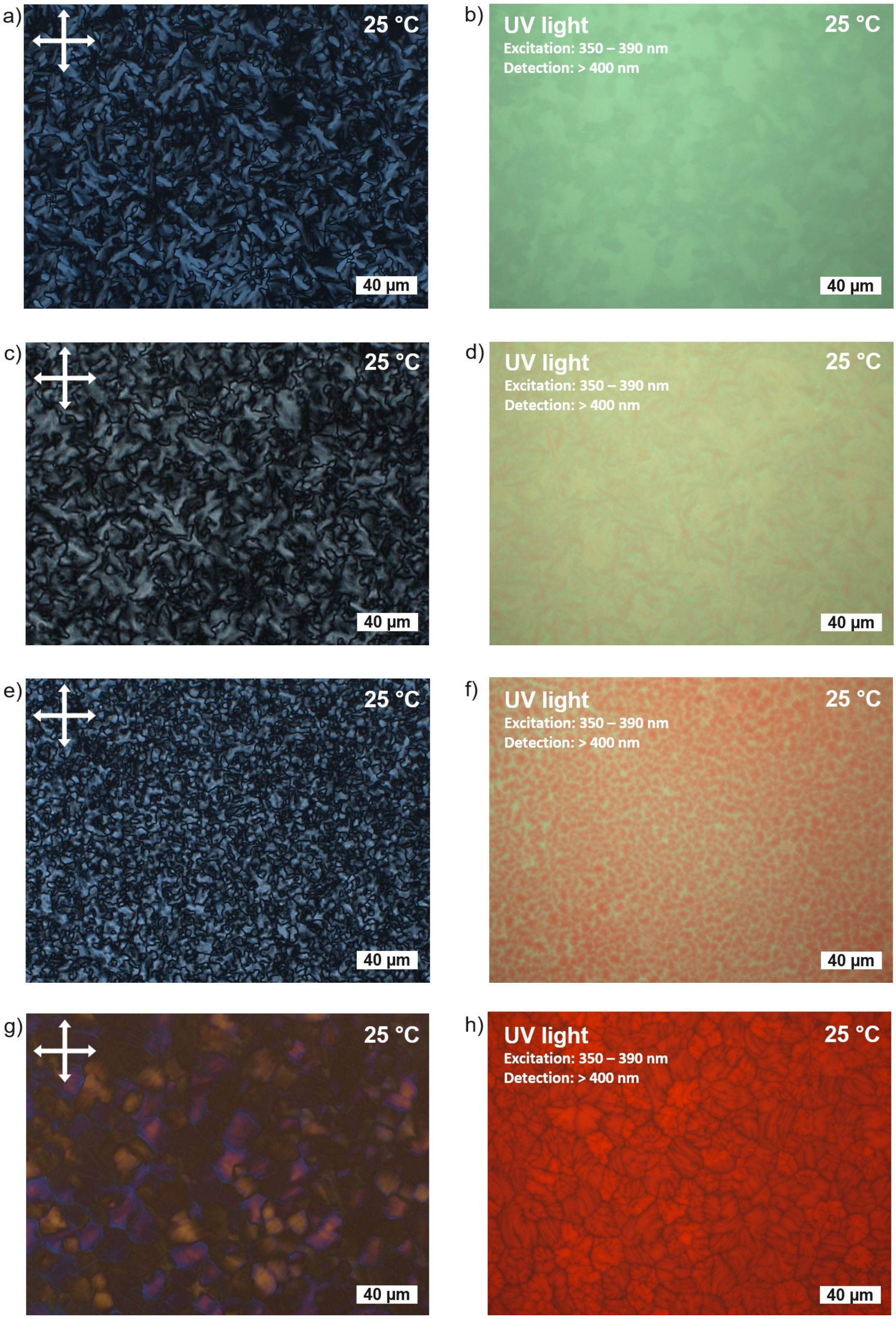

Figure S56. POM images of the mixtures **(NBI2:NBI1)·Pyr** (LC host in total:guest, 5:1) with the ratio of LC hosts (**NBI2**:**NBI1**) (a, b) 100:1, (c, d) 100:5, (e, f) 100:10 and (g, h) 100:100 at 25 °C (298 K) recorded in (a, c, e, g) transmission mode with crossed polarizers after heating to the isotropic liquid and (b, d, f, h) PL-POM image recorded in reflection mode under UV light irradiation.

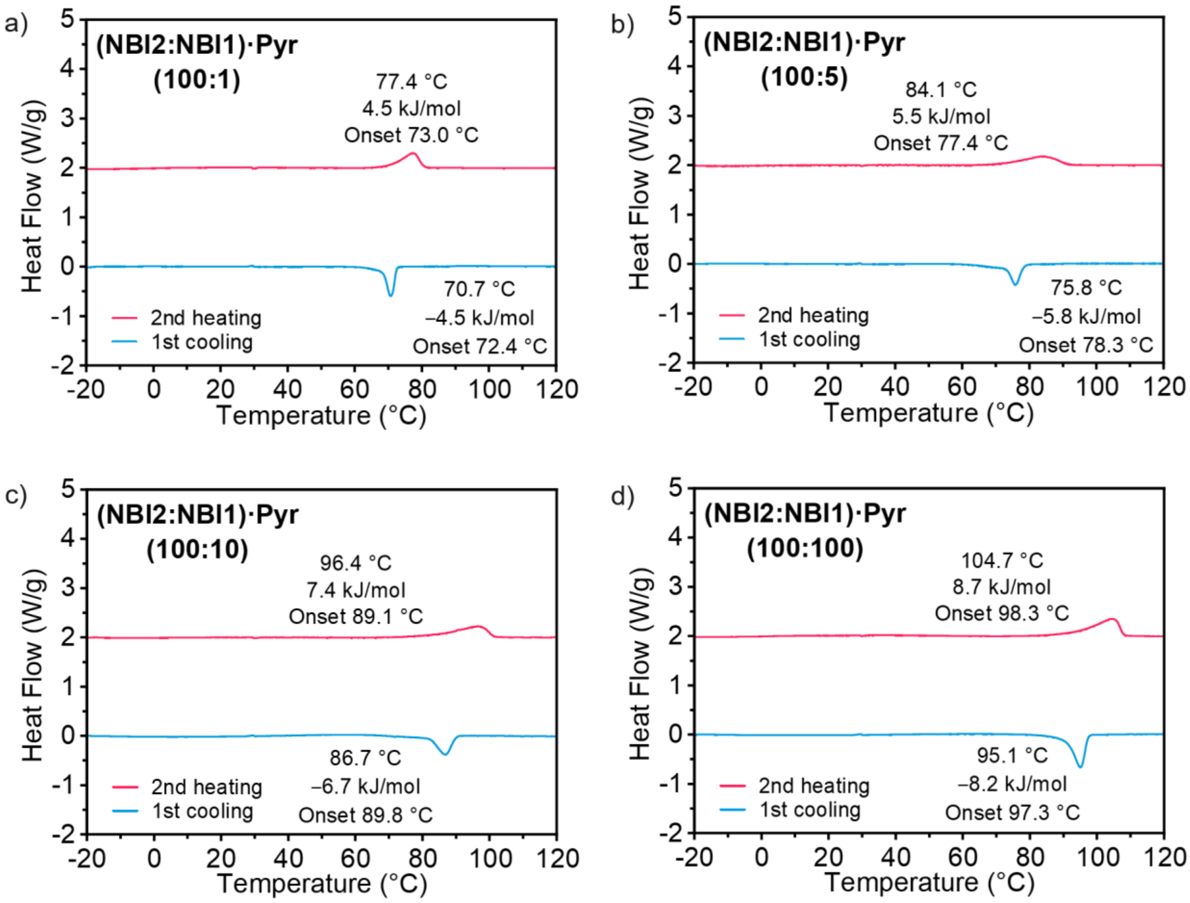

Figure S57. DSC traces of the mixtures **(NBI2:NBI1)·Pyr** (LC host in total:guest, 5:1) with the ratio of LC hosts (**NBI2**:**NBI1**) a) 100:1, b) 100:5, c) 100:10 and d) 100:100 from the second heating (red) and first cooling (blue) cycles with a rate of 10 K min^{-1}, endo up.

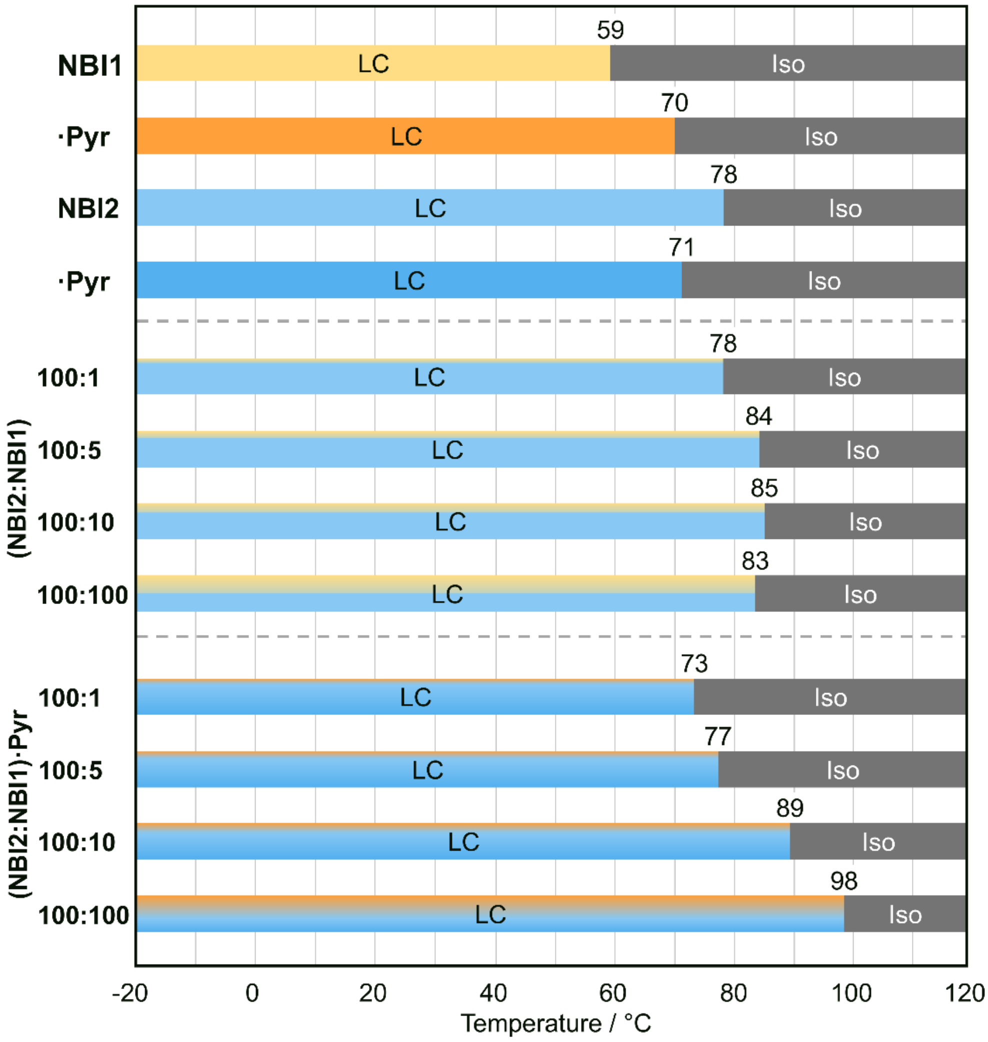

Figure S58. Temperature-dependent phase transition behavior of neat **NBI1** (yellow), neat **NBI2** (light blue) and mixtures with the guest **Pyr** (orange and dark blue), as well as mixtures of pure host LCs **NBI1:NBI2** and with **Pyr** (**(NBI2:NBI1)·Pyr**, LC host in total:guest, 5:1) with different mixing ratios of LC hosts (**NBI2**:**NBI1**) 100:1, 100:5, 100:10 and 100:100 (blue with orange), showing the clearing points upon heating; LC = liquid crystal; Iso = isotropic liquid.

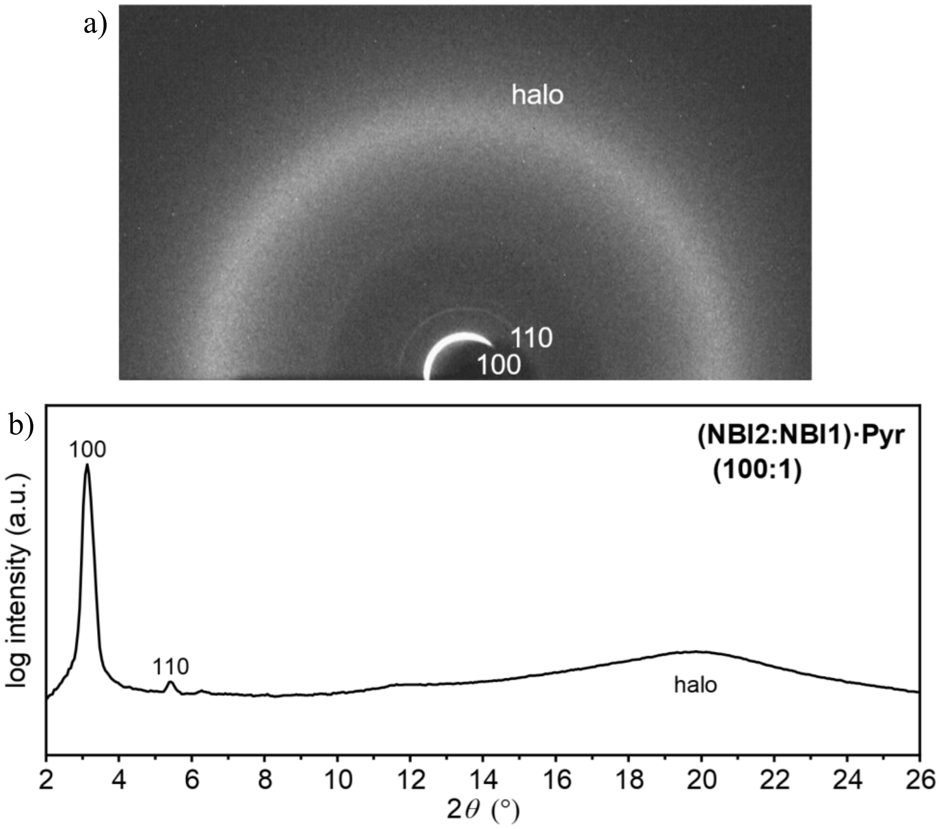

Figure S59. WAXS pattern of a) unaligned mixture **(NBI2:NBI1)·Pyr** (LC host:guest, 5:1) with the ratio of LC hosts (**NBI2**:**NBI1**) 100:1 at 298 K and b) integrated intensities. Random spots in the pattern (zingers) are sudden, localized energy-deposition events that appear as isolated, spurious signals caused by high-energy radiation (e.g., cosmic rays or scattered X-rays) and are unrelated to the true measurement; upon intensity integration, these spots disappear.[S9]

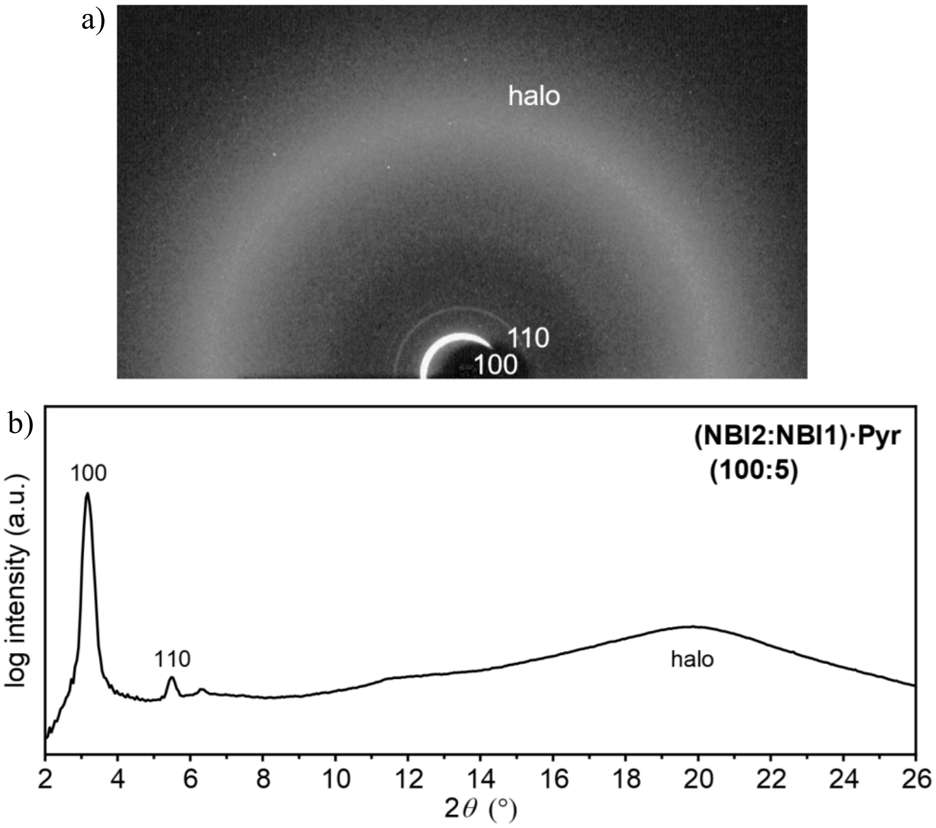

Figure S60. WAXS pattern of a) unaligned mixture **(NBI2:NBI1)·Pyr** (LC host:guest, 5:1) with the ratio of LC hosts (**NBI2**:**NBI1**) 100:5 at 298 K and b) integrated intensities. Random spots in the pattern (zingers) are sudden, localized energy-deposition events that appear as isolated, spurious signals caused by high-energy radiation (e.g., cosmic rays or scattered X-rays) and are unrelated to the true measurement; upon intensity integration, these spots disappear.[S9]

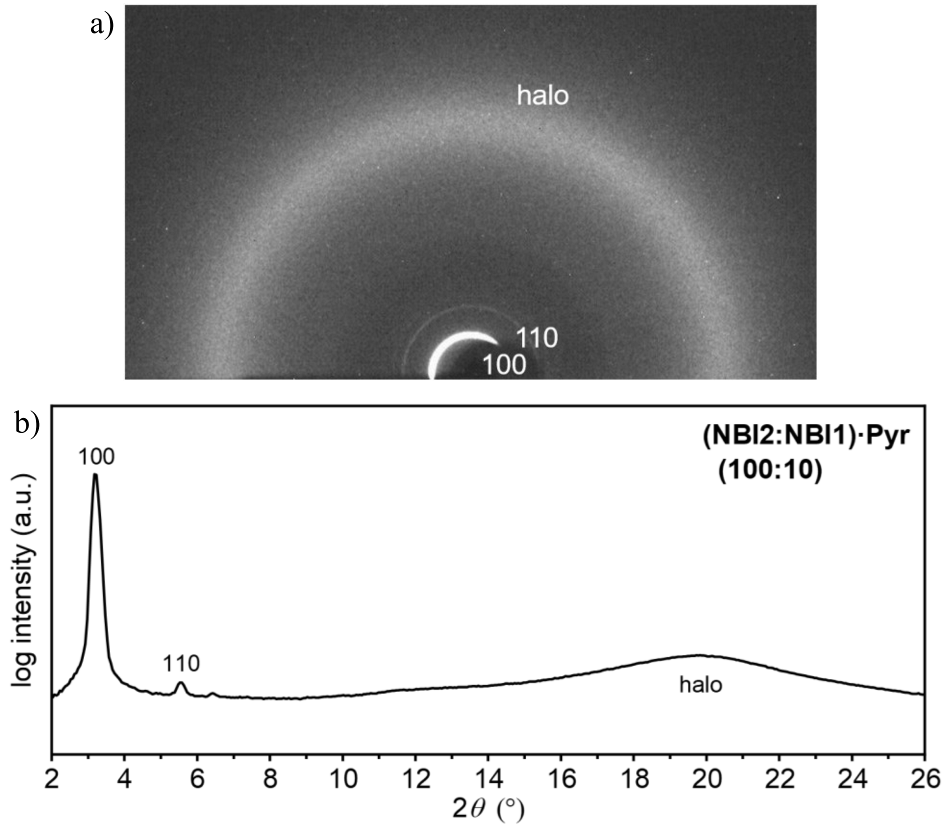

Figure S61. WAXS pattern of a) unaligned mixture **(NBI2:NBI1)·Pyr** (LC host:guest, 5:1) with the ratio of LC hosts (**NBI2**:**NBI1**) 100:10 at 298 K and b) integrated intensities. Random spots in the pattern (zingers) are sudden, localized energy-deposition events that appear as isolated, spurious signals caused by high-energy radiation (e.g., cosmic rays or scattered X-rays) and are unrelated to the true measurement; upon intensity integration, these spots disappear.[S9]

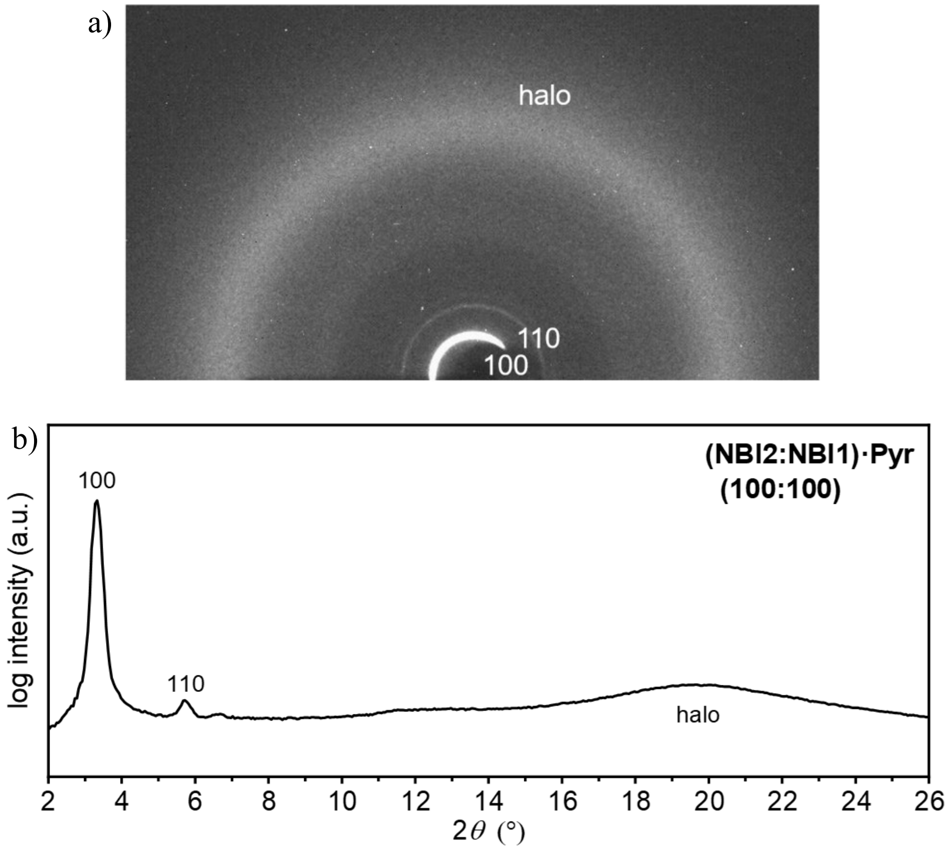

Figure S62. WAXS pattern of a) unaligned mixture **(NBI2:NBI1)·Pyr** (LC host:guest, 5:1) with the ratio of LC hosts (**NBI2**:**NBI1**) 100:100 at 298 K and b) integrated intensities. Random spots in the pattern (zingers) are sudden, localized energy-deposition events that appear as isolated, spurious signals caused by high-energy radiation (e.g., cosmic rays or scattered X-rays) and are unrelated to the true measurement; upon intensity integration, these spots disappear.[S9]

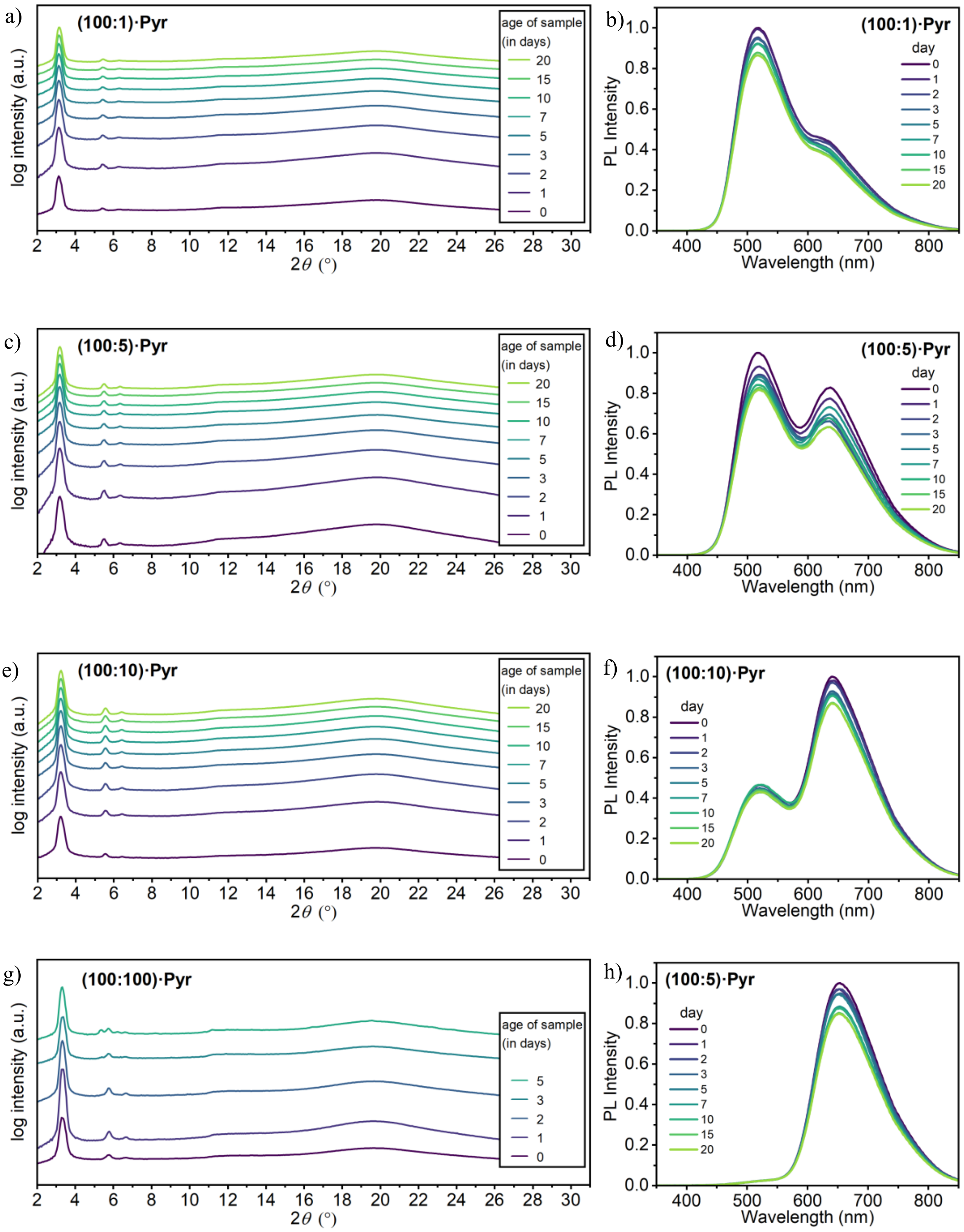

Figure S63. Stability and photoluminescence (PL) performance studies of the LC host–guest systems with WAXS and spectroscopic measurements over twenty days. Integrated intensities of the WAXS measurement of the host mixtures with **Pyr** (**(NBI2:NBI1)·Pyr**, LC host in total:guest, 5:1) with the ratio of LC hosts (**NBI2**:**NBI1**) a) 100:1, c) 100:5 , e) 100:10, g) 100:100 and emission spectra of b) 100:1, d) 100:5, f) 100:10, g) 100:100.

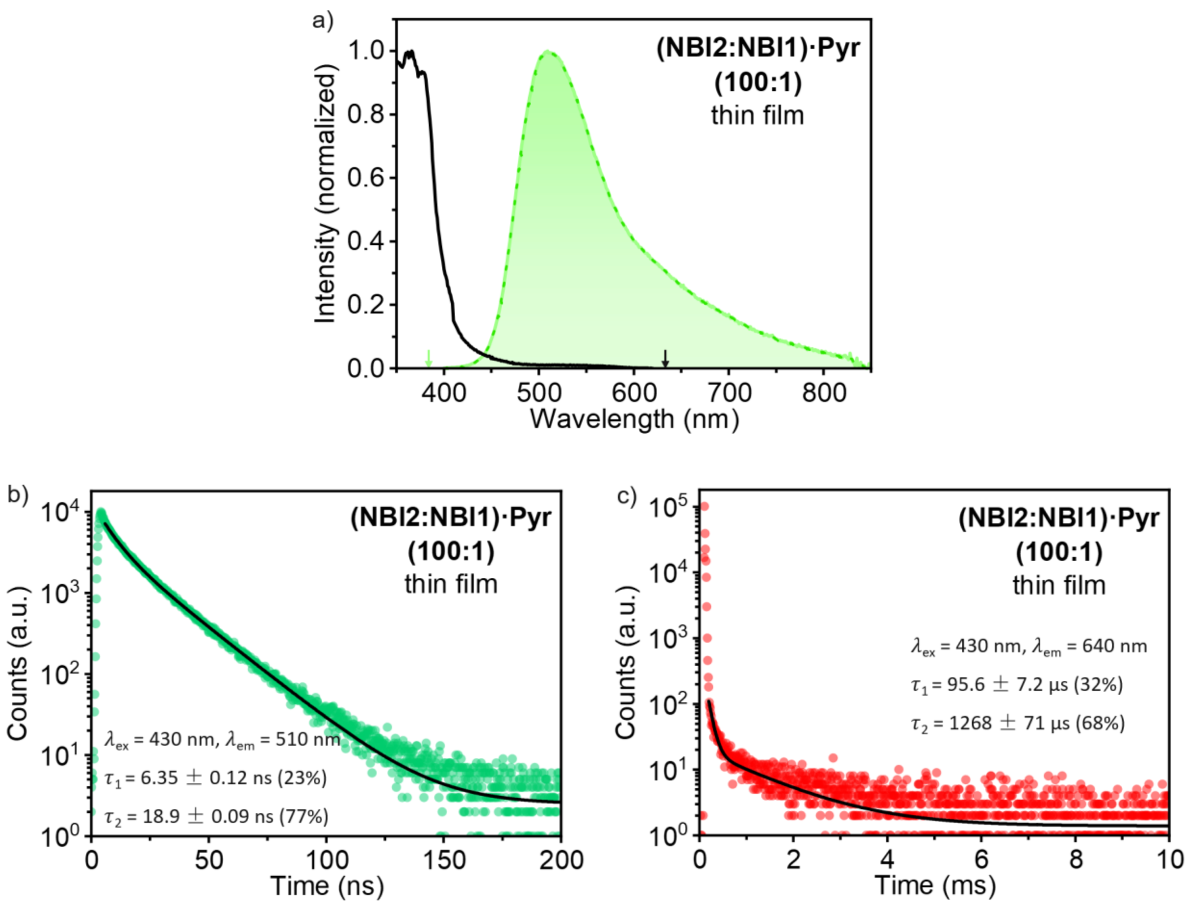

Figure S64. PL measurement of the mixture **(NBI2:NBI1)·Pyr** (LC host:guest, 5:1) with the ratio of LC hosts (**NBI2**:**NBI1**) 100:1 as thin film at room temperature. a) Normalized excitation (black line) and emission spectrum (green line) (λ_{em} = 640 nm, λ_{ex} = 380 nm). The arrows indicate λ_{em} of the excitation spectrum (black) and λ_{ex} of the emission spectrum (green). The emission spectrum was smoothed with adjacent-averaging of 5 points (green dotted line). PL lifetime decay (symbol, λ_{ex} = 430 nm) of b) the fluorescence (λ_{em} = 510 nm) and c) TADF (λ_{em} = 640 nm) component with the best fit (black line). The lifetime components, λ_{ex} and λ_{em} of the decay are given next to the graph.

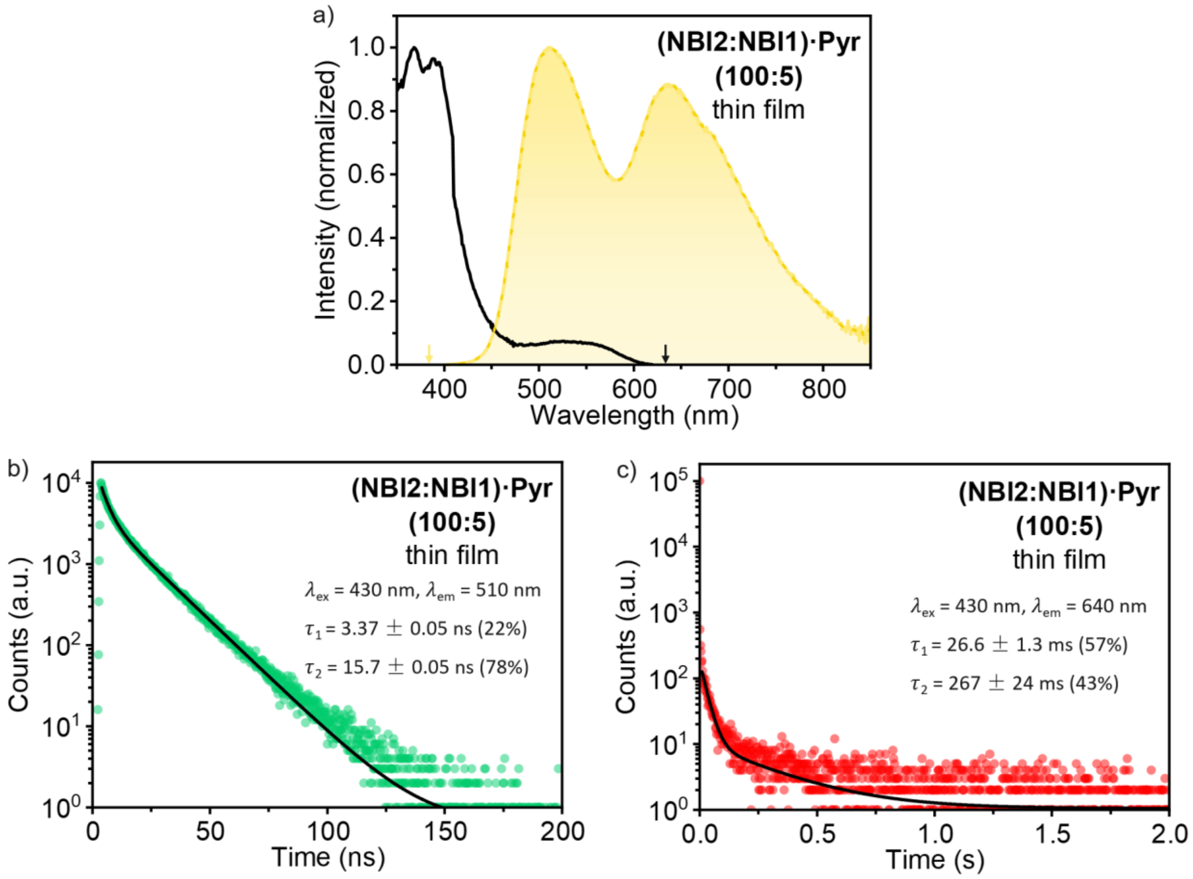

Figure S65. PL measurement of the mixture **(NBI2:NBI1)·Pyr** (LC host:guest, 5:1) with the ratio of LC hosts (**NBI2**:**NBI1**) 100:5 as thin film at room temperature. a) Normalized excitation (black line) and emission spectrum (yellow line) (λ_{em} = 640 nm, λ_{ex} = 380 nm). The arrows indicate λ_{em} of the excitation spectrum (black) and λ_{ex} of the emission spectrum (yellow). The emission spectrum was smoothed with adjacent-averaging of 5 points (yellow dotted line). PL lifetime decay (λ_{ex} = 430 nm) of b) the fluorescence (λ_{em} = 510 nm) and c) TADF (λ_{em} = 640 nm) component with the best fit (black line). The lifetime components, λ_{ex} and λ_{em} of the decay are given next to the graph.

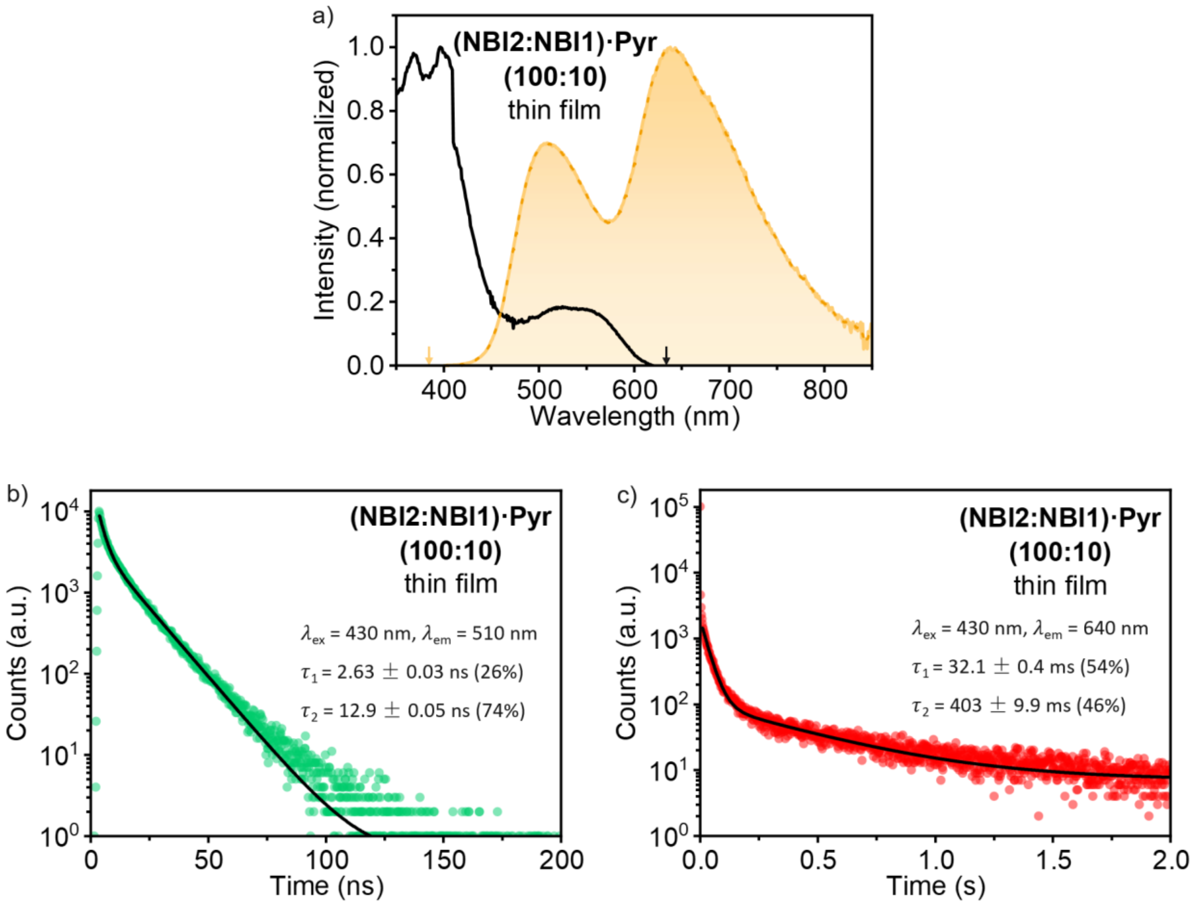

Figure S66. PL measurement of the mixture **(NBI2:NBI1)·Pyr** (LC host:guest, 5:1) with the ratio of LC hosts (**NBI2**:**NBI1**) 100:10 as thin film at room temperature. a) Normalized excitation (black line) and emission spectrum (orange line) (λ_{em} = 640 nm, λ_{ex} = 380 nm). The arrows indicate λ_{em} of the excitation spectrum (black) and λ_{ex} of the emission spectrum (orange). The emission spectrum was smoothed with adjacent-averaging of 5 points (orange dotted line). PL lifetime decay (λ_{ex} = 430 nm) of b) the fluorescence (λ_{em} = 510 nm) and c) TADF (λ_{em} = 640 nm) component with the best fit (black line). The lifetime components, λ_{ex} and λ_{em} of the decay are given next to the graph.

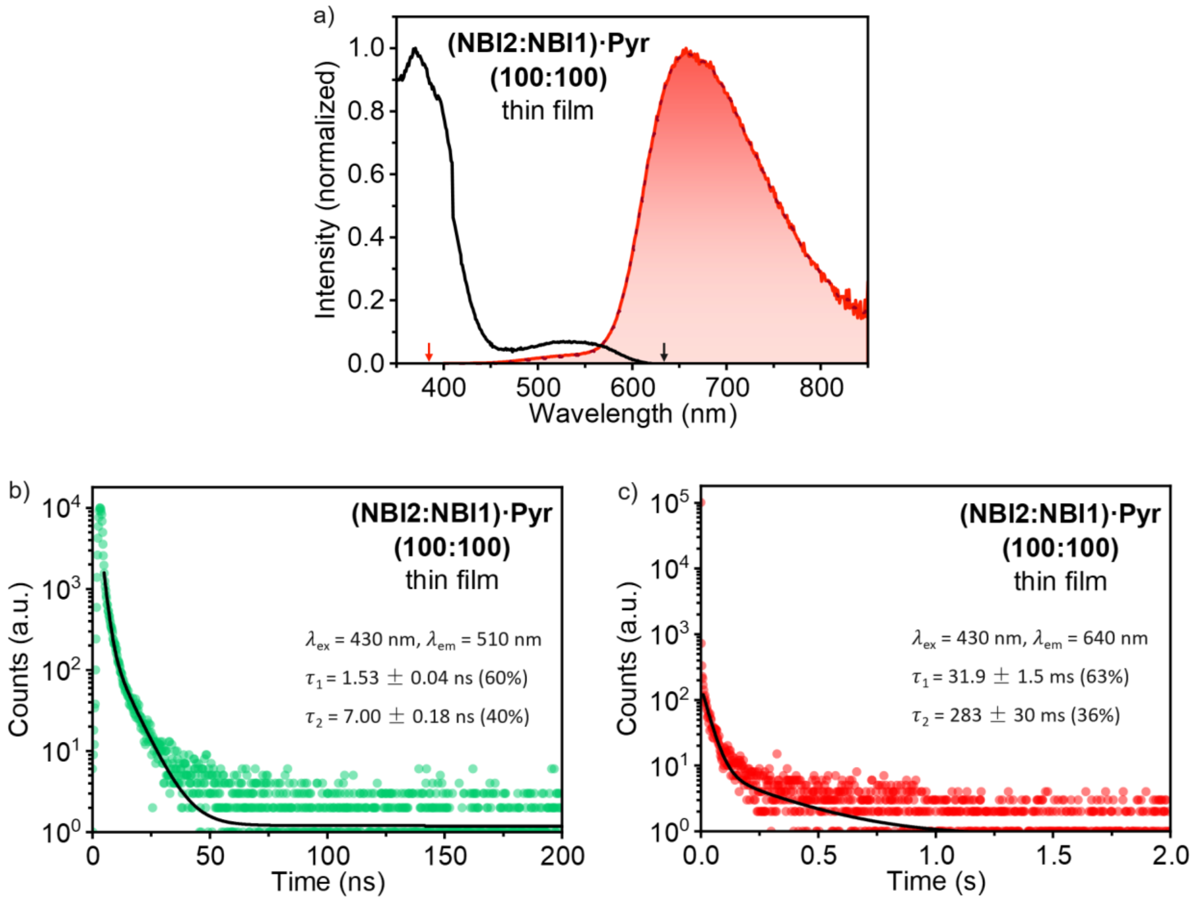

Figure S67. PL measurement of the mixture **(NBI2:NBI1)·Pyr** (LC host:guest, 5:1) with the ratio of LC hosts (**NBI2**:**NBI1**) 100:100 as thin film at room temperature. a) Normalized excitation (black line) and emission spectrum (red line) (λ_{em} = 640 nm, λ_{ex} = 380 nm). The arrows indicate λ_{em} of the excitation spectrum (black) and λ_{ex} of the emission spectrum (red). The emission spectrum was smoothed with adjacent-averaging of 5 points (red dotted line). PL lifetime decay (λ_{ex} = 430 nm) of b) the fluorescence (λ_{em} = 510 nm) and c) TADF (λ_{em} = 640 nm) component with the best fit (black line). The lifetime components, λ_{ex} and λ_{em} of the decay are given next to the graph.

4. NMR Spectra

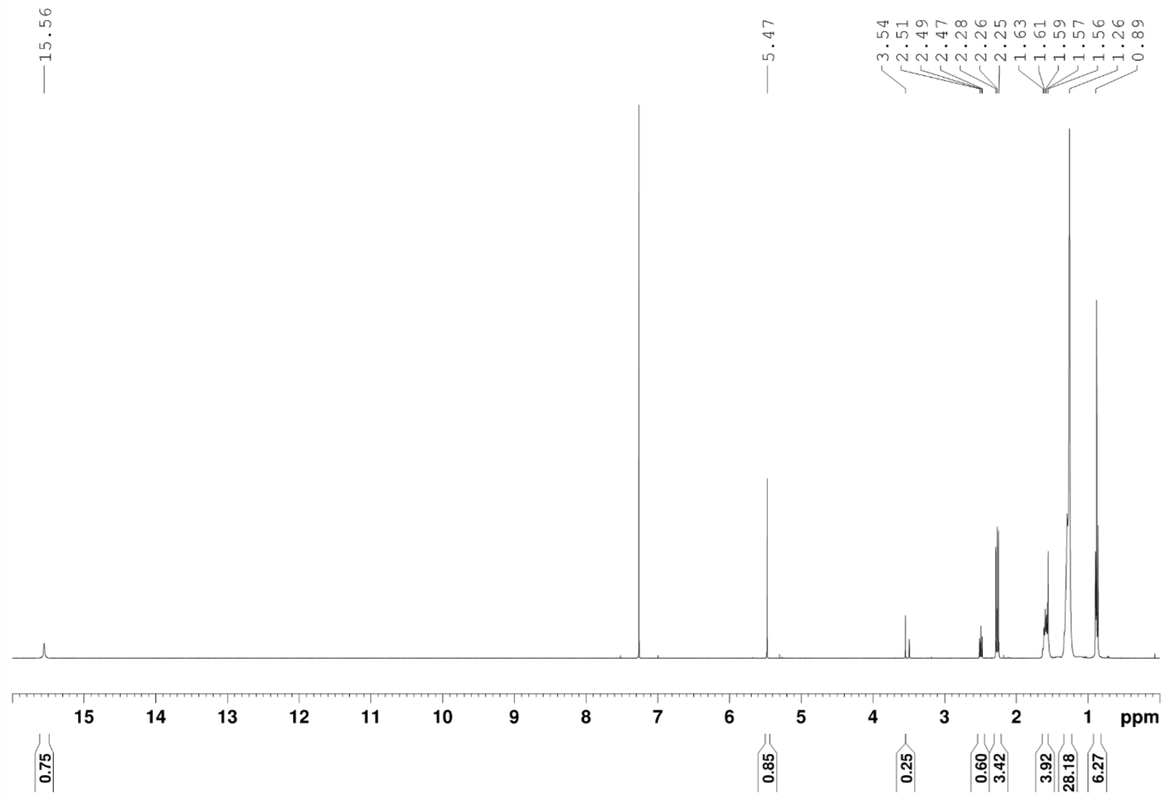

Figure S68. ^{1}H NMR spectrum (400 MHz, $CDCl_3$, 295 K) of 13-hydroxy-12-tricosen-11-one (**9**).

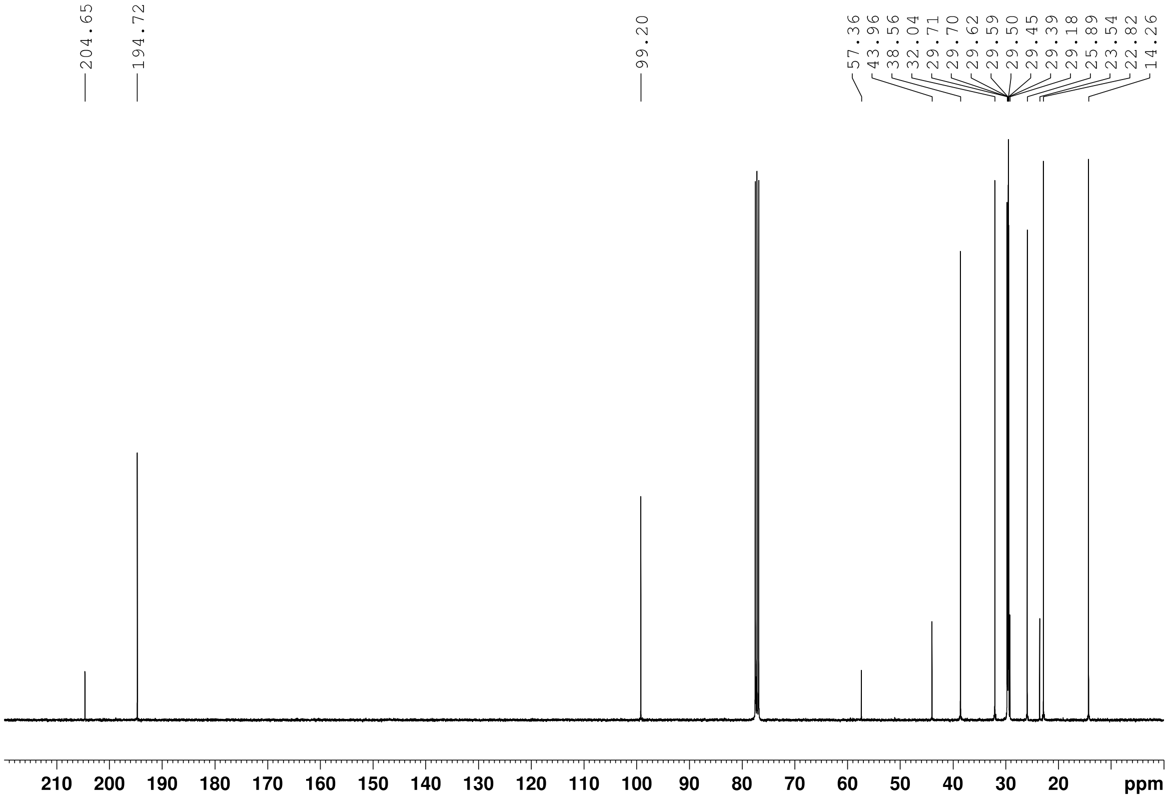

Figure S69. ^{13}C NMR spectrum (101 MHz, $CDCl_3$, 295 K) of 13-hydroxy-12-tricosen-11-one (**9**).

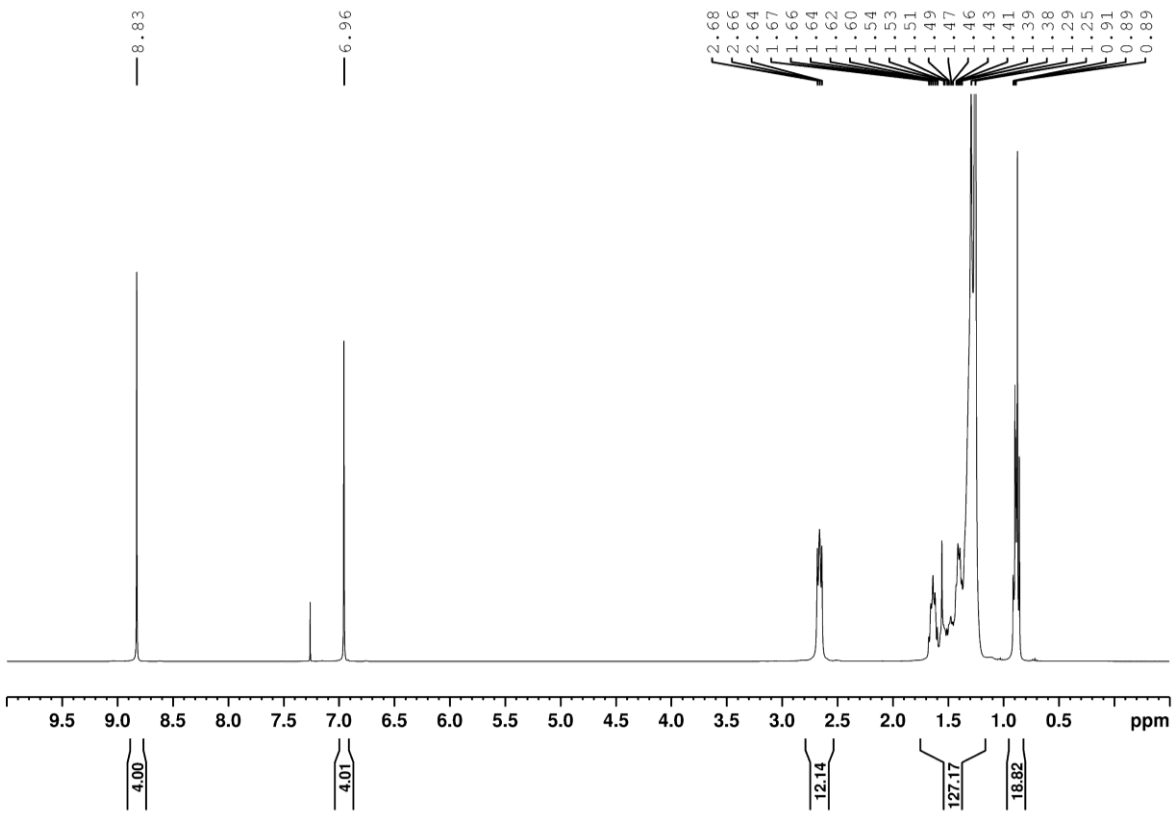

Figure S70. ^{1}H NMR spectrum (400 MHz, $CDCl_3$, 295 K) of **NBI1**.

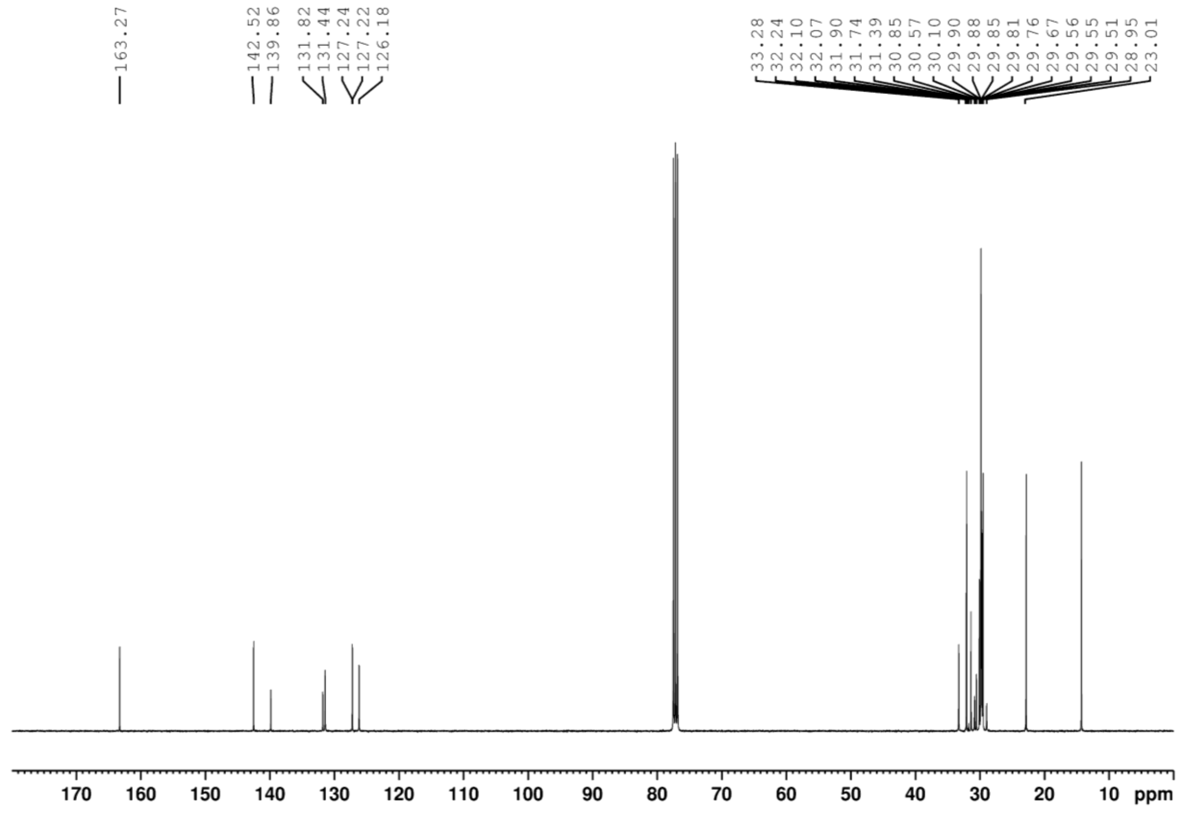

Figure S71. ^{13}C NMR spectrum (101 MHz, $CDCl_3$, 295 K) of **NBI1**.

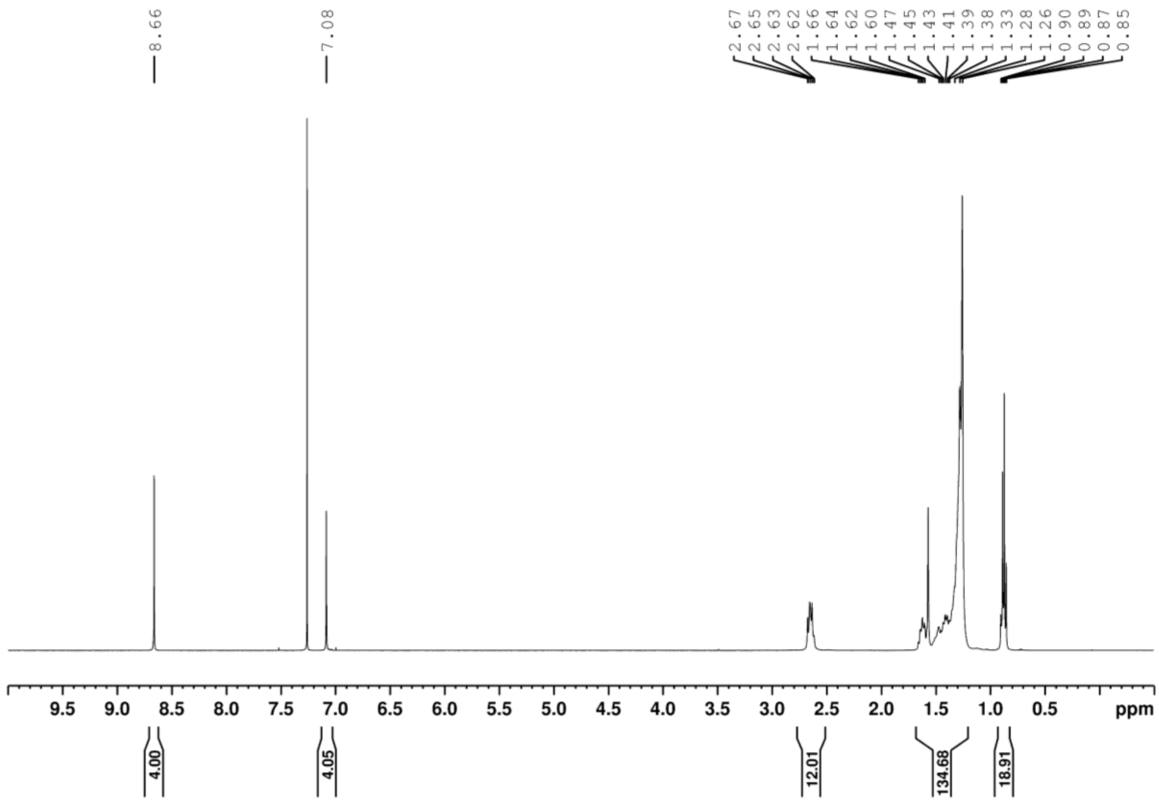

Figure S72. ^{1}H NMR spectrum (400 MHz, $CDCl_3$, 295 K) of **NBI2**.

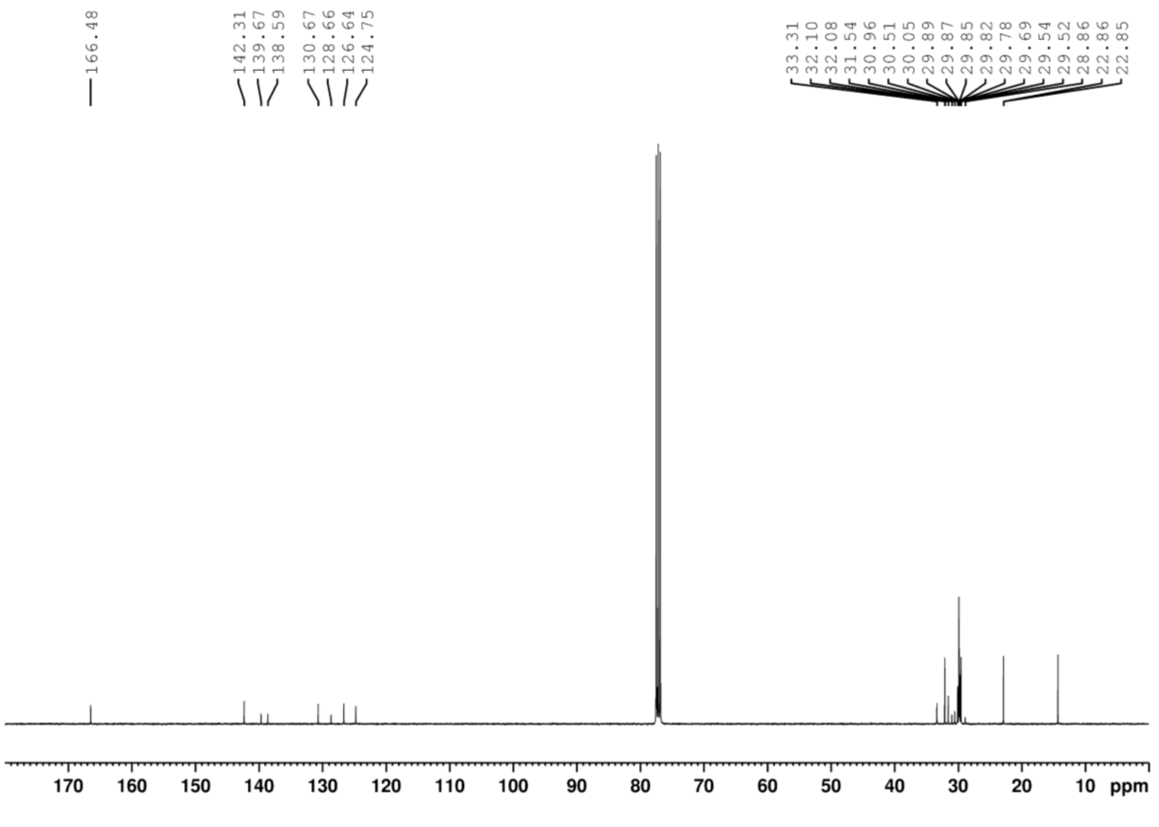

Figure S73. ^{13}C NMR spectrum (101 MHz, $CDCl_3$, 295 K) of **NBI2**.

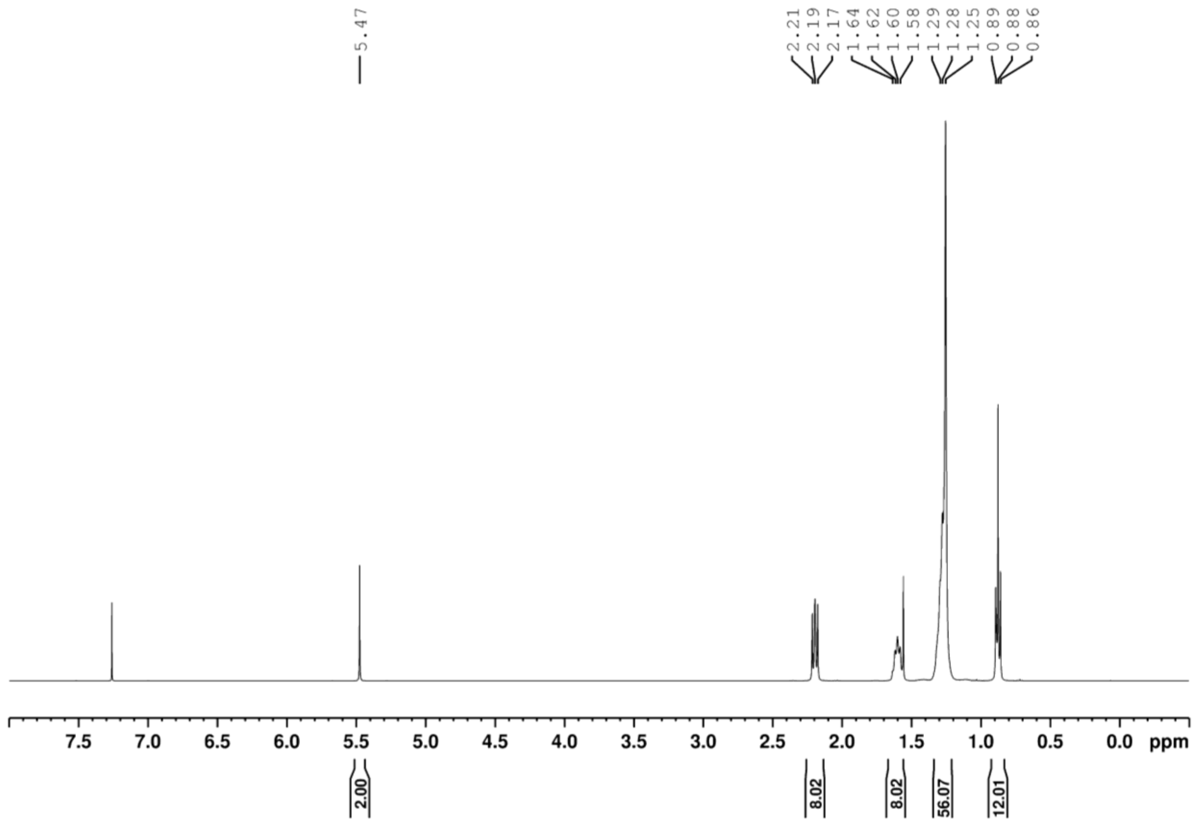

Figure S74. ^{1}H NMR spectrum (400 MHz, $CDCl_3$, 295 K) of **Pt1**.

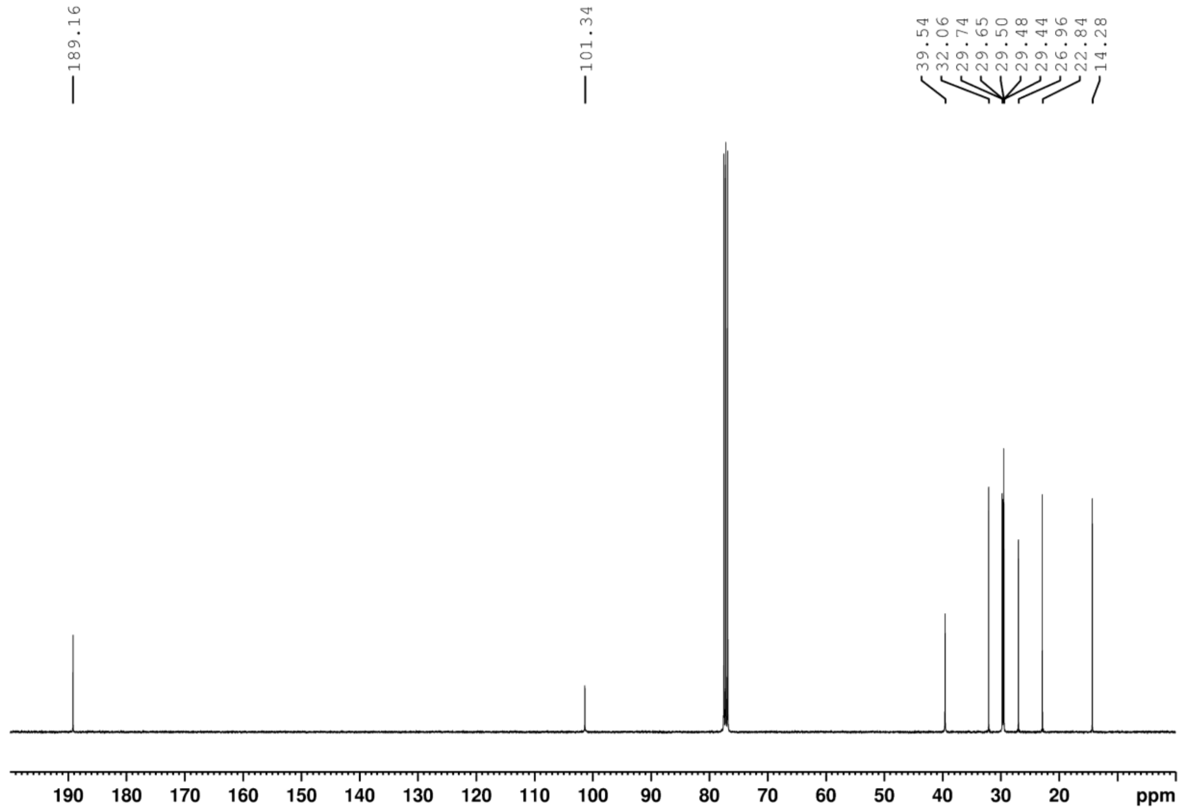

Figure S75. ^{13}C NMR spectrum (101 MHz, $CDCl_3$, 295 K) of **Pt1**.

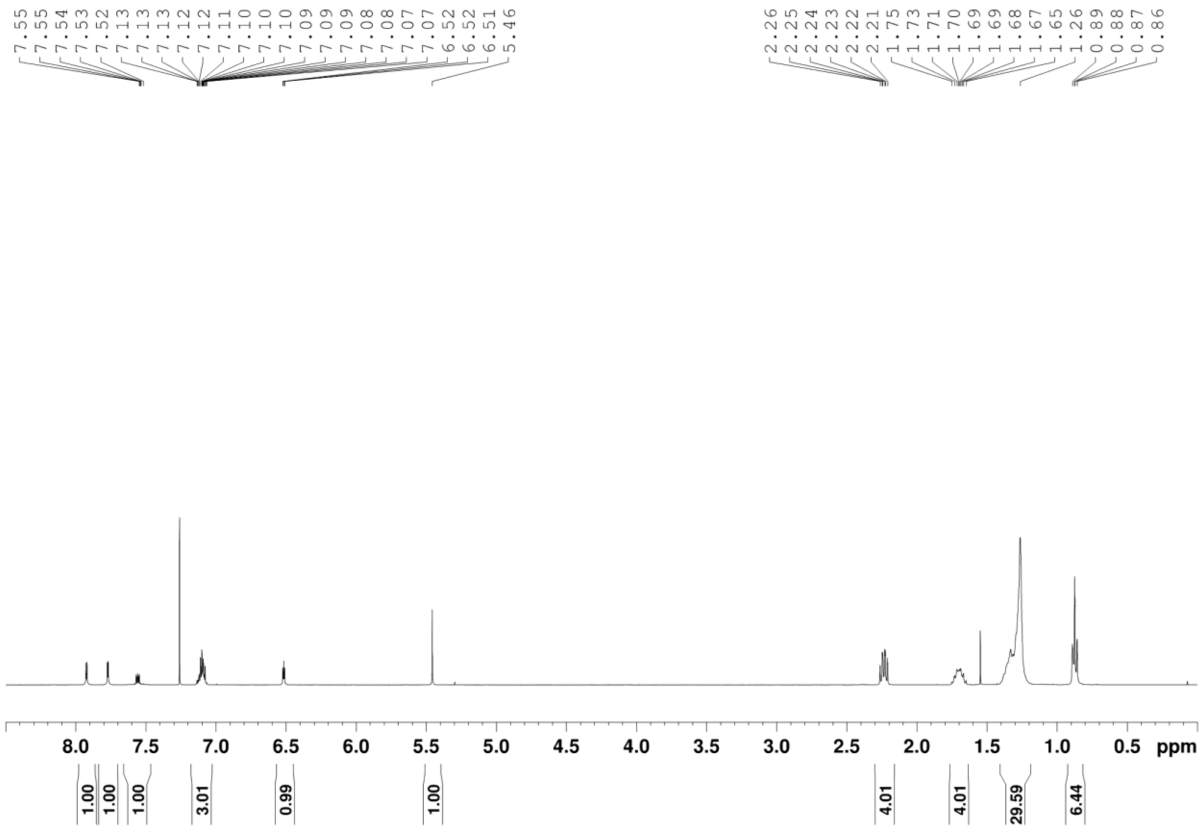

Figure S76. ^{1}H NMR spectrum (400 MHz, $CDCl_3$, 295 K) of **Pt2**.

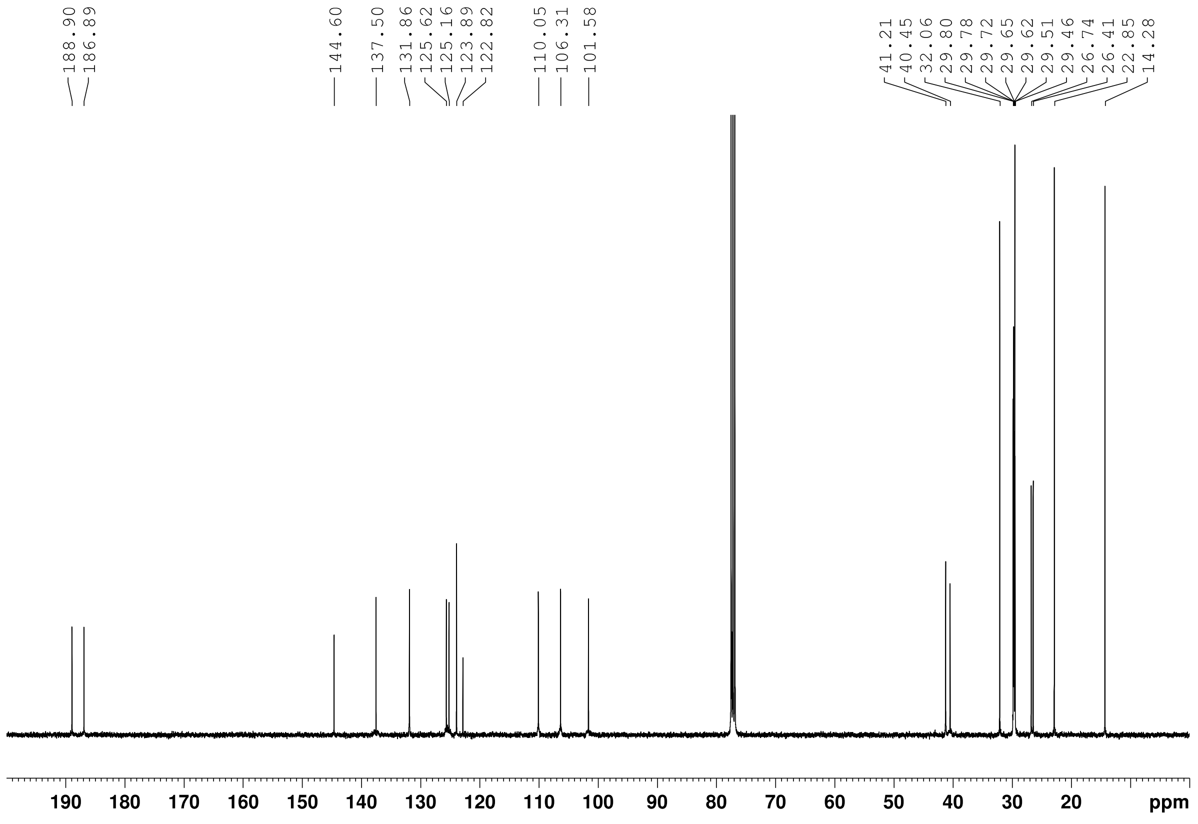

Figure S77. ^{13}C NMR spectrum (101 MHz, $CDCl_3$, 295 K) of **Pt2**.

5. Mass Spectrometry

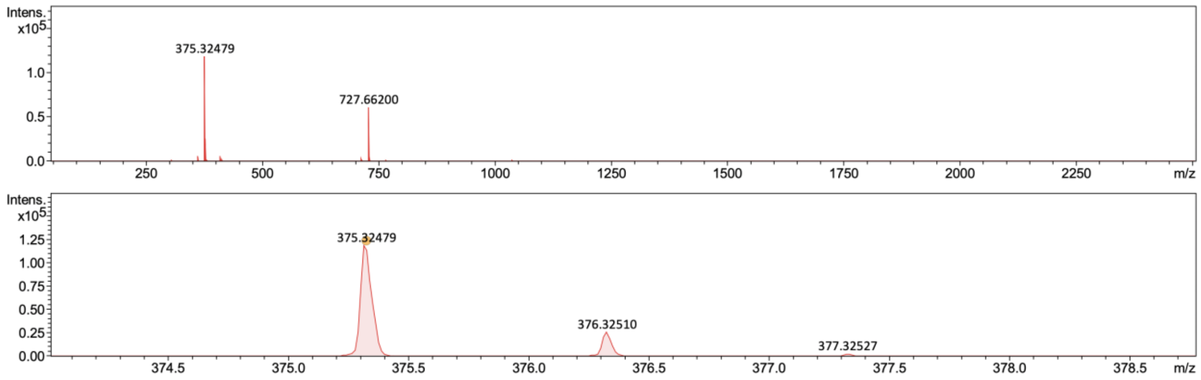

Figure S78. HRMS spectrum (ESI-TOF, pos. mode, $CHCl_3$/MeCN 1:1) of 13-hydroxy-12-tricosen-11-one (**9**).

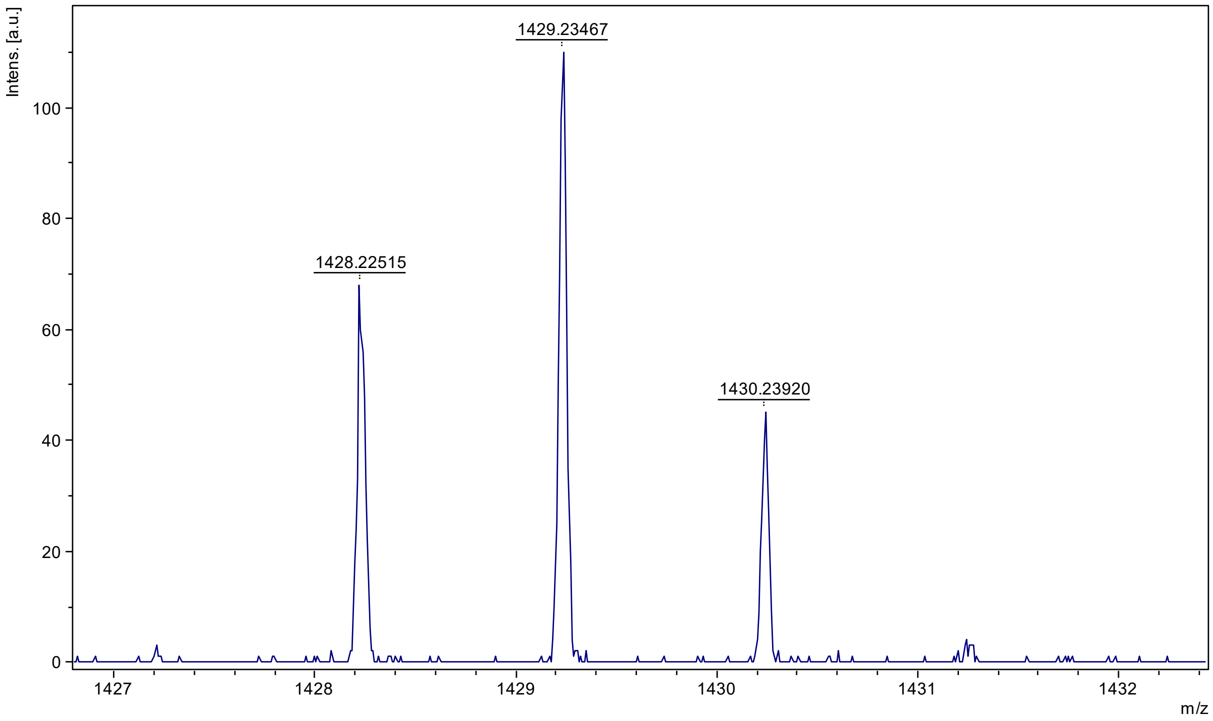

Figure S79. HRMS spectrum (MALDI-TOF, pos. mode, $CHCl_3$/DCTB, 1:3) of **NBI1**.

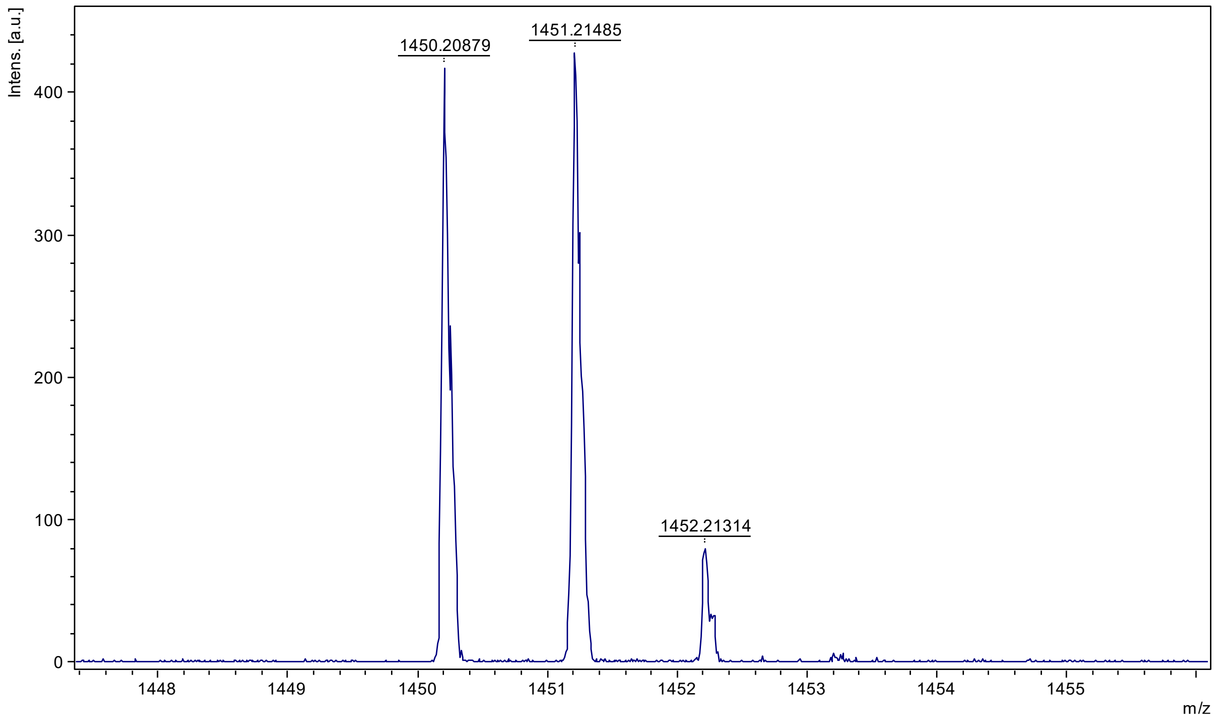

Figure S80. HRMS spectrum (MALDI-TOF, pos. mode, $CHCl_3$/DCTB, 1:3) of **NBI2**.

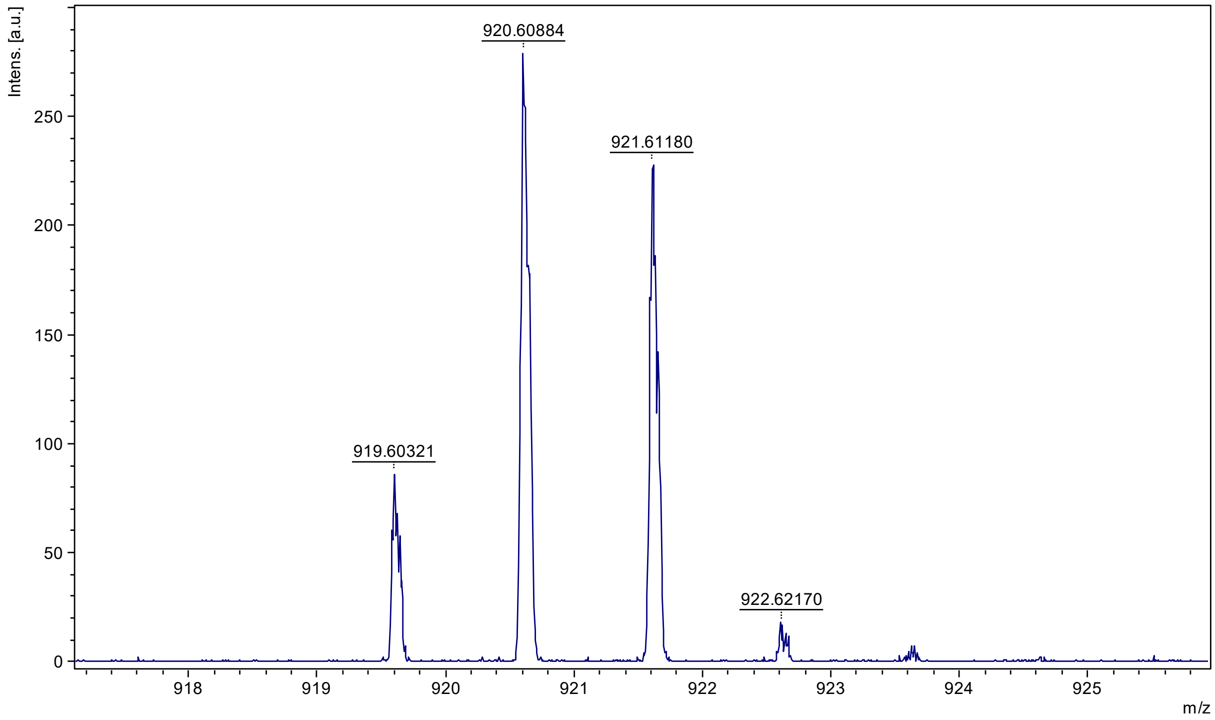

Figure S81. HRMS spectrum (MALDI-TOF, pos. mode, $CHCl_3$/DCTB, 1:3) of **Pt1**.

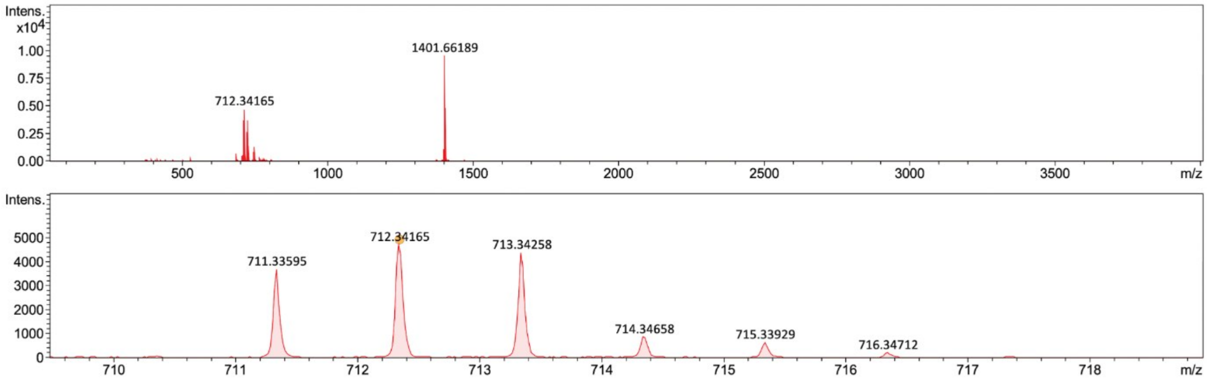

Figure S82. HRMS spectrum (ESI-TOF, pos. mode, $CHCl_3$/MeCN 1:1) of **Pt2**.

6. Supporting References

[S1] C. Chen, Z. Chi, K. C. Chong, A. S. Batsanov, Z. Yang, Z. Mao, Z. Yang, B. Liu, Nat. Mater. 2021, 20, 175–180.

[S2] a) G. M. Sheldrick, *Acta Crystallogr. A,* **2008**, *64*, 112–122, b) G. M. Sheldrick, *Acta Crystallogr. A,* **2015**, *71*, 3–8.

[S3] a) T. C. Huang , H. Toraya, T. N. Blanton, Y. Wu, *J. Appl. Cryst.* **1993**, 26, 180–184, b) T. N. Blanton, T. C. Huang, H. Toraya, C. R. Hubbard, S. B. Robie, D. Louër, H. E. Göbel, G. Will, R. Gilles, T. Raftery, *Powder Diffr.* **1995**, *10*(2), 91–95.

[S4] P. A. Heiney, Datasqueeze, Version 3.0.23, Pennsylvania, **2023**.

[S5] F. Weigend, R. Ahlrichs, Phys. Chem. Chem. Phys. 2005, 7, 3297–3305.

[S6] S. Grimme, S. Ehrlich, L. Goerigk, J. Comput. Chem. 2011, 32, 1456–1465.

[S7] R. van der Weegen, A. Teunissen, E. W. Meijer, *Chem. Eur. J.* **2017**, *23*(15), 3773–3783.

[S8] J. Föller, D. H. Friese, S. Riese, J. M. Kaminski, S. Metz, D. Schmidt, F. Würthner, C. Lambert, C. M. Marian, *Phys. Chem. Chem. Phys.* **2020**, *22*, 3217–3233.

[S9] J.C.E. Mertens, J.J. Williams, N. Chawla, *Nucl. Instrum. Methods Phys. Res. A* **2015**, 800, 82–92.
